# Supplementary material for: Selection and adaptive introgression guided the complex evolutionary history of the European common bean
Source: Nat Commun. 2023 Apr 5;14:1908. doi: 10.1038/s41467-023-37332-z (PMC10076260; doi:10.1038/s41467-023-37332-z)
Supplement: Supplementary file 1 — Supplementary Information [file 41467_2023_37332_MOESM1_ESM.pdf]

Selection and adaptive introgression guided the complex evolutionary history of the European common bean

Bellucci *et al*.

Supplementary Note 1. Background information on the common bean accessions and sample dataset description.

This research is part of the BEAN_ADAPT project, an international collaborative effort funded through the Second ERA-CAPS call, ERA-NET for Coordinating Action in Plant Sciences. The project aims to dissect the genetic basis and phenotypic consequences of the adaptation to new environments of the common bean (*Phaseolus vulgaris* L.), through the study of its introduction from the centers of domestication in the Americas and its subsequent expansion in Europe, as a recent and historically well-defined rapid adaptation event.

This work mainly focuses on landraces, which are plant genetic resources suitable for studying adaptation processes (see Cortinovis *et al.*[1] as review). Local varieties are adapted to specific agro-climatic conditions where they are cultivated and where they evolved under artificial and natural selection pressures over the years[2-8]. In this study, we analyzed 218 landrace accessions of common bean (BEAN_ADAPT Pv_core2) that were sampled to be representative of the geographic distribution and of the various environments where the species is cultivated all over Europe and in the centers of domestication in the Americas.

Whole-genome sequences from the 218 accessions were analyzed. The dataset is composed of 104 accessions (48%) from American centers of origin and domestication and of 114 accessions (52%) from Europe, that are representative of the geographic distribution of the species, and for most of them (74%) georeferenced information is provided (Supplementary Data 1). In particular, the European accessions are distributed as follow: 12 from Germany, 11 from Slovakia, 10 each from Italy and Spain, eight from Portugal, seven from Albania and France, six from Austria and Bulgaria, five from Greece and Romania, four each from Croatia, Georgia and Hungary, three from Sweden and Poland, two from Serbia, Slovenia and The Netherlands, and one each from Czech Republic, Turkey and Ukraine.

The American accessions have the following distribution per country: 27 from Mexico, 15 from Peru, 12 from Argentina, 12 from Chile, seven each from Brazil, Colombia, and Costa Rica, four each from, Ecuador and Honduras, three from Bolivia, two from Venezuela and El Salvador, and one each from Guatemala and Nicaragua.

Out of the total 218 accessions considered under this study, 89% (194) of all accessions are local varieties, 15 are commercial varieties, and nine domesticated accessions have no detailed information available regarding their biological status.

All the original seeds of the lines included in the BEAN_ADAPT Pv_core2 were collected from International Gene Banks or from individual Institutions/Organizations collections. In particular, 90 accessions are from the USDA Gene Bank (Pullman, Washington, USA), 79 from the IPK Gatersleben Gene Bank (Leibniz Institute of Plant Genetics and Crop Plant Research, Gatersleben, Germany), 38 from the Centro Internacional de Agricultura Tropical, Cali, Colombia (CIAT), and the remaining 13 accessions from common bean collection from other research institutions such as the University of California-Davis (UC-Davis, California, US), the Polytechnic University of Marche (UNIVPM, Ancona, Italy), the Università degli Studi di Sassari (UNISS, Sassari, Italy), the Nordic Gene Bank (NGB, Alnarp, Sweden) and the Agricultural Institute of Slovenia (AIS, Plant Gene Bank, Slovenia).

One hundred and ninety-nine lines of the 218 BEAN_ADAPT Pv_core2 accessions were multiplied and purified with at least three cycles of self-fertilization in controlled conditions, to obtain plants through single seed descent (SSD). For the 19 remaining accessions, one seed per accessions was sampled directly from bank original seeds provided by the donor.

Supplementary Note 2. Phenotyping

Phenotyping was performed using only the 199 accessions of the BEAN_ADAPT Pv_core2 from which we developed SSD lines. The phenotypic characterization was conducted in ten different environments: four field trials (in Italy and Germany locations) and six greenhouse experiments, from 2016 to 2018; overall, seven Long Day photoperiod, two Short Day and one intermediate photoperiod condition experiments were carried out (see also Supplementary Note 3 and 4). Experiment codes and conditions are summarized in Supplementary Table 1. In more detail, the four field trials were conducted during the summer of 2016 and 2017 in two experimental farms located in South of Italy at Villa d'Agri – Marsicovetere (Potenza) (experiment codes; I1FL6 and I1FL7), and in two experimental fields in Germany at Gatersleben IPK (D1FL6 and D1FL7); the six greenhouse experiments were conducted in 2016, 2017 and 2018 under controlled condition greenhouse in Golm (Potsdam, Germany) (D2GL6, D2GX7 and D2GS8), Potenza (Italy) (I1GS6 and I1GL7) and

Villaviciosa (Spain) (S1GL7).During the experiments, several phenotypic and phenological traits were recorded and for the present study, we focused on number of days from sowing until 50% of plants showed at least one open flower and growth habit, that has been recorded as determinacy *versus* indeterminacy on a single plant basis (i.e., the presence or absence of the floral bud at the terminal apex, respectively) in five environments. See also Supplementary Note 5 and 6 for more detailed information on classical and molecular phenotyping characterizations.

Supplementary Note 3. Field experiments

D1FL6 (Germany IPK Field LONG 2016) and D1FL7 (Germany IPK Field LONG 2017)

Phenotypic characterization was performed in Germany in the fields of IPK campus (latitude 51°49'19.74" N, longitude 11°17'11.80" E and altitude 111 m above sea level) in 2016 and 2017. The soil type in this location is fertile loess. The data of maximum and minimum temperature, relative humidity, rainfall and day length for the two years were obtained from the weather station on campus (Supplementary Table 2). The first field experiment in 2016 comprised 13 rows, each with 40 plots with a plot distance of 50 cm. Rows were organized in the following way: two adjacent rows distanced 50 cm from each other and 2 m from the next set of rows to allow gardening and plots evaluation. Each plot had three poles tied together to allow climbing of beans. Seeds were sown on 08th of June 2016. For each plot, five seeds were placed in a circle. The date of flowering time and the growth habit were evaluated during this experiment. Three lines did not germinate and accordingly they could not be evaluated. Field management, including irrigation, fertilizer application and pest control, followed normal agricultural practice.

A second field trial was conducted in 2017 as a randomized complete block design with four replicates. Due to the large field space required, three replicates were grown on the same field, while the fourth was grown on the adjacent field that was separate by a small street. Due to the different length of the second smaller field, the fourth replicate was organized in a different way. Each of first three replicates were organized in seven rows of 162 m length and 83 plots per row. While the fourth replicate had 10 rows with a length of 117 m and 60 plots each. However, in all four replicates the distance between rows was the same (3 m) as well as the plot size and distances within plot. The randomized trial followed a generalized alpha-lattice-design with block size of seven entries per block from which one was a control line. In the first three replicates on the larger field, the blocks within each replicate were organized across the seven rows (one cross section equal to one block), while in

the fourth replicate with ten rows, the blocks were meandering across the ten rows. Before planting, a fleece of 1 m width was laid out along each row to facilitate weed control and rows were pre-punched in the middle at 15 cm intervals. The distance between plots was 75 cm and each plot consisted of 9 plants place at 15 cm distance (according to the pre-punched holes). Seeds were sown on the 25th of May 2017 with one seed per pre-punched hole. Plants received common cultural and irrigation practices.

I1FL6 (Italy Field LONG 2016) and I1FL7 (Italy Field LONG 2017)

Phenotypic characterization was performed in South Italy in 2016 and 2017 on two experimental farms located in Villa d'Agri – Marsicovetere (Potenza, Italy). This is an inland intensive horticultural area, characterized by a temperate Mediterranean climate with cool winters and warm dry summers. The data of maximum and minimum temperature, relative humidity, and rainfall for the two years were obtained from the nearest weather station located at the farms, as shown in Supplementary Figure 1 and Supplementary Table 3. The 2016 field experiment was conducted at the experimental farm ALSIA (Azienda Agricola Sperimentale Dimostrativa 'Bosco Galdo') in Marsicovetere (Potenza, Italy - latitude 40°20′23.1″N, longitude 15°50′8.1″E, 592 masl) on a fertile coarse-lime soil.

Seeds for each accession were surface sterilized in 0.5% NaOCl for 10 min, rinsed, and germinated on jiffy peat pots filled with an adequate peat–pellet vermiculite. The field experimental layout comprised of 14 rows, each with 70-72 holes; the distance between rows was 1.5 m; the distance between holes (within the rows) was 0.9 m. For each accession, two single germinated seedlings were transplanted on the 27th of June 2016. The positions of the accessions were completely randomized. A plastic sheet was used along each row to facilitate weed control. Field management, including irrigation, fertilizer application and pest control, followed the normal agricultural practice.

A second field trial was conducted in 2017 at the experimental farm of the ITA (Istituto Tecnico Agrario "Rocco Scotellaro") in Marsicovetere (Potenza, Italy - latitude 40°19′56.7″N, longitude 15°49′28.4″E, 592 masl) on a fertile coarse-lime soil. The field experiment was conducted as a randomized complete block design with four replicates. Seeds were sown on the 22nd of June 2017 in single-row plots with nine seeds per plot (1.2 m between rows, 0.2 m between plants and 0.9 m between plots). A plastic sheet was used along each row to facilitate weed control. Field management, including irrigation, fertilizer application and pest control, followed the normal agricultural practice for both experiments.

Supplementary Note 4. Greenhouse experiments under controlled conditions

D2GL6 - Germany GOLM Greenhouse LONG 2016, D2GX7 - Germany GOLM Greenhouse intermediate 2017 and D2GS8 - Germany GOLM Greenhouse SHORT 2018

Seeds of each SSD line were scarified and germinated at room temperature in Petri dishes. When the radicle had just emerged, germinated seeds were planted in 20 (diameter)*20 (height) cm pots in a climate-controlled greenhouse. Three experiments with three biological replicates per accession were conducted, using a randomized complete block design, in greenhouse under controlled conditions at the Max Planck Institute of Molecular Plant Physiology, Golm, Germany as separately as the following:

(i) Long day conditions experiment (D2GL6): seedlings were grown under 8/16 h dark/light regime with 400-500 µmol m-2 s-1 photon lux density (LED light), 70% humidity and 24/20 °C day/night temperature;

(ii) Short day conditions experiment (D2GS8): seedlings were grown under 16/8 h dark/light regime with 400-500 µmol m-2 s-1 photon lux density (LED light), 70% humidity and 24/20 °C day/night temperature;

(iii) Intermediate day conditions experiment (D2Gx7): seedlings were grown from end of January till May 2017 under 70% humidity and 24/20 °C day/night temperature conditions. Artificial light has been always provided for eight hours per day in the greenhouse to support the plant growth, and when the day length exceeded the eight hours, no cover and protection from natural day light have been provided (the natural light conditions from January to May in Potsdam-Golm [coordinates 52.41568, 12.96881] ranged from about 8 to 16.30 hours).

During the three experiments plants were monitored on a daily basis for flowering time and growth habit. At the end of the experiments, pods were harvested and dried at room temperature.

I1GS6 (Italy Greenhouse SHORT 2016) and I1GL7 (Italy Greenhouse LONG 2017)

The common bean accessions were grown in two different experiments (short-day and temperate/long day conditions) in the Experimental Greenhouse at the Università degli Studi della Basilicata in Potenza, South Italy (latitude 40°38′51.7″N, longitude 15°48′31.7″E).

In both experiments, two seeds for each accession were individually germinated on jiffy peat pots filled with an adequate peat–pellet vermiculite. After four days, germinated seedlings were transplanted into 24 cm (diameter) × 26 cm (height) pots (one seedling per pot), filled with peat soil. Transplanting was performed on the 31st of October 2016 for the short-day experiment, and on the 22nd of February 2017 for the temperate/long day experiment. The experiment was a randomized complete block design with two plants per accession. All plants were watered regularly three times per day with 400 ml of tap water. The experiments were carried out under natural lighting in a greenhouse, with the air temperature kept between 18 and 24 °C, and the relative humidity between 40 and 60%.

Serida Spain 2017 - S1GL7

Accessions were grown in Villaviciosa, Spain (43° 28′ 53″ N, 5° 26′ 7″ W) during the spring 2017 (March-November) under natural lighting in a greenhouse (long day conditions). Plants were grown in 7.5 liters pots containing 80% peat and 20% perlite, as well as 20 g per pot of fertilizer (OSMOCOTE© EXACT 3M 16-9-12+2MgO+TE). A pot per accession with three individual plants was used and a randomized complete block design was applied for pots distribution. Plants were daily irrigated considering the state of development and temperature.

Supplementary Note 5. Molecular phenotyping

Tissues of the BEAN_ADAPT Pv_core2 accessions were harvested from the long day-conditions experiment with three biological replicates; first trifoliate fully expanded leaves were used. The frozen samples were subsequently powdered in a Mixer Ball mill (Retsch, MM 300) using 5 mm steel balls (Th. Geyer Berlin) for 1 min at 25 Hz and stored at -80°C. Secondary metabolites measurement was performed according to Perez de Souza *et al.*[9]. Briefly, 50 mg frozen powder were extracted in 80% methanol, and subjected to LC-MS analysis, mass spectrometry was performed on a Thermo Exactive machine following separation in an UPLC Waters Acquity machine using a Waters column HSS T3 C18 (100 mm l. x 2.1 mm i.d. x 1.8 µm particle size), with column temperature control set at 40°C. A 20 minute elution gradient at 0.4 mL.min-1 using UPLC MS-grade water + 0.1% formic acid (A) and acetonitrile (UPLC MS-grade) + 0.1% formic acid (B) was applied. Mass range was set from m/z 100 to m/z 1500. Ionization was performed using an ESI source with capillary conditions set to 3 kV and 200 °C, drying gas at 350 °C, sheet gas flow and auxiliary gas flow at 60 and 20 units, respectively, and skimmer and tube lens voltages at 25 V and 130 V, respectively. For non-targeted

metabolomics, chromatograms were processed and peak detection and integration were performed using REFINER MS® 10.0 (GeneData, http://www.genedata.com).

In order to explore the molecular phenotypic diversity among the 199 common bean accessions, we performed a non-targeted metabolic fingerprinting analysis using the high-throughput LC-MS analysis on the leaf materials collected from long day experiment. Using this approach, without assigned compound names to mass spectra, we were able to detect more than 20,000 mass features (not included isotopic peaks). In order to select reliable and robust mass features, we applied a filter to exclude mass signals which were not detected in ≥ 50% of the samples and/or having ≤ 1000 peak intensity. This resulted in 31795 mass features that were subjected to heritability (h^2_B) analysis using the REML model (JMP software, Version 8. SAS Institute Inc., Cary, NC, 1989-2019) setting genotype and Continent (i.e., America and Europe) with random effect. Accessions with only one replicate were excluded from the analysis. The heritability was calculated based on 190 accessions (94 and 96 from Americas and Europe, respectively). As a result, 8417 mass features with $h^2_B \geq 0.40$ were left and used to preform Principal Component Analysis (PCA) (GeneData, http://www.genedata.com). Results (Supplementary Figure 2) showed that the first and the second components explained 25.7 % and 7.7 % of metabolic diversity across different groups, respectively.

Supplementary Note 6. Classical phenotypic characterization

As previously reported in the Supplementary Note 2, the growth habit trait was registered in five environments as determinate/indeterminate (i.e., presence/absence; code GH). Flowering time was registered as days from sowing to flowering (DTF) when 50% of plants showed at least one open flower, and it was registered in all the ten different environments. For eight of the ten environments, replicates (varying from two to four per environment) were available for each plant. A DTF value of 300 days was assigned to accessions that did not reach flowering until the end of the trial. Photoperiod sensitivity (PS) was calculated as the ratio between DTF in long day experiments and DTF in a short-day experiment; I1GS6 was used as the reference short-day experiment.

Main descriptive statistics of the different phenotypic traits were calculated by using R (https://cran.r-project.org/) or JMP 7.0.0. The restricted maximum likelihood (REML) model as implemented in JMP 7.00 (SAS Institute, Inc. 2012) was used to calculate the least square means (LSM) and the Best

Linear Unbiased Predictors (BLUPs) of each genotype. REML model was also used to calculate the broad sense heritability (h^2_B) for each quantitative trait, by assuming genotypes and environments as random effects. Distribution of flowering time in each environment and Pearson's pairwise correlation among environments were calculated using *corrplot* and *PerformanceAnalytics* R packages[10-11].

Flowering time varied widely among accessions and locations in which some non-flowering plants were registered across the American accessions (Supplementary Figure 3, Supplementary Table 4). The only environment in which all the accessions reached flowering was the short-day greenhouse trial in Italy (I1GS6). The flowering intervals (DTF range between the first and the last flowering accession) varied between 22.7 days at I1GS6 and 102.6 days at D1FL7. American accessions always showed wider flowering intervals in respect to the European ones (Supplementary Table 4). To obtain a flowering synthetic index (FI) related both to flowering dates and photoperiod sensitivity, the data registered across the ten environments were subjected to Principal Component Analysis (PCA). Considering that variable missing data points were present in the different environments, flowering and photoperiod data were firstly imputed by using PHENIX R package, a Bayesian multiple phenotype mixed model developed to impute missing phenotypes in related samples[12] (https://mathgen.stats.ox.ac.uk/genetics_software/phenix/phenix.html). The PCA was then performed on the data and the first five principal components (PCs), cumulatively explaining the 87.3% of the total variance, were retained as representative of the flowering time variation of the BEAN_ADAPT Pv_core2 (Supplementary Figure 4).

Supplementary Note 7. Sequencing

The total DNA for each of the 199 SSD lines was extracted from frozen young leaves of plants that were grown in greenhouse, and directly from seeds for each of the 19 accessions provided by the gene bank. The unexpanded young leaves of single plant genotype were frozen and grinded using liquid nitrogen and TissueLyser (Qiagen, Hilden, Germany) with tungsten beads. The uniformly disrupted and homogenized tissues were used for extracting nuclear DNA according to the QIAGEN protocol of the Qiagen DNeasy Plant Mini Kit (#69106 Qiagen, Hilden, Germany).

The purity and quantity of the DNA were determined using NanoDrop 2000 (Thermo Fisher Scientific, Waltham, MA) and Qubit 2.0 fluorometer (Thermo Fisher Scientific). Each DNA sample

was sheared with Covaris E220 to fragment sizes of approximate 550 bp. PCR-free libraries were constructed according to manufacturer's instructions (KAPA HyperPrep Kit PCR-free). Paired-end sequencing libraries were sequenced on Illumina HiSeq2500 or HiSeq4000 sequencers. Libraries were labeled with different barcodes and 10-12 libraries were loaded on each lane.

Supplementary Note 8. Mapping and variant calling

Paired-end reads were mapped to the common bean reference genome (V2.0)[13] using BWA-mem (V0.7.15) with parameter "-M"[14]. The alignments were converted into BAM format with SAMtools software (v1.3.1)[15]. The longer reads from HiSeq4000 runs were trimmed to 126 bp before mapping. BAM files from different runs of the same accession were merged with SAMtools software[15]. The average data yield is 44.4 million 126 bp reads/sample. The mapping metrics of each accession were collected using "CollectWgsMetrics" and "CollectMultipleMetrics" commands in Picard software. The average mapping rate and mapping depth across all samples is 0.98 and 6.5-fold, respectively (Supplementary Data 2, Supplementary Figure 5).

Two methods, SAMtools[16] and GATK (V3.6)[17-18], were used for variant calling. In SAMtools, duplicated reads were removed with "rmdup". Aligned reads were sorted according to their coordinate on the chromosomes. SNPs were discovered with "mpileup" for filtered high quality alignments (mapping quality > 10, -q 10) and bases (base quality > 20, -Q 20) and then genotyped with BCFtools[16]. With GATK, duplicated reads were sorted and filtered with Picard (V2.4.1) (http://broadinstitute.github.io/picard) for sequencing duplicates. Variants were then discovered using "HaplotypeCaller" mode for each accession separately. Then the joint genotyping across all accessions was performed using "GenotypeGVCFs" mode. SNPs were extracted using the "SelectVariants" mode and pre-filtered using the "VariantFiltration" mode with the recommended parameters for hard filters (--filterExpression "QD < 2.0 || MQ < 40.0 || FS > 60.0 || SOR > 3.0 || MQRankSum < -12.5 || ReadPosRankSum < -8.0"). The unmapped reads were collected from each BAM file and mapped to the *P. vulgaris* chloroplast genome (NCBI Entry: NC_009259) with BWA-mem with the same parameters for each sample. Chloroplast SNPs were produced following the same pipeline. A total of 16,402,828 and 14,286,271 SNPs were identified by SAMtools and GATK, respectively; 8,864,845 overlapping SNPs were left after pre-filtering for SNP qualities with GATK. The genotypes of SNP sites in each sample were summarized using "vcfstats" command in RTG Tools[19]. The percentage of missing genotypes varied from 0.5% to 18.6% in AM population and from

2.0% to 18.8% in EU population (Supplementary Data 3, Supplementary Figures 6, 7, and 8). The average percentage of missing genotypes is 5.4% in AM population and 5.6% in EU population. The percentage of heterozygous SNPs varied from 0.3% to 12.8% in AM population and from 0.8% to 10.8% in EU population (Supplementary Data 3, Supplementary Figures 6, 7, and 8). The average percentage of heterozygous SNPs is 3.1% in AM population and 2.8% in EU population. The percentage of homozygous SNPs varied from 0.02% to 34.8% in AM population and from 6.5% to 34.8% in EU population (Supplementary Data 3, Supplementary Figures 6, 7, and 8). The average percentage of homozygous SNPs is 20.4% in the AM population and 17.7% in the EU population.

Supplementary Note 9. SNPs annotation

Overlapping SNPs reported by both methods (see Supplementary Note 8) were then annotated with snpEff (V4.3s)[20]. Only chromosomal SNP sites reported by both methods were kept and the genotypes produced by GATK were selected. SNPs whose genotypes were reported in more than 50% of the samples and with minor allele frequency (MAF) higher than 0.05 (VCFtools parameters --minDP 3 --max-missing 0.5 --maf 0.05) were selected. SNP sites whose proportions of heterozygous genotypes were higher than 0.01 were removed. Finally, 3,349,009 SNPs were retained for effect annotation. The snpEff annotation database was built with the *P. vulgaris* genome annotation (V2.1). Only the canonical transcripts (snpEff parameter "-canon") were used to annotate variants and the remaining snpEff parameters were left as default. The majority of SNP effects were found in intergenic (45.82%), upstream (22.86%) and downstream (20.71%) regions, respectively (Supplementary Figure 9). The SNP effects found in intronic regions was 7.07% of the total effects while the percentage of SNP effects located in exonic regions was 2.20% (Supplementary Table 5).

Supplementary Note 10. Indel calling and annotation

Only indels called by GATK were used (see also Supplementary Note 8). The indels were first filtered to remove low quality sites with the following parameters (--filterExpression "QD < 2.0 || ReadPosRankSum < -20.0 || InbreedingCoeff < -0.8 || FS > 200 || SOR > 10.0") with GATK. In total, 2,804,880 raw indels were identified with GATK and 2,769,975 were retained after pre-filtration with GATK. Indels were further filtered with the same setting as SNPs for effect annotation. Finally, 853,371 indels remained. The percentages of indel effects found in intergenic, upstream and downstream were 42.22%, 25.80%, 23.18%, respectively (Supplementary Figure 10). The indels

effects found in intronic regions was 7.07% of the total effects while the percentage of indel effects located in exonic regions was 0.33% (Supplementary Table 6).

Supplementary Note 11. Identification of copy number variations

The copy number variations (CNVs) were identified with CNVcaller software[21]. Briefly, the reference genome sequences were partitioned into 3,000 bp windows for duplicated window search. The windows were aligned to the reference genome with BLASR software[22] and linked windows were identified with the scripts supported in the CNVcaller package. BAM files were preprocessed with Picard software for sorting and marking duplicates and then indel regions in the BAM files were realigned with GATK. CNV windows were detected by the CNVcaller with the following parameters "-f 2 -h 5 -r 0.15". Windows identified as homozygous gain/loss in five or more individuals were retained (as defined by parameter -h 5). The merged CNV windows were genotyped with CNVcaller among individuals and the CNVs were stored in VCF format and tsv format, respectively.

Supplementary Note 12. Analysis of genetic diversity and population structure: Additional quality filtering of variants

In order to measure the levels of genetic diversity, specific additional filters were applied to the SAMtools/GATK overlapping SNP callset described in Supplementary Note 8. Only the genomic positions having a high probability to be polymorphic (QUAL >=30) and a global depth of coverage between 1/3 and 4 times the mean value, were retained using VCFtools[23]. Additionally, individual genotypes called using two reads or less, were marked as missing data. This first step of filtering decreased the number of polymorphic positions to 8,816,890 SNPs. The second round of filtering was applied to exclude (i) all the singletons and private doubletons identified by the "--singletons" VCFtools command, (ii) SNPs with more than 5% of missing data, and (iii) not biallelic SNPs. The retained high-quality set of SNPs, anchored to chromosomes from 1 to 11, was composed of 2,370,523 variable positions that were imputed and phased using Beagle[24].

Supplementary Note 13. Population Structure: Ancestry inference

A population structure analysis was conducted using the maximum likelihood approach implemented in ADMIXTURE v1.3[25]. The information contained in multiple independent SNPs is combined to reconstruct whole genome ancestry for each individual given a prior defined number of ancestral populations K. To satisfy the assumption of independence between markers, and since the haplotype phase is not used in the computations, the unphased variants were filtered taking one SNP every 250kb using the VCFtools "--thin 250000" option and then converted into ped format. In this way, sporadic errors due to the phasing and imputation steps should not affect the analysis. To determine the optimal number of ancestral populations (K) supported by the data, ADMIXTURE was run varying K from 1 to 20, enabling the computation of the cross-validation error using 20 replicates. The best K was then selected according to the lower cross-validation error. The analysis was performed independently over the whole sample of American and European (n=218) accessions or using the American (AM, n=104) accessions only (see also Supplementary Note 14 and 15). The Q scores from the ADMIXTURE analysis at K=2, computed over the whole sample, were used to set up two supplementary runs including individuals from the Mesoamerican (AM_M, n=53) or the Andean (AM_A, n=51) gene pools, selected by having > 80% of their genome composed of alleles from the respective pool. This approach was designed to deal with possible cryptic population structure within pool as in Rossi *et al.*[26] and Bitocchi *et al.*[27,28].

Based on the cross-validation plot, the analysis of all sequenced accessions (America and Europe) supported the existence of two groups, showing the biggest reduction in the cross-validation error moving from one to two ancestral populations (Supplementary Figure 11). Focusing on American accessions (Europeans excluded), three ancestral components were identified in America, two in Mesoamerica and four in the Andes, respectively.

Supplementary Note 14. Genetic structure in the whole sample (America and Europe)

The ancestry plot of American accessions (AM) at K=2 (Supplementary Figure 12), highlighted two clusters composed by almost pure accessions (q > 99%) corresponding to the Cluster1 (red) and Cluster2 (blue). The Cluster1 (red) prevailed among accessions geographically located in Mesoamerica whereas the Cluster2 (blue) was observed more frequently among Andean ones. Within America, 53 individuals had more than 85% of their genome composed by the Mesoamerican cluster (red), 39 of those came from Mesoamerica and 14 from South America, according to geographic

information. On the other side, among the 51 individuals enriched for the Andean component (P.And > 0.8), 41 came from South American and only 10 from Mesoamerican area.

Despite the high frequency of pure individuals, clear signals of gene flow between pools were present, specifically ECa084, ECa094, ECa055, ECa099, ECa101 had from 3 to 15% of Andean contribution to their Mesoamerican genome whereas the ECa046, ECa011, ECa051, ECa002 and ECa041 accessions exhibited from 5 to 19% of Mesoamerican alleles within their Andean genetic background (Supplementary Figure 12).

The accessions geographically located in Mesoamerica were preferentially attributed to the Mesoamerican gene pool (red) with the exception of one accession from Costa Rica (ECa027) and 5 accessions from Mexico (ECa013, ECa014, Eca029, Eca032 and ECa033) that were assigned to the Andean gene pool (Supplementary Figure 13). A more heterogeneous situation was present in South America, where the Mesoamerican and the Andean ancestry components were present in all the sampled geographic areas. This peculiar distribution was not observed in Argentina and Bolivia where the accessions were mainly attributed to the Andean gene pool (with the exception of accession ECa102).

Pervasive admixture was detected in almost all European accessions (Supplementary Figure 12) but with a variable proportion of their genome assigned to have a Mesoamerican or an Andean origin. Specifically, the analysis of the admixture proportions revealed that 62% of the European accessions had more than 50% of their genome attributed to the Andes. Within this group (denoted as EU_A), the 89% of the members showed less than 20% of contribution from the Mesoamerican gene pool. In the remaining 38% of the European individuals, having more than 50% of their genome of Mesoamerican origin (EU_M), the proportion of those having low levels of admixture (< 20%) decreased to 79%. This lower value suggests that the EU_M individuals were more admixed than EU_A and that gene flow between pools probably played a key role during the introduction of *P. vulgaris* lines into Europe.

Geographically, both ancestral components were present across the sampled areas in Europe (Supplementary Figure 13). The Andean component (blue) was predominant in Portugal, Italy, Croatia and Bulgaria whereas the Mesoamerican one was present at higher frequencies around the Balkans, specifically in Albania, Serbia and Czech Republic. The accessions in the rest of the

localities showed a more balanced pattern characterized by the occurrence of different mixing proportions of the two ancestral components among individuals within each geographic area.

Increasing K to 3, the Mesoamerican gene pool divided into the AM_M1 and AM_M2 clusters colored in yellow and orange, respectively (Supplementary Figure 12), with approximately 50% of the individuals assigned to both genetic clusters. Although quite diffused in America, the occurrence of the orange cluster in Europe was very restricted, composing >25% of the single individual genome in only four samples (ECe105, ECe108, ECe122 and ECe094).

Supplementary Note 15. Genetic structure in America

Genetic structure in America was analyzed more in detail excluding all European accessions and repeating the ADMIXTURE runs from the beginning. The cross-validation plot indicated that the most likely number of genetic clusters given the observed data was three (Supplementary Figure 11). Focusing on the source of the introduction, the admixture analysis at K=2 confirmed the results obtained analyzing the whole sample (America and Europe; see Supplementary Note 14), highlighting the division between the Mesoamerican component (red) and the Andean one (blue), with nearly identical inferred admixture rates (Supplementary Figure 14) as the former analysis. The admixture analysis at K=3 including only American accessions (Supplementary Figure 14, Supplementary Figure 15) separated the AM_M1 (yellow) and the AM_M2 (orange) groups in Mesoamerica, from the third cluster composed by Andean individuals, hence supporting the persistence of three well differentiated groups in America. Admixed individuals containing both AM_M1 and AM_M2 genetic component were detected as well as the ones showing signals of introgression with the Andes.

The emergence of the two AM_M1 and AM_M2 groups in Mesoamerica was also confirmed by a separate analysis of individuals having a Mesoamerican genomic background, where the K=2 produced the lower cross validation error (Supplementary Figure 11).

The ancestry plot at K=2 (Supplementary Figure 16) in this restricted sample, was composed of pure AM_M1 (individuals completely yellow), pure AM_M2 (individuals completely orange) and admixed accessions (yellow and orange individuals). Pure AM_M1 accessions were located almost exclusively in Mexico (15 samples) and Chile (1 sample). Pure AM_M2 accessions were frequently

observed in Costa Rica, El Salvador, Honduras Nicaragua and less frequently in Mexico (Supplementary Figure 17). Pure AM_M2 accessions were also observed in South America, specifically in Chile, Colombia, Ecuador, Perú and Venezuela. Admixed individuals were present both in Mesoamerica and South America and were characterized by a wide spectrum of mixing proportions.

To better investigate the apparently absent genetic structure within the Andean genetic pool, an independent analysis including only individuals showing an Andean genomic background was performed. The inferred ancestry of the Andean ones resulted in four groups as indicated by the inferred cross validation error (see Supplementary Figure 11).

The ancestry plot at K=4 showed how most accessions were subdivided into three groups namely AM_A1 (pink), AM_A2 (purple) and AM_A3 (green) (Supplementary Figure 18). The genome of several accessions showed a complete membership to single clusters (e.g., ECa012 to the cluster AM_A1, ECa006 to the cluster AM_A2 and ECa003 to the cluster AM_A3) but admixed individuals were also present in the sample, especially as mixture between AM_A1 and AM_A2 components. The 12 pure AM_A1 individuals (pink) were found in Mexico (n=5), Brazil (n=2), Colombia (n=2), Costa Rica (n=1), Perù (n=1) and Argentina (n=1) (Supplementary Figure 19). The 14 accessions completely attributed to AM_A2 (purple) came from Argentina (n=7), Perù (n=3), Bolivia (n=2) and Ecuador (n=2). Lastly, the six accessions showing a complete membership to AM_A3 were found exclusively in Chile (n=6). The geographic localization of genetic components suggested a North to South pattern of genetic differentiation, with the AM_A1 and AM_A2 components be respectively more common in the Northern and Southern part of South America, and Chile being characterized by the AM_A3 genetic component. Admixed individuals between the AM_A1 and AM_A2 clusters were observed in Brazil (n=3), Colombia (n=1) but especially in Perú (n=9). Individuals composed by the other three combinations of genetic components (AM_A1/AM_A3, AM_A2/AM_A3 or AM_A1/AM_A2/AM_A3) were present but lacking a clear association with geography.

The ancestry plot at K=4 highlighted also a fourth component (red cluster) that, according to the whole-set ADMIXTURE analysis, was induced by the occurrence of Mesoamerican alleles having the AM_M1/AM_M2 components (Supplementary Figure 18 and Supplementary Figure 12). In fact, for ECa046, ECa011, ECa002 and ECa051 accessions, the previously estimated (America and Europe, k=2) proportion of Mesoamerican alleles was 19%, 16%, 6% and 7%. Despite the observed

co-occurrence of Mesoamerican alleles and the presence of the red component in these four individuals, two supplementary accessions (ECa017 and ECa022) showed a small part of their genome attributed to the Mesoamerican cluster (Supplementary Figure 18, Supplementary Figure 19) even though the 100% of their genome was attributed to the Andean pool by the K=2 ADMIXTURE analysis.

Supplementary Note 16. Population structure determined by chloroplast data

Twenty SNPs were identified at chloroplast level. Only the SNPs identified by both the two methods used (GATK and Samtools) were kept. Indels were removed. A minimum depth per sample of 3, a max-missing rate of 0.05 and a MAF > 0.05 were applied as filters. Population structure analysis was carried out with 19 SNPs; in particular, three SNPs, out of the total 20 SNPs identified, showed two different alleles for some individuals, thus we discarded the SNP "SNC_009259.1_75918", presenting a high percentage of individuals with two alleles (~21%), while for the others two SNPs we considered the data of individuals presenting two alleles as missing data (SNC_009259.1_35997, only one individual; SNC_009259.1_53504, eleven individuals). The Bayesian model-based approach implemented in the Bayesian Analysis of Population Structure (BAPS) software, version 5.3[29,30], was used to infer the population structure of the whole sample (218 *P. vulgaris* genotypes) and to assign the genotypes into genetically structured groups/populations (K). A mixture analysis was performed to determine the most probable number of populations (K) according to the data. The 'clustering with linked loci' analysis was chosen, to account for the linkage between sites within aligned sequences. Ten repetitions of the algorithm for each K ranging between 2 and 20 was applied. The best partition of our sample was set to K = 6 (CP1, CP2, CP3, CP4, CP5 and CP6) (Supplementary Figure 20). The relationships among the genotypes were investigated based on Neighbor-Joining (NJ) method by using MEGA X[31]. All positions containing gaps and missing data were eliminated (complete deletion option). The relative support for each node was tested using the bootstrap method with 1,000 replicates. The obtained unrooted NJ tree based on p-distance[32] is shown in Supplementary Figure 21 (condensed tree, cutoff value is 90%). The tree clearly separates (bootstrap value 99%) the CP1, CP2 and CP3 groups by the CP4, CP5 and CP6 groups. Based on this result and on comparison with the structure analysis performed with nuclear data the CP1, CP2 and CP3 groups are of Andean origin, while the CP4, CP5 and CP6 groups are of Mesoamerican origin (Supplementary Figure 20). Nuclear and chloroplast data are in agreement, with the exception of

some genotypes which based on chloroplast data belong to one of the two gene pools, while for nuclear data appeared to be introgressed from the other gene pool; almost all these genotypes were from Europe. The association between the results obtained by the BAPS analysis carried out with chloroplast data and the genetics groups defined by using nucleotide data by admixture and ChromoPainter analyses (see assignment procedure described in Supplementary Note 23) was tested by analysis of contingency tables with the likelihood ratio chi-squared (χ2) test, which was performed using the JMP 8.0 software (SAS Institute, Inc., 2008). Eight European accessions were excluded by this analysis not being assigned to a defined cluster (see Supplementary Note 23). The analysis of contingency tables indicated a significant association ($p < 0.0001$; likelihood ratio χ2 test; $R^2 = 0.42$) between the genetic groups obtained with chloroplast and nuclear data (Supplementary Figure 22). In particular, group CP1 is mainly represented by AM_A1, AM_A2 and EU_A1 groups, while CP3 includes mostly A3 accessions from both America (AM_A3) and Europe (EU_A3); the majority of CP2 and CP6 accessions are from Europe, with CP2 including EU_A1 and EU_A3 accessions, and CP6 being mainly represented by EU_M1 accessions. By contrast, the majority of CP4 and CP5 accessions are from America and belong to Mesoamerican gene pool; CP4 includes a higher proportion of AM_M1 compared to AM_M2 accessions, the opposite can be observed for CP5.

Supplementary Note 17. Spatial interpolation of membership coefficients in Europe and admixture levels in different domestication centres.

The interpolation of the Mesoamerican and Andean components inferred by ADMIXTURE at K=2 over the European map is reported in Supplementary Figure 23. The Mesoamerican component concentrated in three geographical areas: (i) in Western Europe, covering the Eastern part of the Iberian Peninsula including Northern and Central Spain, Northern Portugal and the North-Western part of France; (ii) in Central/East Europe, having a vertical distribution ranging from Eastern Germany/Poland to Albania and Greece, including a portion of Central Europe, specifically Austria, Czech Republic, Slovakia, Hungary and Central/South Italy; and marginally (iii) in the Black Sea area, comprising Turkey, Bulgaria and Ukraine, although the Mesoamerican component was represented by single accessions in each area and hence poorly supported by the data.

The Andean component was instead present in the rest of Europe, occupying (i) Western part of the Iberian Peninsula, including the majority of Portugal and Northwestern Spain; (ii) Central Europe, including Eastern France, Germany, Netherlands, Southern Sweden, Italy, Austria, Slovenia and

Croatia; (iii) Eastern Balkans, comprising Hungary, Romania, and Bulgaria; and iv) the Caucasian region. Here, we also analyzed *de-novo* the admixture in Chinese landraces (data from Wu *et al.*[33]), to compare the level of admixture between different centres of domestication (i.e., Europe and China) (Supplementary Figure 24). All variants reported in Wu *et al.*[33] (vcfs downloaded from https://zenodo.org/record/3236786#.Y49hDXbMK3A) for Chinese landraces (n=457) were filtered retaining biallelic sites with a minimum distance between adjacent SNPs of 250Kb and analysed using Admixture, fixing K=2. When considering a threshold of more than 5% of introgressed genome, we identified 125 (27%) Chinese admixed individuals, and 42 (37%) European admixed landraces, that suggests that admixture had been likely an important factor during adaptation in these regions. When we look for higher levels of admixture (>20%), in China, Mesoamerican and Andean gene pools shows similar level of admixture (7% of admixed accessions in each pool); using the same threshold of admixture, while in Europe, the Andean accessions are similarly admixed (11% of the accessions) to the Chinese, the 23% of the Mesoamerican accessions show admixture at higher levels (>20%). Based on this data, we can speculate that admixture, although with a different pattern among gene pools in the two centres, is relatively high in both regions.

Here, we also analyzed the admixture by Chinese provinces for which genomic data were available for at least ten landraces, and we calculated the average percentage for the Andean (Cluster 1) and Mesoamerican (Cluster 2) ancestries over these provinces (Supplementary Figure 24).
We observed that in regions located more in Northern Chinese provinces, that could overlap with the Silk Road path (for paths and cities crossed by the Silk Road, refer also to https://en.unesco.org/silkroad/silkroad-interactive-map; Supplementary Figure 24), the Mesoamerican ancestry seems to be prevalent, while following a North-South direction, the Andean ancestry is the more represented.

Although additional comparative studies are recommended among centres of domestication, these preliminary data are compatible with the scenario of an introduction of Mesoamerican genotypes from Europe to China following the Silk Road, followed by adaption processes of the two gene pools in China, leading also to different admixtures pattern than that observed in Europe. Moreover, this data is also compatible with an Andean ancestry introduction following sea routs from the South, and also exchanges of material with Japanese regions needs to be considered.

Supplementary Note 18. Networks

Phased SNPs contained in coding and neutral regions (see Supplementary Note 12-13 for details) were extracted from the whole callset and converted to a matrix of individual pairwise distance between accessions using the "—distance square0 1-ibs flat-missing" command in plink v1.9[34]. The distance matrix was transformed in the nexus format using the *phangorn*[35] *R* (R Core Team 2018) package, and SPLITSTREE v4.14.6[36] was used to obtain a phylogenetic network according to the Neighbor-net[37] algorithm. In order to test the robustness of the reconstructed networks to linkage disequilibrium (LD), the analysis was repeated over a random subset of 3,000 elements (roughly one every 150 kb), for each genomic partition.

Phylogenetic networks showing individual genomic similarities within America or between America and Europe were reported in Supplementary Figure 25 and Supplementary Figure 26. Single genomes were represented by circles colored by the ADMIXTURE attribution to gene pools (Mesoamerica and Andes) and their subgroups (M1, M2, A1, A2 and A3).

When only American accessions were included (Supplementary Figure 25), the reconstructed phylogenetic networks clearly supported the occurrence of two differentiated clades, one corresponding to the Mesoamerican gene pool and the other to the Andean one, using both the coding (Supplementary Figure 25A, B) and the neutral regions (Supplementary Figure 25C, D). An additional split within the Mesoamerican clade seems to be also present, separating the M1 subclade (yellow), composed mainly by accessions from Mexico (n=17), 2 samples from Chile and 1 form Argentina, and the M2 subclade (orange) composed by a more geographically heterogeneous set of accessions from Mexico, Central and South America. Three individuals (Eca_r2, Eca084 and Eca101), that were previously assigned to the M1 subgroup by ADMIXTURE, were located much closer to the M2 subclade indicating a greater affinity of these sample to the opponent subclade.

The Andean accessions appeared to be more homogeneous, with a less clear distinction between the A1, A2 and A3 subgroups than Mesoamerican counterparts, and shorter branch lengths (Supplementary Figure 25A). Most of A1 individuals clustered in a single clade composed by accession from Brazil (n=5), Mexico *(*n=5), Colombia (n=1) and Costa Rica (n=1), while 7 accessions were located outside the clade and, given the observed reticulations, suggested some level of admixture with other groups. Within A2 individuals, the phylogenetic network highlighted a subdivision between a subclade containing accessions from Argentina (n=8) and Bolivia *(*n=2), and

a second one composed by Peruvian samples (n=7). A3 individuals formed a more homogeneous clade including 7 samples from Chile and 1 from Colombia.

Based on their position on the network and the associated reticulations, several individuals showed evidence of admixture within and between gene pools. In particular, the topological position (e.g., halfway positions between pools in the network) of three individuals (Eca002, Eca011 and Eca046) on the coding network was in agreement with one or multiple admixture events between the Mesoamerican and Andean gene pools, whereas a supplementary individual from Chile (Eca017) showed the same signal according to the neutral network (Supplementary Figure 25).

Signals of admixture were also present in a group of accessions, attributed to A1 and A2 groups, from Peru (Eca021, Eca051, Eca022, Eca010, Eca048), Colombia (Eca028) and Ecuador (Eca008, Eca009), forming a separate clade, suggesting that some level of gene flow happened between the A1 and A2 clades in this geographic area.

Phylogenetic networks obtained controlling for linkage disequilibrium (LD) between regions produced topologies and reticulations that were very similar to the previous ones suggesting that LD did not affect the reconstruction (Supplementary Figure 25B,D).

The inclusion of European individuals did not alter the global structure of the previously reconstructed networks (Supplementary Figure 26), that was highly conditioned by the differences between the Mesoamerican (red) and the Andean (blue) gene pools. Most of the European accessions (black circles) clustered together with the American ones confirming that the Mesoamerica and the Andes were the main source of materials introduced into Europe and providing evidence about the geographic origin of the American accessions contributing to Europeans genomes. Within the Mesoamerican cluster (red), most of the European accessions belonging to it formed an external clade as expected in case of moderate admixture with the Andean gene pool. This European cluster was also observed in the other networks using different parts of the genome (Supplementary Figure 26B, C and D) including the Eca054, Eca073 and Eca102 American accessions, and occasionally Eca_r2. The same pattern was observed within the Andean cluster (Supplementary Figure 26, blue circles) where European accessions formed different clusters including a variable proportion of American accessions. However, the relationships between European accessions and Andean clades were not

stable, showing different topologies based on the genomic partition used to produce the phylogenetic network (e.g., Coding (A) vs Neutral (C) plot in Supplementary Figure 26).

Despite the observed diversity within clade, many samples were located outside the Mesoamerican and the Andean clades, close to the middle of the network, suggesting that intense events of admixture between the Mesoamerican and Andean gene pools were frequent during the introduction process into Europe.

Supplementary Note 19. Chromosome painting

The admixture analysis described above provided a global description of how each individual genome was partitioned among a prior defined number of ancestral populations. Since the recombination is not included in the model, the estimated admixture coefficients for each accession refers to the "whole amount" of genetic material inherited from the ancestral groups computed from single point positions along a genome (i.e., SNPs). The genomic segment between consecutive markers is therefore ignored and hence it is not possible to reconstruct the actual size of regions attributed to different ancestries. As a solution for this issue, the "chromosome painting" approach implemented in ChromoPainter v2.0[38] was applied to the phased variants. Under this model, recombination breakpoints are initially inferred for each individual and then used to define consecutive region of each chromosome ("chunks") that could be independently inherited through generations and hence that could possibly have different ancestries. After this stage, the ancestry of the haplotypes belonging to a set of accessions called "recipient" (i.e., samples that will be painted) are reconstructed as a series of chunks from a prior defined set of "donor" ones.

Following the recommendations in the manual, the phased SNPs contained in the vcf were converted using plink ("--recode12" option) and then to the proper format using the "plink2chromopainter.pl" and "chromopainter2chromopainterv2.pl" scripts. A uniform recombination map for each chromosome was prepared using the "makeuniformrecfile.pl". Prior to chromosome painting, the effective population size (Ne) and mutation rates (Mu) were estimated individually for each accession using 10 iterations of the Expectation-Maximization (EM) algorithm implemented in ChromoPainter. Each estimate was manually checked for convergence and eventually re-run using more iterations until stable estimates were obtained. After this optimization step, the estimated parameters were fixed in a new round of analysis producing the final chromosome painting of the "recipient" haplotypes.

Donor individuals were chosen according to their ancestry proportion inferred by admixture (Supplementary Data 4). The complete list of American accessions was filtered retaining i) Mesoamerican individuals showing a q value > 0.99 in the admixture run with K=3 using all American accessions, and ii) Andean individuals constantly having a q value > 0.99 from K=2 to K=4 in the admixture run restricted to Andean accessions. Following this procedure, 66 almost "pure" individuals were included in the donor list (36 from the Mesoamerican and 30 from the Andean gene pools), constituting the final panel from which the 114 European and the 38 American recipient individuals were painted. Donors were subdivided into the five groups inferred by ADMIXTURE (AM_M1, AM_M2, AM_A1, AM_A2 and AM_A3) and used to estimate their contribution to the ancestry of each SNP of the recipient individuals. Individual SNP probabilities were then combined in 10Kb not-overlapping sliding windows along chromosomes and subsequently, each window in each recipient haplotype was assigned to one of the five donor groups if a probability >=0.8 was observed. In the case in which the support of a single group was not over the threshold, the probability of the two Mesoamerican groups (AM_M1+AM_M2) and the three Andean ones (AM_A1+AM_A2+AM_A3) were combined and the same threshold (>=0.8) was used to attribute the window to a general Mesoamerican (MES) or Andean (AND) groups. If the combined probability for each gene pool was still not sufficient to reach the threshold, the window was marked as "unknown ancestry". After the assignment step of each 10Kb windows, the total proportion of genetic material coming from the seven groups or "unknown" was computed for each recipient individual and for each chromosome (both pairs). The final assignment of each recipient accession to the gene pools was done according to i) the total proportion of windows attributed to Mesoamerica or Andes, and ii) the number of chromosomes assigned to the two gene pools following the majority rule criterion (e.g., a chromosome was attributed to Mesoamerica if > 50% of windows came from such gene pool). The latter criterion was only used in the case in which the difference between the Mesoamerican and Andean inferred genome proportions was lower than 5%. In order to see where admixed segments were localized in the genome and how they were shared among individuals, one random haplotype for each chromosome from each accession was extracted and the assignment of each 10Kb windows to the different genetic pools was plotted according to their genomic coordinates.

The attribution of each genomic window to the seven groups was also used to estimate the length of the introgressed blocks within each European accession. Each haplotype of the EU_AND accessions (according to the global ChromoPainter attribution, n=71) was traversed merging consecutive windows, attributed to any of the Mesoamerican clusters (AM_M1, AM_M2 or MES), forming larger

blocks. Bedtools[39] *merge* command was used to join windows within a maximum distance between elements of 50Kb in order to deal with occasional window misattributions that could artificially break introgressed blocks. The length of each Mesoamerican block in each EU_AND individual was recorded for each chromosome and was then filtered removing blocks composed by single windows (10kb). The final within-individual distribution of lengths was characterized by the median, due to its non-normality.

The chromosome painting of the American accessions allowed us to attribute each of the 38 accessions to the corresponding genetic pool: 21 of them had an Andean origin whereas the remaining 17 ones were attributed to the Mesoamerican pool (see Source Data for Figure 3a). As expected, each of them showed marked signal of introgression from the other gene pool, with proportions from 3.5% to 44.8% for the Mesoamerican accessions and from 8.7% to 41.8% for the Andean ones.

Among European accession, 71 were attributed to the Andean gene pool (EU_AND) and 43 to the Mesoamerican one (EU_MES) (see Source Data for Figure 3a). Compared to the admixture attribution at K=2, there was a strong correlation (r=0.99) between the ancestry proportions inferred by the two methods (Supplementary Figure 27) both for the Andean and the Mesoamerican component. Despite the overall agreement, an average difference of 2.7% and 3.8% in the estimated genomic attributions between methods were present across the analyzed American and European accessions. Such inconsistency was particularly evident for 5 European accessions (ECe027, ECe077, ECe080, ECe093, ECe153, ECe179) where the Admixture attribution to the AND or MES was 99% whereas ChromoPainter supported a more heterogeneous composition from the two genetic pools, with deviations from 15% to 19% of the estimated proportions.

Globally, the inferred amount of per-accession introgressed material was different between EU_MES and EU_AND accessions (two-sided K-S test, p=3.3x10^{-3}), showing a median proportion of 4.7% and 9.2%, respectively. EU_MES accessions had from 0.1% to 44.9% of their genome introgressed from the other genetic pool (Figure 3a). These proportions were similar in the EU_AND samples, ranging from 0% to 42.3%. The pervasive effect of admixture in European individuals was confirmed by the presence of several accession showing more than 20% of their genome composed by introgressed material, in both EU_MES (8 accessions, n=43, 18,6%) and EU_AND (11 accessions, n=71, 15,5%) groups.

The between-pools introgressed genomic chunks (i.e., Mesoamerican haplotypes present in EU_AND accession and vice versa) had a sparse distribution over the genome of European individuals, composed both of short and very long blocks approaching the chromosome size in some cases (e.g., WGS14 individual, Chr01, Supplementary Figures 28-38). The median length of the introgressed genomic segments was higher for EU_AND than EU_MES accessions (And: 217 Kb, Mes: 70 Kb, Mann-Whitney test, p=7.22x10^{-10}); EU_AND carried longer Mesoamerican haplotypes (Figure 3b). This observation was not consistent across chromosomes (Figure 3c, Supplementary Table 7). Specifically, Chr02, Chr05, Chr06 had a median per-individual introgressed chunk lengths in EU_AND that was much higher than in EU_MES, with a median of 503, 160, and 165 Kb in EU_AND *versus* 38, 50 and 50 Kb in EU_MES. The opposite pattern was observed for Chr03, Chr04 where the median was higher in EU_MES accessions having a median of 110 and 153 Kb in EU_MES and 40 and 90 Kb in EU_AND. The median length was approximately the same in the rest of the chromosomes. The maximum length of the introgressed genomic blocks measured in the EU_AND chromosomes was always higher than what present in EU_MES individuals (Supplementary Table 7), suggesting that EU_AND accessions retained very long Mesoamerican haplotypes in their genome. To evaluate the impact of the potential mis-assignment of some individuals on the results, the identification of the introgressed blocks was repeated excluding three EU_MES (ECe112, ECe895 and ECe901) and three EU_AND (ECe033, ECe035 and ECe109) accessions showing an admixture proportion >=40% (as estimated by Admixture or Chromopainter), producing consistent results (Supplementary Figure 39).

Screening the plots in Supplementary Figures 28-38, haplotypes with a specific ancestry seem to be shared across almost the entire set of EU accessions (e.g., position 46Mb on Chr01), suggesting that these regions could be involved in the process of adaptation of *P. vulgaris* lines in the European continent.

Supplementary Note 20. Admixture time estimation

The length of the introgressed blocks (see also Supplementary Note 19) characterizing European individuals was used to estimate the time since admixture using the model proposed in Janzen & Pina[40] for haploid chromosomes. A single haplotype was extracted from each European accession among those having >=10% of their genome introgressed from the other genetic pool (EU_AND, n=35 and EU_MES, n=10). The whole set of alleles along chromosomes was filtered removing centromeric/pericentromeric regions and retaining 50,000 alleles having a minimum distance of 1000

bp between each other. Each individual was independently analysed using the "estimate_time_haploid" function in the "junctions" R package setting the effective population size (Ne) to 500 and the per-chromosome recombination rate of Schmutz *et al.*[13]. In each analysis, chromosomes having <5% of admixture were removed, and time estimates close to the upper bound (1000 generations), hence indicating potential inferential issues, were discarded (6 EU_AND and 3 EU_MES accessions).

The time since admixture estimated for EU_AND (n=29) and EU_MES (n=7) accessions is reported in Supplementary Figure 40. We can infer that the EU_AND gene pool has been introgressed more recently than the Mesoamerican gene pool (Mann-Whitney p=0.0008). Considering a generation time of one year, the median estimated admixture time was 98 and 155 years in EU_AND and EU_MES accessions, respectively. To consider possible effects induced by the effective population size and recombination rate choice, the whole estimation procedure was repeated changing the Ne values (250 and 1000) and the recombination rate as estimated by Blair *et al.*[41], obtaining consistent results.

Supplementary Note 21. Tagging the signatures of adaptation in Europe: Genome-wide selection scan

Overall, 180 accessions assigned to the Andean and Mesoamerican gene pools in America and Europe (66 donor American accessions; AM_M, AM_A, and 114 EU_A and EU_M accessions) were used to detect signature of selection along the genome. European accessions, derived from American gene pools, probably underwent a combination of demographic events related to their introduction in a new habitat and the effect of natural selection promoting local adaptations, that shaped the current pattern of genomic variation in the European continent. To pinpoint genomic regions showing the signature of positive selection, the hapFLK method[42] was used. Under this method, the haplotype differentiation and the linkage disequilibrium pattern along chromosomes were inferred from genomic polymorphism and used for the detection of selection signatures, accounting for the hierarchical structure of populations. The local genomic differentiation along chromosomes, as measured by haplotypic F_{ST}, was compared to the expectation given by the inferred genomic relationships between groups (whole genome population tree), taking into account the genetic drift within groups. The ability of such inferential method to deal with structured populations made it particularly suitable for the analysis of how structured source (America) and sink (Europe) groups diverged and possibly developed specific adaptations to new environments.

Accessions were subdivided in the AM_A (n=30), AM_M (n=36), EU_A (n=71) and EU_M (n=43) groups and VCFtools was used to sample a single SNP every 250kb (--thin 250000). This set of SNPs was used to estimate a neighbor joining tree and a kinship matrix according to the Reynolds' genetic distance matrix between the four groups of accessions, constituting a genome wide estimate of population structure. The hapFLK statistics was then computed on each chromosome independently over the complete SNP dataset and averaged over 20 expectation maximization cycles to fit the LD model. A first analysis was performed fixing the number of haplotype clusters to 5, as suggested by the admixture analysis. A second run was conducted selecting the appropriate number of haplotype clusters based on the fastPHASE[43] cross-validation procedure, implemented in the *imputeqc* R package (https://github.com/inzilico/imputeqc). Under this approach, VCFtools was used to extract a subset of SNPs spaced at least 100kb (--thin 100000 option) for each chromosome and the "make_test_files.R" script was called to generate five independent copies of such SNP set, randomly masking the 10% of the variants. The fastPHASE v1.4.8 software was run on each dataset imputing the missing genotypes, setting the number of haplotype clusters K to 5, 10, 20, 30, 40 and 50. The *EstimateQuality function* was used to compute the proportion of wrongly imputed genotypes (Wp) for each combination, and the K value, minimizing the mean Wp proportion across the five SNP set replicates, was selected as the most supported number of haplotype clusters. The analysis was replicated using all (AM+EU, n=180) or only American accessions (AM, n=66).

The "scaling_chi2_hapflk.py" script was used to scale hapFLK values and compute the corresponding *p*-values. The significant SNPs, showing a pvalue < 10^{-3} (fdr < 0.05), were extracted and bedtools was used to create a region of 10kb centered on each significant SNP and to merge overlapping regions within a maximum distance of 5Kb. The two set of regions, obtained fixing K=5 or using the fastPHASE estimated K value in two independent hapFLK runs, were merged together forming the extended set, constituted by the union of the two sets, and the restricted set, containing only regions supported by both runs. To pinpoint putative regions under selection in Europe, the "Extended" and the "Restricted" set of regions were intersected with the F_{ST} windowed analysis, and only regions containing at least one F_{ST} window located in the top 5% or top 1% were retained (see also Supplementary Note 30).

The estimated number of haplotype clusters in the whole dataset (AM+EU) was 20 for all chromosomes with the exception of Chr05 and Chr09 supporting 30 clusters and Chr06 where only 10 clusters were estimated (Supplementary Table 8). Restricting the analysis to 66 American

accessions produced qualitatively the same results with many chromosomes supporting 10 or 20 haplotype clusters.

The "Extended" set was composed by 150 regions, 98 of them being highly differentiated between America and Europe at 5% F_{ST} level and 48 regions at the higher top 1% F_{ST} threshold (Supplementary Data 5).

The "Restricted" set included 61 regions, 51 showing levels of differentiation located in the top 5% F_{ST} values and 26 regions in the top 1%.

Supplementary Note 22. Spatial distribution of genetic groups in Europe and environmental association analysis

The spatial interpolation of the Mesoamerican (M1, M2) and the Andean (A1, A2, A3) ancestry components in Europe, as inferred by ChromoPainter analysis (Figure 1e in the main text), highlighted a diverse distribution in the European regions. Starting from this distribution, spatial analyses were performed to investigate if the genetic variation of the European accessions was associated to their geographical distribution or the ecological patterns. The ecological data (about 1-km^2 resolution) were downloaded from WorldClim data (http://www.worldclim.org, Hijmans *et al.*[44]) for a total of 19 bioclimatic variables and 24 monthly variables (precipitations and min, mean and maximum temperatures from April to September) as reported in Supplementary Data 6. At first, a Mantel test has been performed to verify if any significant associations between the genetic and geographical distances were present among all the European accessions, or, if any autocorrelation was detectable among different genetic distance classes. The genetic distances among accessions were calculated using all the callable SNPs. The vegan R package[45] was used to calculate the geographical distances, the ecological distances, the Mantel statistics, and the spatial autocorrelation. The Mantel statistics was tested by 10^3 permutations and the autocorrelogram was calculated among 10 distance classes of nearly 540 km each, calculating the significance of the correlation per each class by 9999 permutations.

No significant correlation was found between the genetic and geographical distances (r = -0.05, p-value = 0.921) and the autocorrelogram was marginally significant only for the second distance class (r = -0.04, p-value$_{corrected}$ = 0.042), thus indicating the presence of random spatial patterns among the

European accessions. As a second step, we have performed a Mantel test to verify if an association was detectable between genetic distances and ecological distances. The ecological distances were calculated from the 19 bioclimatic variables.

Results did not evidence any association between the genetic distances and the ecological distances (r = -0.01, p-value = 0.627). As a further step, an environmental association analysis was then performed to identify if any climatic factors have led to an adaptive genetic variation in Europe for the different genetic groups. To perform these analyses, a multivariate correlation analysis was performed between the Pvalues (proportion of the genetic membership to the five genetic groups M1, M2, A1, A2 and A3) assigned to each European accession and the ecological variables registered at the collection site. This analysis has revealed a significant negative association between the P(AM_A3) values and the latitude (Supplementary Table 9 and Supplementary Data 6) indicating that the accessions classified as Eu_A3 are usually located at lower latitudes in respect to other subgroups.

Significant associations have been also detected between the membership Pvalue and different climatic variables (Supplementary Table 9). Among these, the ones detected for the P(AM_M1) indicate that accessions mainly attributed to the subgroup Eu_M1 seem to be preferentially distributed along those areas where higher mean diurnal ranges in temperatures (coded as BIO2), higher temperature seasonality (temperature changes over the year, coded as BIO4) and higher temperature annual ranges (coded ad BIO7) may occur. Similarly, significant positive associations where also observed between the P(AM_M2) values of the European accessions and the BIO4 and BIO7 variables.

On the other hand, significant negative associations were detected between the P(AM_A1) values and the BIO2 and BIO5 and Tmax of July and August, suggesting that the accession belonging the Eu_A1 subgroup are preferentially distributed along areas where lower mean diurnal ranges of temperature and lower temperatures in the warmest months are registered (Supplementary Table 9). Regarding the accessions mainly attributed to subgroup Eu_A3, a preferential distribution in warm climatic areas seems to emerge (Supplementary Table 9).

Supplementary Note 23. Genetic structure, molecular phenotyping and flowering data

Here, we investigated the association between flowering time (See Supplementary Note 6) and the genetic structure on 199 common bean accessions (BEAN ADAPT Pv_core2) for which phenotypic data on flowering time are available. In more detail, the Analysis of Variance (ANOVA) was performed between the subgroups that were identified within the two genetic pools (i.e., Mesoamerican and Andean) both in America and Europe. Here, the first principal component (PC1_FLW), which describes the 68.8% of the phenotypic variance for DTF and for the photoperiod sensitivity (see Supplementary Figure 4), was used as a representative phenotypic trait in order to perform the ANOVA between the genetic subgroups. We previously defined a set of American donor accessions that were assigned to different genetic groups [i.e., P(AM_A1), P(AM_A2), P(AM_A3), P(AM_M1), and P(AM_M2)] (See Supplementary Note 15, 19). Moreover, the percentage of membership to different genetic subgroups was established also for the so-called "recipient" accession with the ChromoPainter analysis (See Supplementary Note 19). With the aim to perform the ANOVA for FLW across well-defined subgroups, each American and European recipient accession has been assigned to the subgroup which showed the relative majority membership compared to the other subgroups (i.e., AM_A1, AM_A2, AM_A3, AM_M1, AM_M2, EU_A1, EU_A3, EU_M1, EU_M2 and EU_MIX). We classified as admixed those accessions showing a PMeso value comprised between 0.4 and 0.6. According to the ANOVA analysis, significant differences were observed for FLW between genetic subgroups (F ratio 59.63; *p*-value <.0001). The results of the Tukey-Kramer test for each pair of comparisons between subgroups are reported in Supplementary Table 10. Interestingly, any of the European accessions were classified as mainly belonging to the subgroup A2. Moreover, this genetic subgroup (i.e., A2) was poorly represented in the European accessions that were assigned to other Andean subgroups (i.e., 0.065 in EU_A1 and 0.060 in EU_A3) (Supplementary Figure 41). Similarly, also the M1 genetic subgroup was relatively low represented in Europe (0.398 in EU_M1 and 0.184 in EU_M2) and it is more admixed compared to the EU_M2 group, which instead is composed of more than 50% for the genetic cluster to which it belongs (i.e., AM_M2). Overall, these results indicate that the two genetic clusters AM_A2 and AM_M1, which are mostly represented in the American accessions (i.e., 0.836 in AM_A2 and 0.843 in AM_M1) showing high sensitivity to the photoperiod and late flowering, are less represented in Europe than the other clusters (Supplementary Figure 41). Plants can cope with persistent fluctuations in agro-ecological conditions through adaptive mechanisms; thus, when *P. vulgaris* was introduced in Europe, favorable combinations of alleles that ensured a reduced sensitivity to the photoperiod

have been selected and fixed. Contrarily, it can be postulated that the genetic subgroups AM_A2 and AM_M1, which are sensitive to the photoperiod, experienced a selection pressure after their introduction in Europe. This resulted in a reduction in their contribution to the genome of the modern European accessions that carry favorable allele combinations at the basis of their adaptation. Moreover, the individuals from the Chile race (genetic group AM_A3) did not show a significant delayed flowering when considered as a unique group, in comparison with the individuals from the Race Nueva Granada (genetic group AM_A1) (Supplementary Table 10). However, a few individuals from Chile race (AM_A3) have a slightly delayed flowering (Supplementary Figure 41) in American accessions grown in Europe, and in particular they show delayed flowering at certain latitudes (Figure 2e in the main text). When we compared the flowering time of the genetic groups from Europe, by removing the highly sensitive genetic groups from the Americas, the individuals mainly belonging to the race Chile (EU_A3) showed a delayed flowering that is significant when compared to the individuals that are mainly assigned to the genetic group A1 (race Nueva Granada) (Supplementary Table 10, Supplementary Figure 41). According to Wallace *et al.*[46] a significant admixture between gene pools has been reported in Europe and North America for the BeanCAP snap bean panel. When we considered separately dry and snap bean type, according to our passport data (Supplementary Data 1), five American and 16 European common bean accessions result to be of the snap bean type, with only three from Europe showing an admixture higher than 20%, We also compared flowering data of snap beans and dry beans from Americas and Europe and we observed that the dry beans from Americas show delayed flowering in Europe compared to the remaining three groups (i.e., dry beans from Europe, and snap beans from Europe and from Americas), among which we did not observe significant differences for the flowering time (Tukey-Kramer HSD) (Supplementary Figure 41).

Following the same approach, we performed an ANOVA between the genetic subgroups with the aim to find an association between the genetic structure and the molecular phenotype (See Supplementary Note 5). In detail, the PC1, that was obtained from the secondary metabolites having a high hereditability (H_{2}>0.65) (non-targeted secondary metabolite analysis; see Supplementary Note 5), was used as a phenotype for comparison between the genetic subgroups (Figure 2c). According to the ANOVA analysis, significant differences were observed for the molecular phenotype (i.e., secondary metabolites) and the genetic structure (F ratio 185.35; *p*-value <.0001). The results of the Tukey-Kramer test for each pair of comparisons between subgroups are reported in Supplementary Table 11. With regard to the Andean gene pool, no significant phenotypic differences were detected between continents (i.e., between American and European subgroups). On the other hand, concerning

the Mesoamerican gene pool, the European subgroup EU_M1 was phenotypically different from the Mesoamerican American ones, and from the AM_M2 subgroup in particular, while the European subgroup EU_M2 was phenotypically different from the AM_M2. This might be the result of the high-level of Andean introgressions into the Mesoamerican gene pool in European accession, which, according to our data, have occurred with a higher magnitude than the opposite scenario (i.e., Mesoamerican introgression in the Andean gene pool) (See also Supplementary Note 31). We also analyzed the distribution of the Q_{ST} for the same highly inheritable putative secondary metabolites and for the flowering data, performing the ratio between the genetic variance attributable to differences among Continents (i.e., American *vs* European accessions) over the entire genetic variance for each trait. Here, we observed that the Q_{ST} value for the flowering (0.33) is on the 97.5% percentiles of the distribution for the Q_{ST} for the highly hereditable metabolites, and in the top 99.5% of our F_{ST} distribution genome-wide. The comparison of the Q_{ST} values among molecular phenotypes and flowering data, in parallel with the clear pattern of flowering time as reported in Supplementary Figure 41 supports that flowering is a candidate trait that underwent a selection process associated to the introduction in Europe[47,48].

Supplementary Note 24. Genetic diversity: Baseline levels of genetic diversity

The theta estimator based on the mean number of pairwise difference (θ_π[49]) was used to quantify the levels of genetic diversity within groups of accessions defined according to their geographic origin and gene pool (see also Supplementary Note 25, Supplementary Table 12 and Supplementary Table 13). The "--site-pi" VCFtools flag was used to obtain a per-SNP estimate that was subsequently filtered, according to the genome annotation, including only positions located:

a) in callable regions, defined as genomic positions that did not show an overlap with any repeated region (276 Mbp);
b) in coding regions, defined as exonic regions corresponding to the longest isoform for each gene (34 Mbp);
c) in neutral regions, defined as callable regions where all the genes and the 10 kb flanking regions were removed (44 Mbp).

The per-site θ_π estimate was then summed up and divided by the size of each specific region (callable, coding or neutral) in order to have a global estimate over it. A raw estimate (i.e., not taking into

account the annotation) of θ_π along chromosomes, averaged over 100kb not overlapping windows, was also computed to highlight chromosomal regions having different levels of genetic diversity.
The observed levels of within-group genetic diversity for each genomic partition are reported in Supplementary Table 12, Supplementary Figure 42 and Figure 4a. America and Europe showed similar diversity levels of 18.7x10-4 and 15.9x10-4 θ_π/bp in callable regions, respectively. Approximately, the same relative level of diversity between continents was observed in the coding (11.2x10-4 in America vs 9.6x10-4 in Europe) and neutral (9.2x10-4 in America vs 8.0x10-4 in Europe) part of the genome, with a slightly high level of diversity in America.

Within America, a high level of diversity was observed in Mesoamerica (AM_M, 10.7x10-4) compared to the Andes (AM_A, 3.1x10-4) in callable regions, showing a reduction of diversity of approximately 3.45 times in the latter group. A consistent decrease in genetic diversity of the same magnitude was observed in the coding and the neutral genomic partitions.

Within Europe, the observed diversity in callable regions of the EU_M group was 1.6 times higher than EU_A one (11.3x10-4 in EU_M vs 7.1x10-4 in EU_A), and it was similar to what observed in the AM_M group from which it was probably introduced into Europe. On the other hand, the comparison of the Andean individuals in Europe (EU_A) and America (AM_A) highlighted a higher diversity in Europe than in America, with the former having a θ_π/bp roughly 2.3 times higher than what observed in the American counterpart.

The relative observed pattern of diversity in the different groups of accessions was also coherently supported by coding and neutral regions. Interestingly, the coding regions appeared to be more polymorphic than neutral regions in all comparisons suggesting that the process of introduction of *P. vulgaris* in the Andes and in Europe affected differently the coding and the non-coding regions.
The θ_π variation along chromosomes was not uniform, highlighting part of the chromosomes having a reduced polymorphism level, correlating with the position of centromeric and pericentromeric regions, and other ones that conserved more genetic variability (Supplementary Figures 43-45).

Supplementary Note 25. Levels of genetic diversity after admixture masking

The identification of a not-negligible amount of introgressed genomic regions in admixed European individuals described in Supplementary Figure 12 suggested that the previously estimated levels of genetic diversity could be biased (probably overestimated) in Europe (EU_M, EU_A and EUROPE

groups) (see Supplementary Note 24). To take this fact into account, all variants in the vcf file were filtered masking all alleles identified to be introgressed by the ChromoPainter analysis or with an ambiguous assignment, within European accessions. The "--site-pi" and "--missing-site" commands in VCFtools were used to obtain a per-site θ_π estimate and the proportion of missing data (masked individuals) for each position, respectively. Plots of θ_π along chromosomes were generated following the same procedure applied for the unmasked dataset. The global within group estimate of θ_π was computed for the callable, the coding and the neutral genomic partitions, excluding regions with an average (over SNP) minimum mean proportion of not-masked individuals (PIND) from 0 to 100%. In this way, the stability of the θ_π estimate at different missing data levels was evaluated.

The θ_π estimates were robust to the PIND thresholds chosen, showing very small differences in the interval from 0 to 0.7 and starting to deviate from the baseline values for higher PIND levels (Supplementary Figure 46). This result was consistent across groups of accessions (EU_A, EU_M and EUROPE) and genomic partitions (callable, coding and neutral).

Overall Europe, the admixture masking procedure did not substantially change the observed level of diversity where we got a θ_π estimate of 15.5x10-4 versus the 15.9x10-4 of the previous one, in the callable regions (Supplementary Table 12 and Supplementary Table 13, Supplementary Figure 47, Figure 4b). This shallow decrease was also observed in the coding (9.3x10-4 vs 9.6x10-4) and in the neutral (7.8x10-4 vs 8.0x10-4) part of the genome. However, the admixture masking step produced a sharp decrease in genetic diversity within Europe when considering the two gene pools. The level of genetic diversity in the European individuals with Mesoamerican genomic background (EU_M) was 1.68 times lower than unmasked one in callable regions (6.7x10-4 vs 11.3x10-4) whereas the loss of genetic diversity was slightly less evident in coding (5.0x10-4 vs 7.5x10-4) and neutral regions (4.0x10-4 vs 5.9x10-4) showing a decrease of 1.5 times. This reduction in genetic diversity was particularly marked in European accessions having an Andean genomic background (EU_A), where the estimated diversity was 3.55, 2.61 and 3 times lower than before the admixture masking process, in the callable (2.0x10-4 vs 7.1x10-4), coding (1.8x10-4 vs 4.7x10-4) and neutral (1.3x10-4 vs 3.9x10-4) genomic regions.

The comparison of the new estimated levels of genetic variation within Europe with the American pools indicated a clear reduction in diversity that was not observed in the unmasked data. Specifically, the θ_π estimate in EU_M group showed a 37% reduction in diversity compared to AM_M group in

callable regions. This reduction was similar in coding (33%) and neutral (29%) regions. A reduction in diversity was also observed in the EU_A group showing a lower θ_π estimate than the AM_A counterpart in callable regions. The inferred reduction was 35%, 31% and 24% in callable, coding and neutral genome partitions. A small reduction in diversity was also observed between America and Europe, with the latter showing 17%, 17% and 15% less genetic variation in callable, coding and neutral regions than the former.

To evaluate the impact of the potential mis-assignment of some individuals on the results, the genetic diversity was also re-estimated excluding three EU_MES (ECe112, ECe895 and ECe901) and three EU_AND (ECe033, ECe035 and ECe109) accessions showing an admixture proportion >=40% (as estimated by Admixture or Chromopainter), showing no substantial differences.
The $\theta\pi$ variation along chromosomes is showed in Supplementary Figure 48-58.

Supplementary Note 26. Loss of diversity

In order to test whether the introduction of American lines into Europe affected the observed levels of genetic diversity, the θ_π estimate was compared between the two putative American sources of genetic variation (*i.e.*, AM_M and AM_A) and the two European groups (i.e., EU_M and EU_A). A sampling without replacement of 30 individuals for each group was performed (except for AM_A where n=30) to exclude the effect of uneven sample size in computations. For each European individual, the same procedure explained in the Supplementary Note 25 (Levels of genetic diversity after admixture masking**)** was applied to mask all alleles belonging to introgressed regions or showing uncertain attribution to the gene pools. All alleles located in genomic segments identified as under selection in the Supplementary Note 31 (the “Extended” set of regions) were also excluded. The per-site θ_π estimator was computed allowing the 70% of maximum proportion of missing genotypes and was averaged over 100kb not overlapping sliding windows. The “Loss of diversity” score (LoD, Vigouroux *et al.*[50]) was computed as 1-(mean ($\theta_{\pi\text{-EU}}$)/mean($\theta_{\pi\text{-AM}}$)), where $\theta_{\pi\text{-AM}}$ and $\theta_{\pi\text{-EU}}$ were the mean estimated diversities across 100kb windows in American and European groups, respectively. One hundred sampling replicates were performed. The analysis was also repeated computing the LoD score in the regions under selection, in order to see whether the introduction process into Europe impacted differently on different regions of the genome. Only regions under selection being also in the 5% top F_{ST} between Europe and America were considered.

Across replicates, Mesoamerican lines showed a median LoD score of 0.36 (min 0.31, max 0.42) that was significantly higher (Wilcoxon test, *p*=2.2x10-16) than the Andean one (LoD 0.33, min 0.27, max 0.38) (Supplementary Figure 59), suggesting that the reduction of genetic diversity due to the introduction process into Europe was stronger for the Mesoamerican gene pool, over the genome. Compared to the genomic baseline level, the LoD in regions under selection was 0.32 (min 0.15, max 0.51) and 0.39 (min 0.28, max 0.49) for the Mesoamerican and Andean gene pools. The LoD in regions under selection was higher than the baseline level for the Andean gene pool, indicating a further reduction of diversity in Europe probably due to the effect of positive selection as main driver. In the Mesoamerican gene pool, the median LoD was lower than the Andean one in regions under selection (Supplementary Figure 59). However, replicates showing the highest and the lowest loss of genetic diversity were present in this set indicating the coexistence of individuals with a marked reduction in diversity and others showing more genetic variation than expected. This latter case could be the product of balancing selection or the introgression of genetic material from the wild genetic pool.

Supplementary Note 27. Genome-wide Linkage Disequilibrium (LD): Linkage Disequilibrium decay

The relationship between linkage disequilibrium and physical distance along chromosomes was evaluated in America and Europe, and successively within the AM_M, AM_A, EU_M and EU_A groups. The *PopLDdecay*[51] tool was used to compute r^2 correlation between allele frequencies at pairs of SNPs along the chromosomes, setting a minimum minor allele frequency of 0.1 and a maximum distance between SNPs of 5 Mbp. The mean estimated r^2 values were plotted against the physical distance between SNPs to trace the overall linkage disequilibrium decay over chromosomes.

The American and European groups showed a decay in LD increasing the physical distance between markers. In America, the r^2 decreased very rapidly reaching the value of 0.4 approximately at 100Kb and then staying stable also at higher physical distances. Compared to America, the European accessions showed a slower LD decay, approaching the r^2 of 0.4 at 2Mb distance between markers. The analysis of Mesoamerican and Andean accessions revealed the same pattern of decay, where the LD decreased more rapidly in America than in Europe (Supplementary Figure 60). The AM_M group had an r^2 of 0.15 at 500Kb whereas the same LD was observed at 2Mb in the EU_M. The AM_A group reached an r^2 of 0.2 at 500kb but this value was never reached by the EU_A group neither at 5Mb.

The Andean accessions were also characterized by a higher baseline level of LD. An r^2 of 0.2 was indeed reached at 500kb for AM_M and 1Mb for AM_A, while an r^2 of 0.3 was reached at 250Kb for EU_M and 1.5Mb for EU_A.

Supplementary Note 28. Synonymous and missense mutations

Polymorphic positions in the vcf file were parsed according to the SnpEff annotation. All variants that were classified as "missense_variant" or "missense_variant&splice_region_variant" contributed to the missense set, whereas variants having the "synonymous_variant" or the "splice_region_variant&synonymous_variant" tag constituted the synonymous set of SNPs. The total number of stop codons that were gained ("stop_gained" and "stop_gained&splice_region_variant" tags) or lost ("stop_lost" and "stop_lost&splice_region_variant") was also considered forming the loss-of-function (LoF) set. All the variant positions, referring to the four different types of mutations, were intersected with the genomic coordinates of the longest isoform for each gene, in order to avoid redundancy. Results are reported in Supplementary Data 7. Finally, for each individual we recorded the total number of non-reference alleles at each retained SNP position for each class of mutations, counting one or two non-reference alleles for each heterozygous or homozygous genotype, respectively.

The observed ratio of missense alleles over synonymous alleles within the Mesoamerican and Andean genetic pools was similar between America and Europe. The Mesoamerican groups were characterized by a median ratio of 0.78 that was shared between the two American groups (AM_M1 and AM_M2) and the European one (Figure 4d). The same ratio was observed also in the admixed group of American accessions belonging to the Mesoamerican gene pool (AM_MES). The ratio in the Andean groups was higher than what observed in Mesoamerican accessions and more heterogeneous between groups. The highest median ratio was observed in the AM_A3 accession having 1.02 missense/synonymous alleles. A lower value of 0.99 and 0.97 was observed in the AM_A1 and AM_A2 accessions, respectively. A ratio of 0.97 was also observed in the admixed accession belonging to the Andean gene pool (AM_AND). The lowest ratio was observed in the EU_A group, showing a median proportion of 0.92 missense/synonymous alleles, ranging from a minimum of 0.84 to a maximum of 1.00, among accessions.

A similar pattern was observed in the median ratio of LoF over synonymous alleles, with all Mesoamerican groups showing a lower ratio compared to Andean groups (Figure 4d), going from 8.64x10-3 to 8.92x10-3 of AM_M1 and AM_M2 group respectively. A proportion of 11.88x10-3, 12.19x10-3, 12.88x10-3 and 12.05x10-3 was observed in AM_A1, AM_A2, AM_A3 and AND groups, belonging to the Andean genetic pool. Among Andean groups, the lower median value was observed in the European one (EU_A) with 11.31x10-3 LoF over synonymous alleles, with some accessions showing median values comparable to the Mesoamerican genetic pool (ECe145: 7.8x10-3; ECe033: 9.28x10-3).

Supplementary Note 29. Pattern of private alleles

Missense and synonymous variants were screened in American (n=66) and European (n=114) accessions following the same steps described in the previous section. Variants that were private of the European or the American group were retained and divided in low (below 5%) and medium-high (above 5%) within-sample frequency. The genomic coordinates related to private alleles segregating at different frequencies in the American and European groups of accessions were intersected with the gene annotation, and the burden of missense and synonymous mutations was recorded for each gene element.

The ratio of missense over synonymous alleles differed among continents and among frequency classes (Supplementary Figure 61). In Europe, the ratio at alleles segregating at low frequency (EU<5%) was 1.44. This value was higher than the ones segregating at frequencies >5% in the same continent (EU>5%, 1.19) but also higher than what observed in America regardless of the frequency class. In America, private mutations segregating at low frequency (AM<5%) were also enriched of missense alleles, showing a proportion of 1.2 that was higher than what observed at higher frequencies (AM>5%, 0.85).

Supplementary Note 30. Divergence estimates (F_{ST})

The magnitude of the genomic differentiation between and within America and Europe was evaluated using the Weir & Cockerham estimator of F_{ST} (Weir & Cockerham[52]). The baseline differentiation between the two continents was initially estimated pooling together the AM_M and AM_A accessions and the EU_M and EU_A accessions forming the American and the European groups, respectively.

The amount of differentiation within America and Europe was evaluated comparing EU_M vs EU_A and AM_M vs AM_A, respectively. The differentiation between continents was measured comparing AM_M vs EU_M and AM_A vs EU_A.

The pairwise weighted F_{ST} was then computed in 10kb not-overlapping sliding windows between each pair of groups using VCFtools. The mean and the interquartile range (IQR) of the windows-based distribution were used as point estimate of the differentiation between groups and to evaluate its dispersion. To take into account any bias related to the F_{ST} estimation in repetitive rich genomic regions, the F_{ST} estimation was repeated excluding windows composed by less than 90% of callable nucleotides, based on the callability annotation.

A global F_{ST} estimate between and within continents was reported in Supplementary Table 14 and showed in Supplementary Figure 62. Using all the genome (F_{ST} [full] in Supplementary Table 14), the level of differentiation between America and Europe was 0.06 (0.02-0.07), a low value indicating that allelic frequencies did not substantially change during the introduction of *P. vulgaris* into Europe from the American continent. Within Europe, the differentiation between the Mesoamerican and Andean genetic pools was high, reaching an F_{ST} of 0.75. Such diversity between gene pools was also higher than what observed in America, where the F_{ST} estimate approached 0.65. Although the mean F_{ST} values were discordant within each continent, the IQR intervals were widely overlapping (0.65-0.93 vs 0.48-0.87). The comparison of the European and American Andean group (EU_A vs AM_A) produced a mean F_{ST} value of 0.07 indicating a modest change in the allele frequencies between the two groups, with an IQR from 0.01 to 0.09. The same comparison tested using the European and American Mesoamerican groups (EU_M vs AM_M), produced a mean F_{ST} value of 0.15 that was higher than the Andean comparison between continents, and an associate IQR interval from 0.02 to 0.24. Also, in this case, despite the fact that the Mesoamerican groups had a genomic F_{ST} distribution that was more shifted towards higher values, the IQR intervals were largely, but not completely, overlapping. This observation suggests that the two pools were differently affected by the process of introduction of *P. vulgaris* into Europe. Correcting F_{ST} by removing windows containing a low proportion of callable regions produced slightly higher estimates and consistent results. The F_{ST} variation along the 11 common bean chromosomes is shown in Supplementary Figure 63.

Supplementary Note 31. Tagging the signatures of adaptation in Europe: excess of introgression

Based on the ChromoPainter results (Supplementary Note 19), the European accessions (*n*=114) were subdivided according to their genome composition into EU_M and EU_A groups, including 43 and 71 individuals, respectively. In absence of demographic and selection forces, each chunk of the genome should be attributed to the corresponding genetic pool from which every individual come from, and hence the frequency of genome segments having a Mesoamerican or Andean ancestry should be equivalent to the frequency of Mesoamerican (f=0.377) or Andean (f=0.623) individuals in Europe. Demographic events, such as admixture between gene pools in European lines, can produce deviations from the expected frequencies and it could be used to identify putative portions of the genome target of excess of introgression. In order to detect deviations from the expected frequencies, the ChromoPainter output was parsed tracing the assignment of each SNP to the corresponding Mesoamerican (AM_M1, AM_M2 and AM_MES) or Andean (AM_A1, AM_A2, AM_A3 and AM_AND) groups, in the 114 European accessions. For each SNP position, the proportion of haplotypes assigned to the Mesoamerican or Andean groups was then computed excluding missing attributions. The raw set of SNPs showing an unexpected proportion of Andean alleles in the sample, constituting a set of genomic candidate positions supporting Andean introgression events into the Mesoamerican genetic pool, was defined extracting genomic coordinates where the observed proportion of Andean assignment was higher than the expected number of Andean haplotypes (EU_A, 71*2=142 haplotypes) plus the 50% of the Mesoamerican ones (EU_M, 43*2*0.5=43) over the total sample of haplotypes ((142+43)/228, Fobs>=0.811). The putative SNPs targets of Mesoamerican introgression events were identified according to the same rationale, checking all the cases in which the Mesoamerican assignment frequency exceeded the threshold given by the expected number of Mesoamerican haplotypes (EU_M, 43*2=86) plus the 50% of Andean ones (EU_A, 71*2*0.5=71, Fobs=0.688). The Bedtools "slop -b 2500" and "merge -d 10000" functions were used to pass from SNP point coordinates to 5Kb regions and then merge it in larger genomic blocks if the relative distance between each of them was lower than 10kb. Only genomic regions supported by at least three SNPs were retained.

The genomic scan resulted in 135 candidate regions (CR) having frequencies exceeding our thresholds (Supplementary Table 15 and Supplementary Data 8). Among them, 131 regions supported the diffusion of Andean alleles into the Mesoamerican gene pool in Europe, whereas the opposite case (Mesoamerican alleles into the Andean gene pool) was observed in only four regions. Andean

introgression was widespread across chromosomes going from the 56.1Kb (Chr02) to 1.09Mb (Chr03) and it was also present at different proportions in other chromosomes. Mesoamerican introgression was limited and concentrated to Chr05 and Chr08 spanning a total of 17.4 and 5.3 Kb, respectively.

Supplementary Note 32. Inter-chromosomal linkage disequilibrium

The level of inter-chromosomal linkage disequilibrium was also evaluated. The VCFtools program was used to sample one SNP every 10kb and compute the r^2 correlation index between pairs of markers coming from different chromosomes. The analysis was performed independently over the AM_M, AM_A, EU_M and EU_A groups, using only SNPs that were segregating within each group of accessions with a minor allele frequency higher than 0.05, and only pairs of SNPs showing an r^2 value >= 0.8 were retained. Multiple pairs of SNPs pointing to the same chromosomal regions were merged if located within 100kb from each other using an in-house script and only pairs of regions spanning at least 500kb at each side were retained. The whole analysis was also repeated including only SNPs falling in the putative regions under selection defined by the “Extended” and the “Restricted” set of regions from the hapFLK analysis, decreasing the minimum width of retained regions from 500kb to 50kb. Link plot showing regions in high linkage disequilibrium were produced using the *Rcircos*[53] package.

The two American groups showed a higher number of inter-chromosomal associations than the European ones (Supplementary Figure 64, Supplementary Data 9, Supplementary Data 10). The AM_M and the AM_A groups had 5,286 and 8,447 links respectively, whereas only 583 links were identified in the EU_M group and 299 in the EU_A accessions. Analyzing the overlapping links identified between the American and the European groups, 286 links were shared between the AM_M and EU_M and 297 links were private of the EU_M group; 211 shared links were also present between the AM_A and the EU_A groups with the occurrence of 88 private links in the latter group.

Focusing on inter-chromosomal LD in the “Extended” set of regions under selection (Supplementary Figure 65, Supplementary Data 11, Supplementary Table 16), 162 and 221 links were identified in the AM_M and AM_A groups, whereas 137 and 18 links in the EU_M and EU_A groups. Sixty-five

links were shared between AM_M and EU_M groups and 72 links were private to EU_M. The AM_A and the EU_A shared 17 inter-chromosomal links whereas one was private of the EU_A group. Approximately half the number of links between chromosomes were identified analyzing the "Restricted" set of regions under selection (Supplementary 66, Supplementary Data 12). One hundred six and 121 links were found in the AM_M and AM_A groups, whereas 74 and 17 links were found in the EU_M and EU_A groups. Fourty-four links were shared between AM_M and EU_M groups and 30 links were private of EU_M. The AM_A and the EU_A shared 17 inter-chromosomal links but none were private of the EU_A group.

Supplementary Note 33. Additional quality filtering of variants for genome wide association studies (GWAS)

In order to perform GWAS, specific additional filters were applied to the samtools/GATK overlapping SNP callset described in the Supplementary Note 8-9. A total of 3,434,655 polymorphic SNPs was maintained after a quality control check performed to maintain only biallelic SNPs across the eleven chromosomes and the available scaffolds. No INDELS, singletons nor private doubletons were kept, as further filtering thresholds a MAF of 0.05, maximum proportion of missing data of 25% and a maximum proportion of heterozygosity of 1% were used. Imputation and phasing were accomplished by using BEAGLE 4.1[24,54]. An LD clumping to remove loci in LD at r^2=1 was performed by using the R package 'bigsnpr'[55] (https://github.com/privefl/bigsnpr). A second dataset composed of 7716 CNVs, including deletions and insertions, (see Supplementary Note 11, Identification of Copy Number Variations), was also used to perform GWAS.

Supplementary Note 34. Models used for GWAS

GWAS was performed using the SNP and CNV datasets for the growth habit (GH), that is scored as presence or absence and coded as 0/1 in the input phenotypic file, and for the PCs that were obtained by the analysis of flowering time and photoperiod sensitivity data (FLW) (see Supplementary Note 6).

A single-locus mixed linear model (MLM), implemented in the MVP R package[56,57] (https://github.com/XiaoleiLiuBio/MVP), was run at first. QQ-plots from MLM results suggested that confounding effects were not completely resolved by kinship and population structure correction. The Bonferroni correction at $\alpha = 0.05$ was set up as the significance threshold for each trait. Even

though the Bonferroni correction might be considered too conservative (see e.g., Cui et *al.*[58]), we used it to limit the occurrence of Type I errors. The analysis was then conducted using the multi-locus stepwise linear mixed-model (MLMM[59]; https://github.com/Gregor-Mendel-Institute/MultLocMixMod) that, using a step-wise approach, includes the most significant SNPs as cofactors to the mixed-model. The mBonf criterion was used to identify the optimal results, and the α = 0.05 Bonferroni-corrected threshold was used. The multilocus models are often used when complex traits are investigated because of the "higher power and lower false discovery rate" when compared to single-locus approaches (see e.g., Segura *et al.*[59] and Wang *et al.*[60]). In particular, the MLMM is based on a stepwise mixed-model regression with forward inclusion and backward elimination of multiple loci and the variance components of the model are tested at each step. The forward inclusion of further loci is stopped when the estimate of the heritable variance of the trait is close to zero or when a maximum number of steps is reached[59]. In this model, the phenotypic variance is subdivided into genetic and residual variance (or error). The genetic explained variance is given by the kinship and the significant SNPs included as cofactors in the model. The unexplained part of the genetic variance can be indicated as the "missing heritability" of the trait.

GWAS models usually assume that the analysed phenotypic traits are normally distributed, but this is rarely observed in nature. To overcome this issue, some researchers normalize the traits distribution, despite adequate transformation methods are often difficult to choose and subsequent interpretation of the results might be problematic[61-63]; some others state that transformation is not necessary when proper attention is given to sample size, heteroscedasticity and minor allele frequency (e.g., Goh and Yap[64], Beasley *et al.*[65], Bůžková[66], Liu *et al.*[67]). We performed the analyses both using unmodified and transformed trait distributions and we compared the results. Normalization of the flowering data was done using different methods: log-transformation, RANK_INT transformation (e.g., Wu *et al.*[63]), and a method implemented in the python package WarpedLMM[61] which is used to normalize the phenotype distributions based on the data. We compared these results with the ones obtained on the unmodified phenotypic distribution by using the MVP package. We observed that the main inflation region in the QQ-plots was not completely removed when transforming the distribution of the phenotypic data, which was on the contrary removed when the MLMM model was used. Thus, we concluded that the inflation of the model was likely due to statistical confounding originating from the polygenic nature of the trait (flowering trait; see e.g., Kobayashi & Weigel[68], Buckler *et al.*[69]) possibly due to multiple loci of "large-effect"[59]. In addition, considering the advantages and limitations of the available GWAS models, many studies recommend the combination of single-locus

and multi-locus models to improve the detection power and robustness of GWAS[70-74]. We have therefore chosen to use the original untransformed data and both the MLMM and the MLM models to analyse and discuss our data.

Supplementary Note 35. GWAS on growth habit

GWA analysis on GH trait (determinate *vs* indeterminate) evidenced a major marker-trait association (MTA) on Chromosome Pv01 at 44871969 bp (p = 2.31E-97) and two additional associations, with lower -log$P$ values, one on Chromosomes Pv01 at 45413334 bp (p = 4.31E-18) and one on Chromosome Pv08 at 6852621 (p = 2.55E-11) (Supplementary Figure 67a). These last two associations were detected after that the main MTA was included as cofactor into the model (Supplementary Figure 67b). The SNP S01_44871969 is located 14 Kb upstream of *Phvul.001G189200* (*PvTFL1y*) (Supplementary Figure 68), the gene identified as responsible for the determinacy trait[75,76] and located in the *fin* locus[77]. This region was also associated to determinacy in a GWAS performed on an Andean population[78].

When the three markers were included into the MLMM model the missing heritability was almost zero, indicating that the trait has a simple Mendelian inheritance, and it can be assumed to be controlled by one or a few genes (Supplementary Figure 67c).

The single-locus MLM analysis on GH pointed to the same genomic region identified by MLMM. Indeed, an association peak composed by numerous SNPs is evident on Chromosome Pv01 around 44-45 Mb (Supplementary Figure 69). Other minor associations are also seen on Chromosomes Pv01, Pv06, Pv07, Pv10 and Pv11.

The GWA analysis based on the polymorphisms due to the CNVs and performed by using a single-locus MLM has acknowledged the results obtained from both the single- and multi-locus analyses. Indeed, two duplications, within a region that overall spans ~45 Kb (46884001-46929000 bp), were found to be significantly associated with the GH trait and they are located ~1.5 Mb downstream from the significant peak detected by the precedent analyses. An additional region (Chr01:25458001_25465500) contained a deletion associated with the same trait (Supplementary Figure 70).

Supplementary Note 36. GWAS on flowering time

GWAS was performed on the first five PCs obtained from a PCA on flowering and photoperiod data that were registered in ten different environments, therefore the results of this analysis are the output of a composite trait. The correlation analysis performed between each flowering PCs and the flowering data (days to flowering - DTF - and photoperiod sensitivity - PS) helps in understanding the GWAS results. Indeed, the PC1 is strongly correlated with DTF and PS data from almost all the environments (Supplementary Figure 71), and it mainly explains the variation between non-flowering and flowering accessions under long-day conditions.

The other principal components further explain the residual variation among genotypes associated to the flowering. In particular, the PC3 is positively correlated to DTF in I1GS6 (Supplementary Figure 71), the short-day experiment performed in Italy, thus capturing the variation between early flowering and late flowering individuals.

The GWA analysis performed by the Multilocus Model, MLMM, pointed to different chromosomal regions as potentially implicated in the expression of this trait (Supplementary Figures 72-73).
In particular, the loci that showed the strongest association with the PC1 (eight overall) were located on Chromosomes Pv03, Pv06, Pv07, Pv09 and Pv11 (Supplementary Figure 72, Supplementary Table 17). GWAS on the remaining components identified 21 additional significant associations located on Chromosomes Pv01, Pv02, Pv03, Pv04, Pv06, Pv07, Pv08, Pv09 and Pv10 (Supplementary Figures 72-73, Supplementary Table 17).

GWAS results from the single- and multi-locus models, were compared and the output from the MLM analysis confirmed many of the significant associations detected by MLMM, with additional MTAs enriching the target regions (Supplementary Figure 74); additional regions were also detected solely by the single locus model.

Subsequently, a single-locus MLM association analysis was performed on the five flowering components by using the CNV as markers. The output of these analyses is illustrated in Supplementary Figure 75.

Significant associations between CNV (both duplications and deletions) were found in genomic regions that were not always overlapping with the ones detected by SNPs from WGS, such as the one

on Chr05. Nonetheless, regions shared by the two analyses were identified on Chromosomes Pv02 (around 42Mb), Pv04 (around 16 Mb), Pv08 (around 18Mb and 60 Mb), Pv10 (around 31 Mb and 37 Mb) and Pv11 (around 7 Mb).

Some of the MTAs for flowering and photoperiod sensitivity here identified, fall within genomic regions that overlap with QTLs detected for the same traits in common bean[33,77,79-83]. In particular, different candidate genes involved in the control of flowering were previously mapped within the region from 44.5 Mb to 48.5 Mb on Chromosome 1[33,77,79,81,82,84], including *PvTFL1y* (*Phvul.001G189200*), the *fin* locus for determinacy[76]. A candidate for the photoperiod sensitivity (*Ppd*) locus was recently proposed by Weller *et al.*[82] to be *Phvul.001G221100*, the red/far-red photoreceptor gene *PHYTOCHROME A3* (*PHYA3*) located at 47.5 Mb. This gene is the orthologous of *E3*, a *PHYA* gene involved in photoperiod responses in soybean[85] and a role in the regulation of flowering in common bean was also suggested by other authors[81,86]. Other common bean genes that represent putative candidates for flowering time and photoperiod sensitivity, based on the presence of adaptive introgression signals (i.e., selection and excess of introgression), and/or GWAS peak and a putative function based on the orthology with known genes associated to flowering, will be discussed in the following sections (see Supplementary Note 40, 41, 46, and 49-52, and Supplementary Data 13).

Supplementary Note 37. Investigation on function of candidate genes for adaptation: Gene annotation and orthofinder analysis

Here, we identified orthologous genes across a set of legume species and *A. thaliana* using the Orthofinder tool[87] with the aim to provide more information for the gene annotation in *P. vulgaris*. For the orthologous identification, the entire protein sequences from *P. vulgaris* (v2.1), *A. thaliana* (TAIR10), *Glyicine max* (Wm82.a2.v1), *Medicago truncatula* (285_Mt4.0v1), *Vigna unguiculata* (v1.1), *Cicer arietinum* (cicar.ICC4958.gnm2.ann1), *Lotus japonicus* (v3.0), *Lupinus angustifolius* (1.0), *Vigna angularis* (vigan.Gyeongwon.gnm3.ann1.3Nz5), *Vigna radiata* (vigra.VC1973A.gnm6.ann1) and *Glycyrrhiza uralensis* were compared[88]. The putative function of not well characterized common bean genes was predicted based on the orthologous relationship coupled with a literature screening of functionally characterized genes in the species that were included in the analysis. From the above species, the orthologue and known genes involved in

flowering time, photoperiod and growth habit were selected and checked if located within the GWAS results (regions found to be associated with flowering time, photoperiod and growth habit).

Supplementary Note 38. Enrichment analysis within DTF and GH QTLs

For the enrichment analysis, genes within a 100 kb interval including 50 kb up and 50 kb down-stream of each significant SNP associated to DTF, and GH, and genes located within selection scan (extended set) and excess of introgression scan regions (see Supplementary Note 21, 31, 35, 36) were considered and subjected to GO term enrichment analysis including biological process (BP), cellular component (CC), and molecular function (MF) using the enrichment analysis available on the Metascape tool[89] (http://metascape.org).

In order to check whether a possible enrichment in gene functions that could be related to DTF traits, 926 genes located within a 100 kb interval of significant GWAS SNPs for DTF were subjected to GO enrichment analysis (Supplementary Figure 76). Results showed that the top highly enriched pathways were involved in morphogenesis of branching structures, cell wall biogenesis, regulation of hormones levels, tissue development, response to UV, and shoot apical meristem. Similarly, 198 genes within the QTLs related to GH trait were subjected to enrichment analysis. Supplementary Figure 76 shows that these regions were mainly enriched with genes involved in ion homeostasis, seeds germination and embryo development, in addition to hormone signaling and carbohydrates processes.

Supplementary Note 39. Gene enrichment analysis in selection scan, regions with significant F_{ST} differentiation and introgression scan

We extended the enrichment analysis to the genes located within all the selection scan (extended set), and to those genes within selection scan showing significant F_{ST} between American and European accessions (Sel_F_{ST}), in order to investigate the putative gene functions of genes associated to recent selection in Europe (Sel_F_{ST}) and to previous selection in America (all the selection scan). Moreover, we investigated the function of genes located in selection scans showing both significant F_{ST} between Europe and America and signature of adaptive introgression (Sel_F_{ST}_excess of Introgr), with the aim to identify regions putatively involved in the adaptation of common bean in Europe, through the combined effect of recent section in Europe and introgression. Supplementary Figure 76 showed the top highly enriched pathways. When we considered all the selection scan, we found significant

enrichment for functions such as negative regulation of seed germination and cellular processes, de-etiolation, phenylpropanoid metabolic processes, anatomical structure formation involved in morphogenesis, response to virus, auxin-activated signaling pathway, vegetative to reproductive phase transition of meristem, cell surface receptor signaling pathway, response to UV, and response to water deprivation. The enrichment analysis for genes in selection scan putatively associated to novel selection in Europe (Sel_F_{ST}), highlight an enrichment for root development in addition to functions already highlighted in the previous analysis. Finally, when look at the adaptive introgression scan (Sel_F_{ST}_excess of introgression), we detected an enrichment for vegetative to reproductive phase transition of meristem, hormone metabolic process, plastid organization, pigment metabolic process, and response to auxin.

Supplementary Note 40. Introduction to the gene function investigation

We identified potential candidate genes at the basis of the adaptation and diversification of the common bean in Europe. We considered as promising candidates those genes located within selection, introgression scans (Supplementary Note 21 and 313), and showing a significant F_{ST} index between American and European accessions (p<0.05) (for a summary on the candidate genes see Supplementary Data 13). Moreover, we identified and investigated those genes located within 50 kb centered on significant GWAS peaks associated to flowering and photoperiod, and to growth habit (GH) (Supplementary Data 13). The putative function in adaptive and diversification processes was inferred according to the gene description in the last common bean genome (Phytozome v.2.1) and according to the function of the orthologous in *A. thaliana* and other legumes species (identified as in Supplementary Note 37). The main genes involved in the key pathways for the regulation of the flowering time in *A. thaliana* are represented in Figure 5; in the figure we emphasized those genes for which we identified orthologous genes in common bean that are located within selection and/ or introgression scans and GWAS peaks.

Supplementary Note 41. Candidates for adaptation in European environments: genes involved in the flowering networks

The timing of flowering, that also depends on the photoperiod sensitivity, is one of the major diversification traits that defines adaptation of plant populations to different agro-ecological conditions. Here, we identified *Phvul.009G259400* and *Phvul.009G259650* in common bean that are

the orthologous to the *LATE ELONGATED HYPOCOTYL* gene (*LHY*) in *A. thaliana* (Supplementary Data 13, rows 90 and 92). *LHY* is a key component of the photoperiodic pathway that affects the flowering time[90]. *LHY* is a pivotal oscillator in the morning stage of the circadian clock, and it is interconnected with *CIRCADIAN CLOCK ASSOCIATED 1* (*CCA1*) in the direct suppression of the middle, evening, and night complex genes[91]. Interestingly, there are evidence of strong functional similarities between many *A. thaliana* clock genes and their homologs in legume species, suggesting a conservative and analogous role of *LHY* in *P. vulgaris*[92-94]. Among these genes, *LUX ARRYTHMO* (*LUX*) encodes a night-time repressor essential for the transcriptional regulation of the circadian rhythms[95-97]. *LUX* is coregulated with *TIMING OF CAB EXPRESSION 1* (*TOC1*) and repressed by *CCA1* and *LHY* by the direct binding of these proteins to the *LUX* promoter[98,99].The *LUX* orthologous in the common bean (*Phvul.011G062100*) is under selection, however, we did not detect a significant F_{ST} value between American and European accessions and it does not fall within an introgression scan, suggesting the occurrence of a pre-existing selection in the American germplasm for this gene (Supplementary Data 13, row 98). *VRN* genes mediate vernalization, the process by which the exposure to low temperatures during the winter season allows plants to reach vegetative maturity before reproduction. *Phvul.011G050600* is orthologous to the *A. thaliana VERNALIZATION 1* (*VRN1*) and *RELATED TO VERNALIZATION1 1* (*RTV1*) genes (Supplementary Data 13, row 97). In *A. thaliana*, *VRN1* and *RTV1* are two key genes for the activation of the floral integrator genes after the exposure to long-term cold temperatures. In detail, overexpression of *RTV1* led to early flowering through the increased expression of floral integrator genes, such as *FT* and *SOC1*[100]. With regard to *VRN1,* it encodes a MADS box transcription factor related to the *A. thaliana* proteins *FRUITFULL* (*FUL*) and *APETALA1* (*AP1*). *VRN1* promotes flowering by decreasing the level of the *FLOWERING LOCUS C* (*FLC*) transcript and protein[101] (Figure 5). *FLC* encodes a transcription factor that functions as a main inhibitor of the floral transition through the negative control of *SOC1* and *FT* expression[102] (Figure 5). Even though the complete molecular mechanism of *VRN1* is not well addressed to date in *A. thaliana,* being part of the polycomb complex 1 (PCR1), it also interacts with *DRIP1/2* genes which encode C3HC4 RING-domain-containing ubiquitin E3 ligase capable of interacting with DREB2A proteins[103,104]. Interestingly, DRIPs are required to promote all developmental phase transitions and to control cell proliferation during organ growth and development, and they are also involved in regulating stress-related transcriptional changes and drought tolerance[105,106]. *Phvul.001G157400* and *Phvul.007G177500* are orthologous to the *A. thaliana DRIP1/2* genes, and they have been identified within a selection scan with a significant F_{ST} value; moreover *Phvul.007G177500* is located within an introgression scan, while *Phvul.001G157400* falls within a

GWAS peaks for the days to flowering (Supplementary Data 13, rows 4 and 70). Similarly, to *A. thaliana*, in the temperate cereals, barley and wheat, vernalization is required to ensure flowering under favorable environmental conditions. During vernalization, the transcription of *VRN1* is increased. *VRN1* promotes inflorescence development and represses the transcription of *VERNALIZATION 2* (*VRN2*)[107]. This latter blocks the expression of *FT*-like genes under long day conditions; in turn, short day conditions lead to the repression of *VRN2*, allowing *FT1* expression that promotes flowering in summertime[107,108]. *Phvul.003G191900* has been identified as under positive selection, within an excess of introgression and showing a significant differentiation (F_{ST} index) between American and European accessions; interestingly, it is orthologous to two *A. thaliana* genes involved in the vernalization pathway that are *UBC1* and *UBC2* (Supplementary Data 13, row 50). In *A. thaliana,* the double mutant *ubc1-1 ubc2-1* shows a significative reduction in the number of rosette leaves and an early-flowering phenotype, that confirms their redundant role in promoting the expression of the *FLC* gene[109]. Up to now, eight *A. thaliana* genes have been identified in the autonomous pathway[110] (Figure 5), and among them the *LUMINIDEPENDENS* (*LD*) gene, which is orthologous to *Phvul.001G204600* (Supplementary Data 13, row 29). Mutants for this gene displayed late flowering and reduced expression of *LFY*, that confirms the role of *LD* in the floral transition by the repression of *FLC*[111,112] (Figure 5). The gibberellic acid (GA) pathway is also one of the four central networks involved in the regulation of the flowering time in *A. thaliana*. GA promotes petal and stamen filament elongation and controls the cellular developmental pathway of anthers[113]. Downstream to GA signaling, the paralogous and functionally redundant GATA transcription factors GATA, NITRATE-INDUCIBLE, CARBONMETABOLISM INVOLVED (*GNC*) and CYTOKININ-RESPONSIVE GATA FACTOR1 (*CGA1*) directly downregulate the *SOC1* expression and consequently repress the flowering[114]. In turn, both *GNC* and *CGA*, which are also positive regulators of chlorophyll biosynthesis and chloroplast division[115-117], are negatively regulated by *SOC1* to modulate cold tolerance and greening[114].*Phvul.003G137100* is the orthologous to the *A. thaliana GNC* and *CGA* genes (Supplementary Data 13, row 38). Finally, *Phvul.001G154800*, *Phvul.001G204700* and *Phvul.011G074100* (Supplementary Data 13, rows 3, 30 and 99) are the orthologous of the *A. thaliana INDETERMINATE DOMAIN 8* (*IDD8*) gene, commonly known as *NUTCRACKER* (*NUC*). In detail, *Phvul.001G204700* shows all the adaptive signals, while *Phvul.001G154800* and *Phvul.011G074100* shows only GWAS signals for the flowering.

NUC belongs to a secondary pathway that interacts with the main regulatory flowering networks. In detail, it encodes for a transcription factor that positively regulates the photoperiodic flowering by

modulating the sugar transport and metabolism via the *FT* gene[118,119]. *NUC* activates the sucrose synthesis by binding directly to the promoter of the *SUCROSE SYNTHASE 4* (*SUC4*) gene, resulting in promoted flowering in SUS4-overexpressing plants. However, it is still unclear how the *NUC* modulation of sugar metabolism and transport influences FT expression[119]. In addition, *NUC* is a direct target of *SHR* transcription factor, which plays key roles in specifying the root stem cell niche and radial root patterning[120].

Supplementary Note 42. Candidates for adaptation in European environments: genes involved in root development and traits

Variations in the root structure architecture (RSA) can allow plants to explore different environments and agro-ecological conditions that result also in a better adaptation to novel environments. *SHORT ROOT* (*SHR*) encodes a transcription factor belonging to the GRAS gene family, and it is orthologous to *Phvul.002G010700* (Supplementary Data 13, row 36). *SHR* is a key root mitotic regulator and it is essential for maintaining root apical meristem (RAM) activity[121,122]. Moreover, *SHR* is required for the normal shoot gravitropism, initiation and patterning of lateral root primordia, and the maintaining of the indeterminate growth of lateral and adventitious roots[123]. *Phvul.009G238200* (Supplementary Data 13, row 81) is another promising candidate and it is orthologous to *VILLIN 4* (*VLN4*). *VLN4* regulates the organization of long axial and short apical actin bundles in root hairs, and it is essential for normal root hair growth and cytoplasmic streaming within root hairs[124]. Another candidate gene is *Phvul.001G202600* (Supplementary Data 13, row 9) that is orthologous to the *TRANSPORT INHIBITOR RESPONSE 1* (*TIR1*). *TIR1* encodes an auxin receptor that mediates auxin-regulated transcription, and it is consequently involved in several physiological processes[125]. In particular, it has been demonstrated that *tir1* mutants fail the hypocotyl elongation and lateral root formation[126].

Supplementary Note 43. Candidates for adaptation in European environments: genes involved in responses to environmental stresses

We identified several *P. vulgaris* candidate genes based on the homology with proteins involved in environmental stress responses in *A. thaliana* and on the presence of selection signatures and introgression (Supplementary Data 13). Among those, *OVARIAN TUMOR DOMAIN-CONTAINING DEUBIQUITINATING ENZYME 5* (*OTU5*) is a phosphate-responsive gene that acts downstream of

phosphate signaling, and it is orthologous to *Phvul.001G203400* (Supplementary Data 13, row 16). In phosphate (Pi) starvation conditions, *OTU5* is responsible of adaptive responses recalibrating and maintaining cellular Pi homeostasis. Insufficient phosphate supply causes alterations in root architecture and epidermal cell morphogenesis; indeed, *otu5* mutants show short-root-hair phenotype and decreased primary root growth[127,128]. *Phvul.005G010500* (Supplementary Data 13, row 58) is also an interesting candidate, and its orthologous *BIOTIN F* (*BIOF*) in *A. thaliana* encodes a 7-keto-8-aminopelargonic acid (KAPA) synthase, the first committed enzyme of the biotin synthesis pathway[129]. In addition to its essential metabolic functions, *BIOF* is involved in survival pathways by modulating the defense genes expression and spontaneous cell death[130]. *Phvul.002G300900* (Supplementary Data 13, row 37) is orthologous to the *A. thaliana SWEET6* and *SWEET7* genes. *SWEET* proteins represent one of the largest sugar transporter family in the plant kingdom and play crucial roles in plant development and stress responses[131,132]. Interestingly, Gautam *et al.*[132] identified 108 SWEET genes across the entire wheat (*Triticum aestivum*) reference genome (IWGSC RefSeq v1.0), and expression analysis revealed higher expression of SWEET genes in water and heat sensitive and leaf-rust resistant genotypes. Similarly, Zhang *et al.*[133] identified in *Brassica oleracea* five SWEET genes with reduced expression levels under chilling stress. Additionally, the expression levels of six SWEET genes were up-regulated in roots of a clubroot-susceptible cabbage cultivar after inoculation with *Plasmodiophora brassicae* compared with uninoculated plants[133]. Overall, these recent results clearly indicate that SWEET genes play conservative and important roles in promoting sugar transport to enhance abiotic tolerance and disease resistance in plants. Finally, *Phvul.008G024400* (Supplementary Data 13, row 75) is orthologous to the *GENOMES UNCOIPLED 1* (*GUN1*) gene in *A. thaliana*, which is implicated in plant adaptation to long-term high-light stress via ROS accumulation and ROS-dependent signaling[134].

Supplementary Note 44. Candidates for adaptation in European environments: additional genes

Phvul.009G259100, *Phvul.009G259200*, *Phvul.009G259250* and *Phvul.009G259500* encode for caffeic acid 3-O-methyltransferases (Supplementary Data 13, rows 86, 87, 88 and 91). Caffeic acid is a secondary metabolite, primarily required during the synthesis of the lignin polymer[135]. Furthermore, it is involved in the regulation of cell expansion, turgor pressure, phototropism, water flux, and growth[136]. Interestingly, caffeic acid and its derivatives are also involved in plant biotic and abiotic stress tolerance including responses to pathogen attacks to low and high temperature stress,

UV light, drought, heavy metal and salinity stress[137].*Phvul.001G258700* (Supplementary Data 13, row 33) is another interesting gene that is orthologous to the *A. thaliana AT1G13450* gene. *AT1G13450* encodes GT-1, a plant transcription factor that binds to one of the cis-acting elements, BoxII, which resides within the upstream promoter region of light-responsive genes, and it was assumed to act as a molecular switch modulated through Ca(2+)-dependent phosphorylation/dephosphorylation in response to light signals[138]. Finally, the orthologous of *Phvul.006G073500* (Supplementary Data 13, row 64) in *A. thaliana* is the *PHOTOSYSTEM II CORE PHOSPHATASE* (*PBCP*) gene, which encodes a chloroplast PP2C phosphatase that is required for efficient dephosphorylation of PSII proteins and involved in light acclimation[139].

Supplementary Note 45. Secondary candidate genes

Here, we report about additional candidate genes that are located within 50 kb centered on significant GWAS peaks for flowering and photoperiod or growth habit (GH) traits (see also Supplementary Note 35 and 36), that do not show the three signals for the adaptation to Europe (i.e., selection, significant F_{ST} index between American and European accessions (p<0.05), and excess of introgression). However, based on the GWAS signal and/or the function of the orthologous, those genes are potential candidates for relevant biological processes. Detailed information for each gene is reported in Supplementary Data 13.

Supplementary Note 46. Secondary candidate genes: genes involved in the flowering networks

UBP12 and *UBP13* are orthologous to *Phvul.007G234000* (Supplementary Data 13, row 73), and they are two ubiquitin-specific proteases that act directly in the control of the circadian clock and, through CO-dependent pathway, in the photoperiodic flowering[140,141]. In *A. thaliana CO* is a photoperiod-dependent flowering time locus that encodes a zinc-finger transcription factor downstream of photoreceptor and circadian clock genes[142]. *CO* is activated under long-day condition and it up-regulates the expression of the *FLOWERING LOCUS T* (*FT*) gene to promote flowering[143]. Another gene of interest is the *PHY-INTERACTING FACTOR 1* (*PIF1*), also named *PHY-INTERACTING FACTOR 3 LIKE 5* (*PIL5*), that is orthologous to *Phvul.001G168700* (Supplementary Data 13, row 5) in common bean. During long-day conditions, *PIL5* interacts with the master genes in the regulation of the transition from the vegetative to the flowering stage by

negatively regulating *FT*, *SUPPRESSOR OF OVEREXPRESSION OF CO 1* (*SOC1*), and *LEAFY* (*LFY*)[144]. *Phvul.008G133600* (Supplementary Data 13, row 78) is orthologous to the *A. thaliana CIB2* gene, which also belongs to the photoperiod pathway (Figure 5). *CIB2* encodes for a basic helix-loop-helix (bHLH) DNA-binding protein, and in *A. thaliana* it specifically interacts with *CRYPTOCRHOME 2* (*CRY2*) in regulating the response to blue light and to promote the transcription of the main floral integrator *FT*[145]. Another candidate is *Phvul.006G109600* (Supplementary Data 13, row 65), which is orthologous to the *A. thaliana LIGHT-RESPONSE BTB1* gene (*LRB1*). *LRB1* encodes for nucleus localized *BTB* proteins that strongly influence photomorphogenesis (i.e., seed germination, cotyledon opening and expansion, chlorophyll accumulation, shade avoidance, and flowering time). In detail, *LRB1* acts redundantly with *LRB2* as negative regulators of the *PHYB/D* (*PHYTOCROME B/D*) turnover and signaling[146]. *Phvul.001G230500* is one of the genes located within a significant GWAS peak for the growth habit (Supplementary Data 13, row 31). It has four orthologous genes in *A. thaliana* (i.e., *GAI*, *RGL1*, *RGA1*, and *RGL2*) that are members of the DELLA proteins, a family of nuclear growth repressors that function in contrast with GA regulation by repressing plant flowering through different genetic interactions. For instance, DELLAs directly activate the expression of *FLC*, and negatively regulate the meristem identity gene *LFY*[147] (Figure 5). Another putative candidate belonging to the gibberellin network is *Phvul.006G120700* (Supplementary Data 13, row 66), that is orthologous to the *A. thaliana GIBBERELLIN 2-OXIDASE 4* gene (*GA2OX4*). *GA2OX4* encodes a gibberellin 2-oxidase that acts on C19 gibberellins. The expression of *GA2OX4* is responsive to cytokinin and *KNOX* activities related to the regulation of the shoot apex of *Arabidopsis*[148]. The shoot meristem identity gene named *TERMINAL FLOWER 1* (*TFL1*) is also an interesting candidate, as a mutation in this gene results in early flowering *A. thaliana* plants that are characterized by the development of a terminal floral meristem[149,150]. Noteworthy, the functional orthologous of *TFL1* in common bean (i.e., *Phvul.001G189200*; *PvTFL1y*) is the gene responsible for naturally occurring variation for determinate growth habit[76] (Supplementary Data 13, row 8). The orthologous of *Phvul.008G142400* in *A. thaliana* is the *CRYPTIC PRECOCIOUS* (*CRP*) gene, which is another flowering regulator that plays multiple roles in the flowering pathway (Supplementary Data 13, row 79). *CRP* represses *FLC* expression, promotes *FT* expression independently of *FLC*, and it also acts downstream of *FT* in the regulation of *SOC1* and *FUL* genes[151]. The *LATE MERISTEM IDENTITY1* (*LMI1*) gene, orthologous to *Phvul.001G184800* (Supplementary Data 13, row 6), encodes a homeodomain leucine zipper class I (HD-Zip I) meristem identity regulator. *LMI1* acts downstream of *LFY*, which is a key gene responsible for the initiation of the flowers, and promotes the expression of *CAULIFLOWER* (*CAL*), which positively regulates *LFY*

expression. The interaction between *LFY*, *LMI1* and *CAL* resembles a feed-forward loop transcriptional network motif[152,153]. Finally, *Phvul.004G085100*, *Phvul.004G085400* and *Phvul.004G085594* (Supplementary Data 13, row 53, 54 and 56) are the orthologous of the *A. thaliana SUCROSE-PROTON SYMPORTER 9* (*SUC9*), which is a gene involved in the carbohydrate metabolism. *SUC9* is highly expressed in sink tissues, and plants containing mutations in this gene show an early flowering phenotype under short-day conditions[154]. Sivitz *et al.*[154] suggest that this gene may prevent premature flowering by maintaining a low concentration of extracellular sucrose.

Supplementary Note 47. Secondary candidate genes: genes involved in responses to environmental stresses

Phvul.008G182500 (Supplementary Data 13, row 80) is located within a GWAS peak associated to the flowering, and it might be involved in environmental stress responses. It is orthologous to the *A. thailana SICKLE* (*SIC*) gene, which is a unique factor required for the biogenesis of some miRNAs, and it is important for the correct plant development and for the modulation of abiotic stress responses. Mutants in this gene are hypersensitive to chilling and salt stresses[155]. Interestingly, *sic* mutants also show low-amplitude or arrhythmic expression of core circadian clock genes under cool ambient temperature cycles, suggesting undirect interactions with *LHY* and *CCA1* genes[156].

Supplementary Note 48. Secondary candidate genes: additional genes

The orthologous to *Phvul.008G041500*, located withing a GWAS window for flowering (Supplementary Data 13, row 77) is *MYB30*, firstly characterized for its role as a positive regulator of the pathogen-induced hypersensitive response and of brassinosteroid and abscisic acid signaling in *A. thaliana*[157]. In addition, *MYB30* is involved in the regulation of the flowering time by directly promoting the expression of the *FT* gene[158]. Recently, Mabuchi *et al.*[159] showed that *MYB30* is also required for root growth regulation during defense responses. Thus, *MYB30* may be a candidate to mediate crosstalk between gene networks involved in biotic stress perception and flowering time. *Phvul.007G071500* (Supplementary Data 13, row 67) is orthologous to the *CYP715A1* gene in *A. thaliana*, which functions as a key regulator of flower maturation by synchronizing petal expansion and volatile emission. Thus, *CYP715A1* appears to be an important determinant of flower-insect interaction[160].

Supplementary Note 49. Candidate genes in regions showing inter-chromosomal linkage disequilibrium and adaptive introgression

The "extended" set of genomic regions with evidence of inter-chromosomal LD private to the EU_A (i.e., European Andean) and to the EU_M (i.e., European Mesoamerican) pools were also considered for the identification of genomic regions potentially involved in the adaptation of common bean in Europe (see also Supplementary Note 32). Here, we considered the genomic regions showing inter-chromosomal LD private to EU_M (see **Supplementary Data 9**) and EU_A (**Supplementary Data 10**) that intersect selections scan windows (i.e., extended set). Among these regions, we particularly focused on pairs of regions in LD for which both the two regions, that are in linkage, show intersection with an introgression scan, with the aim to identify the genetic basis of potential adaptive introgression in Europe. The inter-chromosomal LD between regions from the "extended" set and private to EU_M and EU_A pools are shown in the figure Supplementary Figure 65.

For the identification of candidate genes for adaptive introgression of common bean in Europe, we considered a surrounding region of 50 kb upstream and downstream to the effective start/end positions of the two regions in LD, in order to cover a higher confidence interval.

Supplementary Note 50. Private LD in the EU_M pool

Epistatic interactions between genes belonging to different flowering-related pathways potentially drive the adaptation for flowering time of the common bean in Europe. Here we identified two regions on chromosomes Pv09 (37895653-37988961) and Pv11 (4439224-4507451) showing inter-chromosomal LD, that carry 15 and 24 genes, respectively, with both regions showing signatures of selection and presence of adaptive introgression. Moreover, a QTL for the days to flowering (i.e., GWAS for the PCA1 associated to the flowering time; see Supplementary Note 36) was identified on chromosome Pv11, with a significant SNP (i.e., S11_4451847) located ~46 kb upstream to the orthologous to *VRN1* (i.e., *Phvul.011G050600*) that is a key gene in the vernalization pathway[101] (Figure 5). Interestingly, in the region on chromosome Pv09 that is in LD with the *VRN1* locus on Pv11, we identified the orthologous genes to *LHY* (i.e., *Phvul.009G259400* and *Phvul.009G259650*) that are annotated as "MYB-related transcription factor LHY" on the last common bean genome

version (Phytozome v.2.1). *LHY* is involved in the regulation of the circadian rhythm with a central role in the regulation of the photoperiod pathway in *A. thaliana*[90,91] (Figure 5).

We also detected two other regions on chromosomes Pv01 (46125558-46295360) and Pv03 (33526730-33615190) that show inter-chromosomal LD. In the region on chromosome Pv01 we detected 37 genes among which we identified the orthologous to *LD* (i.e., *Phvul.001G204600*) and *NUC* (i.e., *Phvul.001G204700*); while in the region on chromosome Pv03 we found a total of 16 genes, and among them the orthologous gene to *GNC* and *CGA1* (i.e., *Phvul.003G137100*). As described previously, *LD* gene is one of the eight genes identified so far in the autonomous pathway of *A. thaliana* that acts as a repressor of *FLC* gene, promoting consequently the transition from vegetative to the flowering stage; while *NUC* encodes a transcription factor that positively regulates the photoperiodic flowering by modulating the sugar transport and metabolism via the *FT* gene[118,119]. Both these genes (i.e., *LD* and *NUC*) are positive regulators of the flowering-genes expression, and their orthologous in the common bean (i.e., *Phvul.001G204600* and *Phvul.001G204700*) are in epistatic correlation with *Phvul.003G137100*, whose orthologous genes are *GNC* and *CGA1*. In *A. thaliana* model system, the paralogous *GNC* and *CGA1* act in a redundant way promoting greening downstream from the gibberellic acid network signaling. In particular, they act upstream from the flowering time regulator *SOC1* to directly repress its expression and thereby repress the flowering[114]. Overall, our results suggest that genes belonging to different pathways (i.e., *LHY*, *VRN1*, *LD*, *NUC*, *GNC* and *CGA1*) may have played a relevant role in the adaptation of the common bean in Europe through a possible epistatic co-regulation of flowering and photoperiod sensitivity.

Supplementary Note 51. Additional loci in LD in the EU_M pool

Among the regions showing inter-chromosomal LD private to the EU_M pool, we identified several loci carrying potential candidate genes for adaptation. Within the two regions displaying inter-chromosomal LD on chromosomes Pv01 (46125558-46295360) and Pv03 (33526730-33615190) that were mentioned above, we detected two genes, both involved in root development-related traits. In particular, on Pv01 we found *Phvul.001G202600* that is orthologous to the *TRANSPORT INHIBITOR RESPONSE 1* (*TIR1*). In *A. thaliana TIR1* mediates auxin-regulated transcription, and it is primarily involved in the development of lateral root formation[126]. *TIR1* is in LD with *CLAVATA3/ESR-RELATED 19* (*CLE19*), a member of a large family of putative ligands homologous to the *CLAVATA3* gene (*CLV3*). Casamitjana-Martínez *et al.*[161] demonstrated that localized overexpression in roots of

CLE19 can partially replace the function of *CLV3* in the regulation of the root meristem size through an SCR and SHR-independent pathway. *CLE19* acts by over activating an endogenous CLV-like pathway involved in root meristem maintenance without directly interfering with organizer and stem cell specification.

On chromosome Pv05 we identified a genomic region (10102074-10333357) carrying *Phvul.005G054680* gene, annotated as a SERINE CARBOXYPEPTIDASE-LIKE 36-RELATED. Interestingly, although no detailed characterization is available for the orthologous of *Phvul.005G054680* in *A. thaliana* (i.e., *AT2G35770*), Wen et *al.*[162] showed that the overexpression of *ECS1,* coding for a SERINE CARBOXYPEPTIDASE, results in the increase in the number of carpel and seeds in *A. thaliana*; moreover, plants overexpressing *ECS1* on a *bri1-5* (*brassinosteroid-insensitive 1)* background show earlier flowering time compared to the *bri1-5* mutant, which flower 7–10 days later than wild-type[162]. *Phvul.005G054680* is in inter-chromosomal LD with a region on chromosome Pv09 (37907918-37988961) containing a cluster of 7 genes (i.e., *Phvul.009G258700, Phvul.009G258800, Phvul.009G258900, Phvul.009G259000, Phvul.009G259100, Phvul.009G259200*, *Phvul.009G259250*) that are classified as caffeic acid 3-O-methyltransferase. These latter genes are putatively involved in the phenylpropanoid biosynthesis pathway and therefore involved in the control of water flux, growth, phototropism, cell expansion, and turgor pressure[136]. Recently, Riaz *et al*.[137] reported evidence for the involvement of caffeic acid and its derivatives also in plant biotic and abiotic stress tolerance, including responses to pathogen attacks to low and high-temperature stress, UV light, drought, heavy metal and salinity stress.

Interestingly, the seven caffeic acid 3-O-methyltransferase genes mentioned above are also in LD with another region on chromosome Pv05 (40744434-40800531) containing *Phvul.005G183600,* that is orthologous to the *A. thaliana GA3* gene. *GA3* encodes a member of the CYP701A cytochrome p450 family that is involved in the later steps of the gibberellin biosynthetic pathway[163]. Notably, gibberellins are plant hormones, which are involved in many aspects of plant growth and development, including seed germination, hypocotyl elongation, chlorophyll biosynthesis, and especially flowering induction[113].

Supplementary Note 52. Private LD in the EU_A pool

Here, we identified only one pair of regions from the extended set of regions, showing inter-chromosomal LD private to the EU_A pool. Both two regions, on chromosome Pv05 (10102074-10333238) and on chromosome Pv06 (1584954-1649852), partially overlap with genomic regions on chromosomes Pv05 (10102074-10333357) and Pv06 (1585169-1649852) that are also involved in private inter-chromosomal LD in the EU_M gene pool. This suggests that these loci have a potential role in adaptive strategies both in Mesoamerican and Andean European accessions.

In these regions we found several interesting genes. In particular, *Phvul.005G054680* is annotated as SERINE CARBOXYPEPTIDASE-LIKE 36-RELATED on the last common bean genome version (Phytozome v.2.1), while *Phvul.006G006600* is classified as CLAVATA3/ESR (CLE)-RELATED PROTEIN, and it is involved, together with *WUSCHEL*, in the signaling pathway associated to cell proliferation and differentiation at the shoot apical meristem[164]. Interestingly, although no detailed characterization is available for the orthologous of *Phvul.005G054680* in *A. thaliana* (i.e., *AT2G35770*), Wen *et al.*[162] showed that the overexpression of *ECS1*, coding for a SERINE CARBOXYPEPTIDASE, results in the increase in the number of carpel and seeds in *A. thaliana*; moreover, plants overexpressing *ECS1* on a *bri1-5* (*brassinosteroid-insensitive 1)* background show earlier flowering time compared to the *bri1-5* mutant, which flower 7–10 days later than wild-type[162].

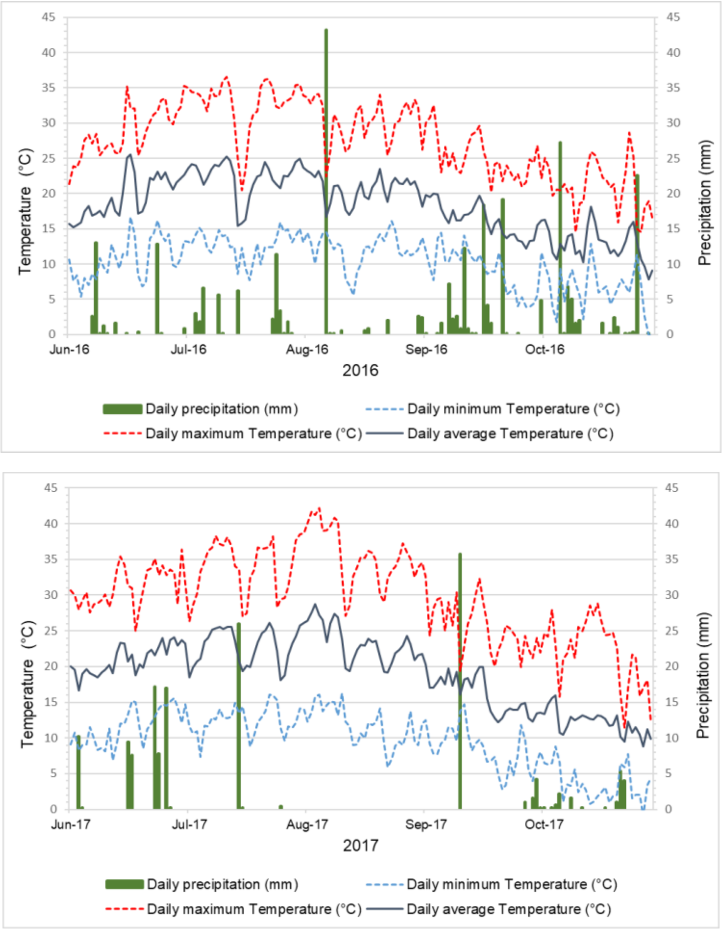

Supplementary Figure 1. Meteorological data at the study site during the common bean growth seasons 2016 and 2017. Data are taken from the nearest weather station in Villa d'Agri – Marsicovetere (Potenza, Italy).

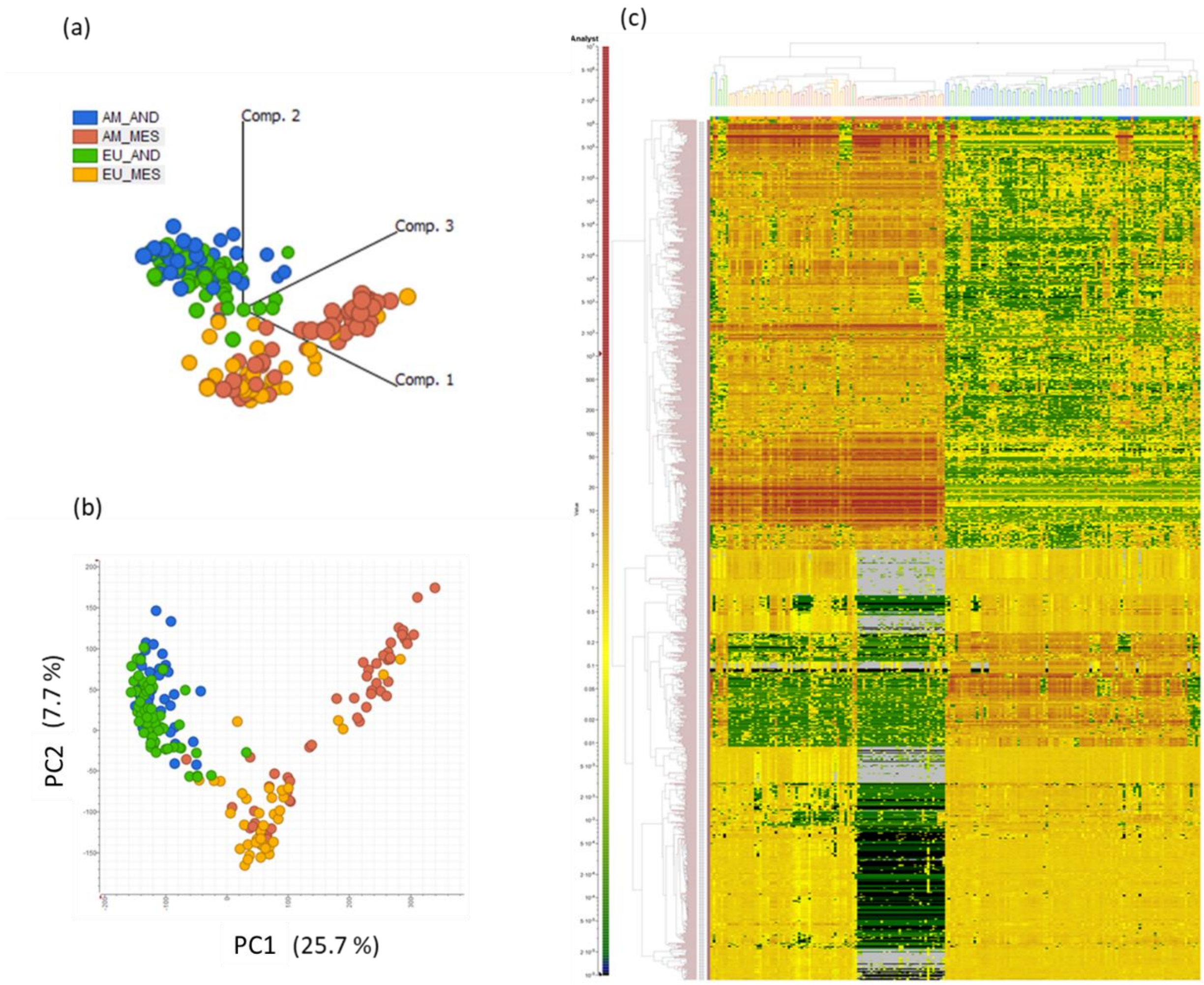

Supplementary Figure 2. Principal component analysis (PCA) and hierarchical clustering heatmap of secondary metabolite profiling of 190 accessions representing four groups; American Mesoamerican (AM_MES), American Andean (AM_AND), European Mesoamerican (EU_MES) and European Andean (EU_AND). (**a**) 3D PCA, projection of the first three Principal Components using 8417 mass features measured by UPLC-MS. (**b**) PCA1 and PCA2 differentiate the metabolic features of common bean accessions across four groups. The amount of variance explained is shown in parentheses on each axis. (c) Heatmap analysis showing the metabolite changes in the top 1000 mass features based on PCA analysis.

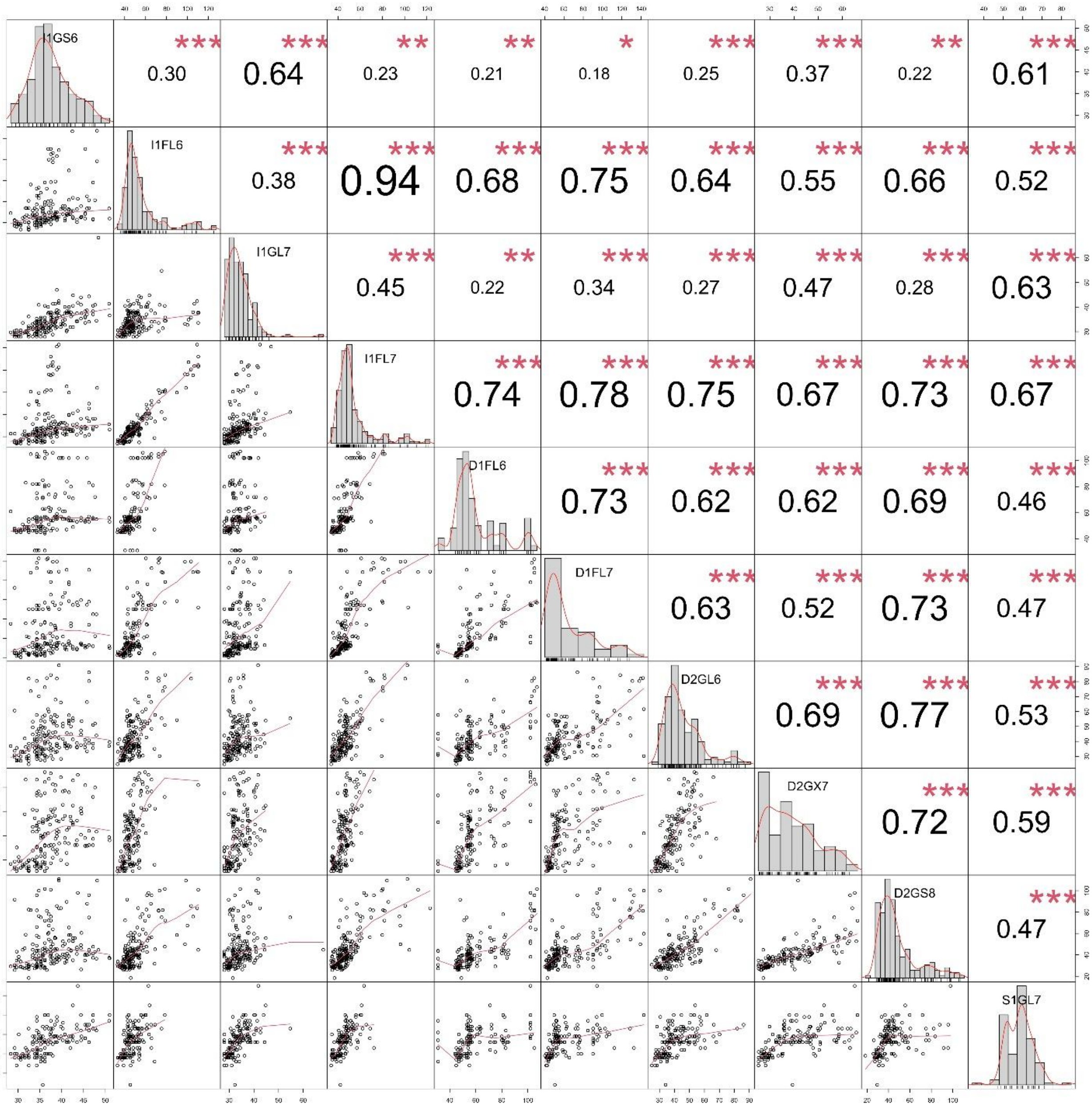

Supplementary Figure 3. Flowering time variation among accessions and across locations. Distribution of the DTF for each environment (calculated using only American and European flowering accessions) are illustrated along the diagonal of the figure. Below diagonal the bivariate scatter plots with a fitted line are displayed for each pairwise comparisons. Above diagonal are indicated the Pearson's correlation values and the associated two-sided *p* values represented as stars (* $p < 0.05$, ** $p < 0.01$, *** $p < 0.001$).

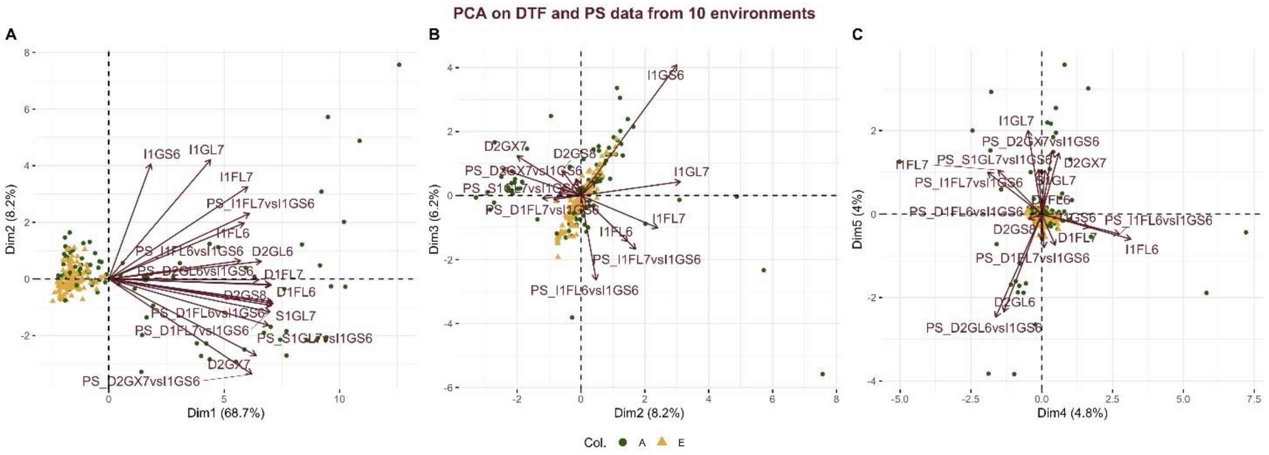

Supplementary Figure 4. PCA on flowering dates and photoperiod sensitivity as registered in ten different environments. The biplots summarize the distribution of the American (green dots) and European (yellow dots) accessions in the space of the PC1 and PC2 (A), PC2 and PC3 (B) and PC4 and PC5 (C). The loading of each variable is indicated by a dark-red arrow starting from the center of the plot.

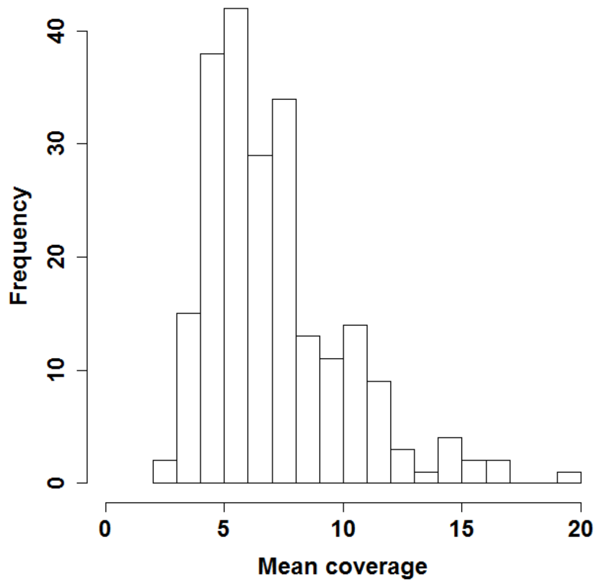

Supplementary Figure 5. Distribution of mean mapped coverages among samples.

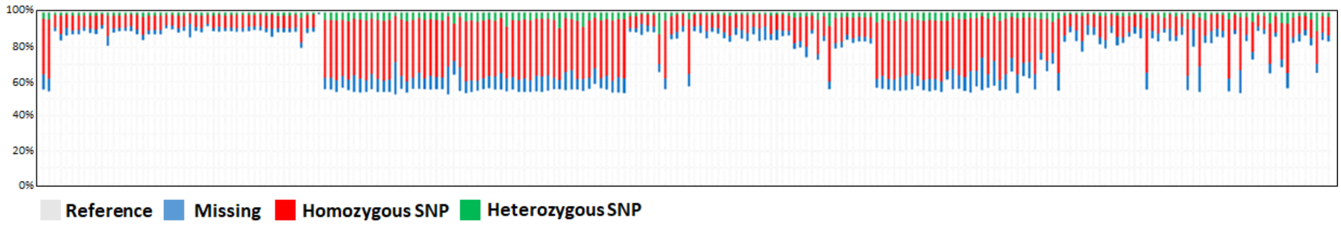

Supplementary Figure 6. Proportion of genotypes in all samples ordered according to the BEAN_ADAPT code. The percentage of missing, homozygous and heterozygous SNPs is shown.

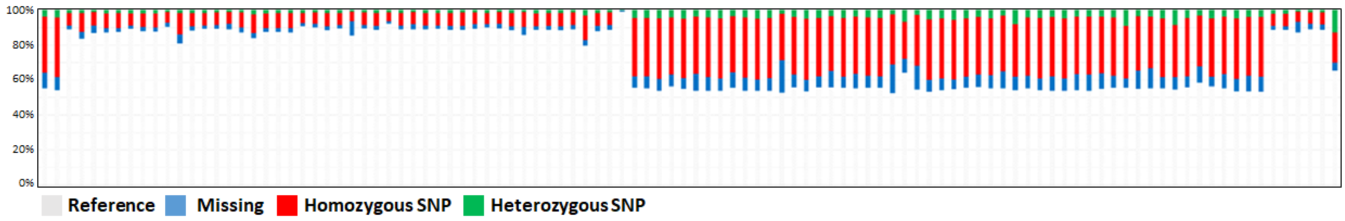

Supplementary Figure 7. Proportion of genotypes in AM samples ordered according to the BEAN_ADAPT code. The percentage of missing, homozygous and heterozygous SNPs is shown.

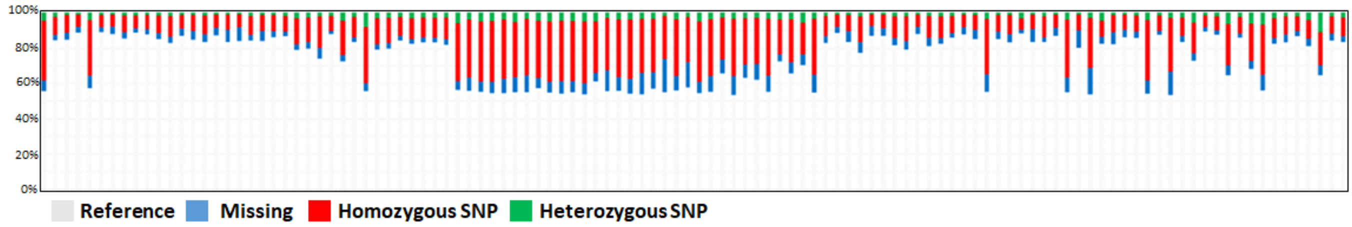

Supplementary Figure 8. Proportion of genotypes in EU samples ordered according to the BEAN_ADAPT code. The percentage of missing, homozygous and heterozygous SNPs is shown.

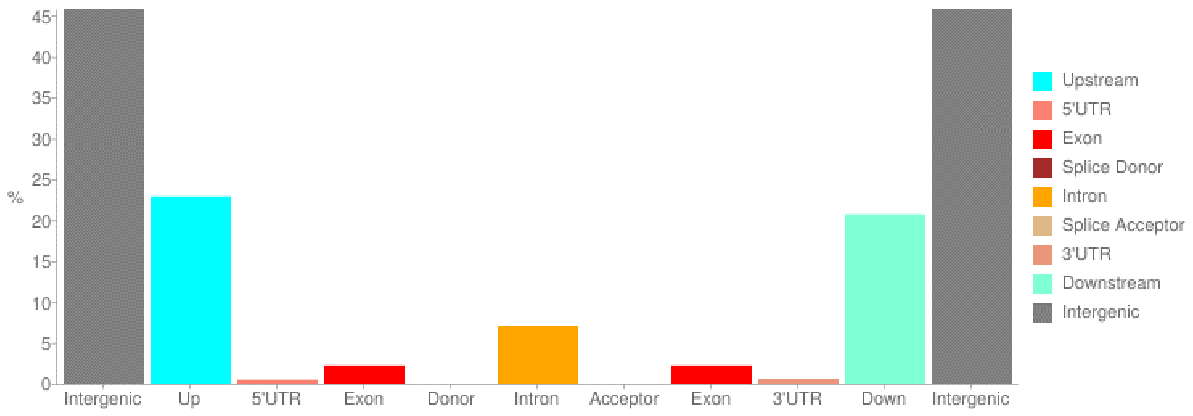

Supplementary Figure 9. Percentage of SNP effects by region.

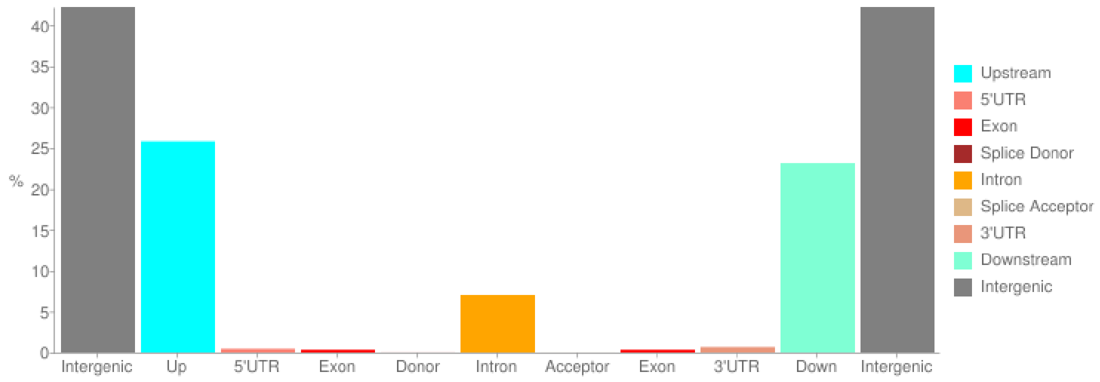

Supplementary Figure 10. Percentage of indel effects by region.

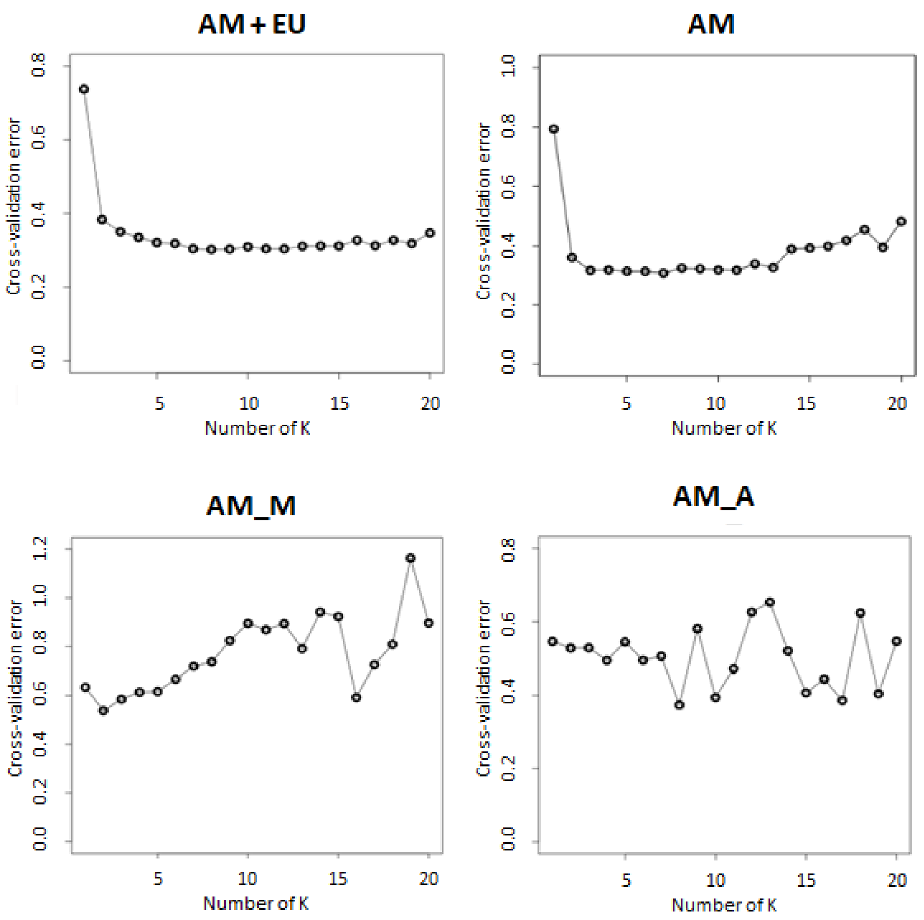

Supplementary Figure 11. Cross-validation error plot from K 1 to 20 for the 4 group of accession analysed.

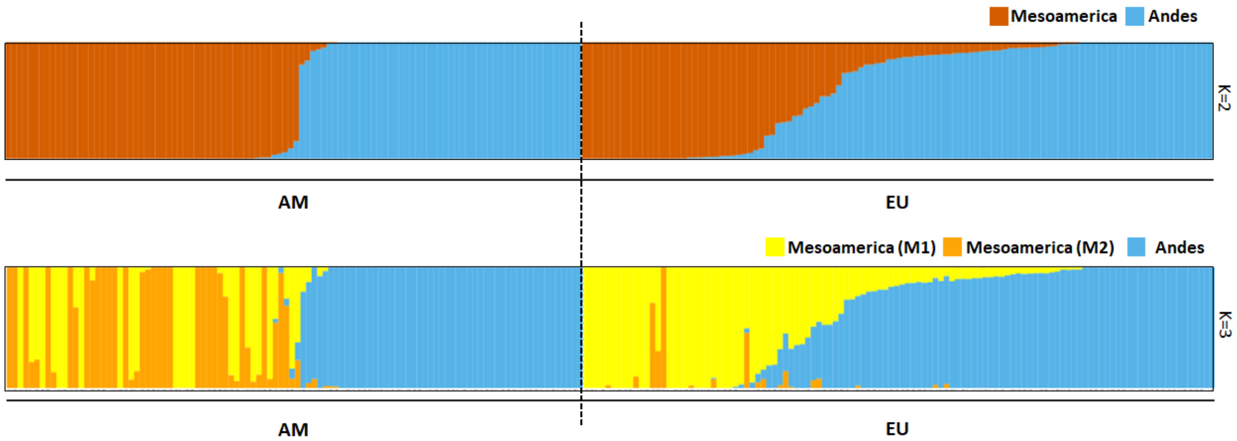

Supplementary Figure 12. Admixture plots showing the inferred ancestry in American (AM) and European (EU) accessions. Runs assuming K=2 and K=3 are shown.

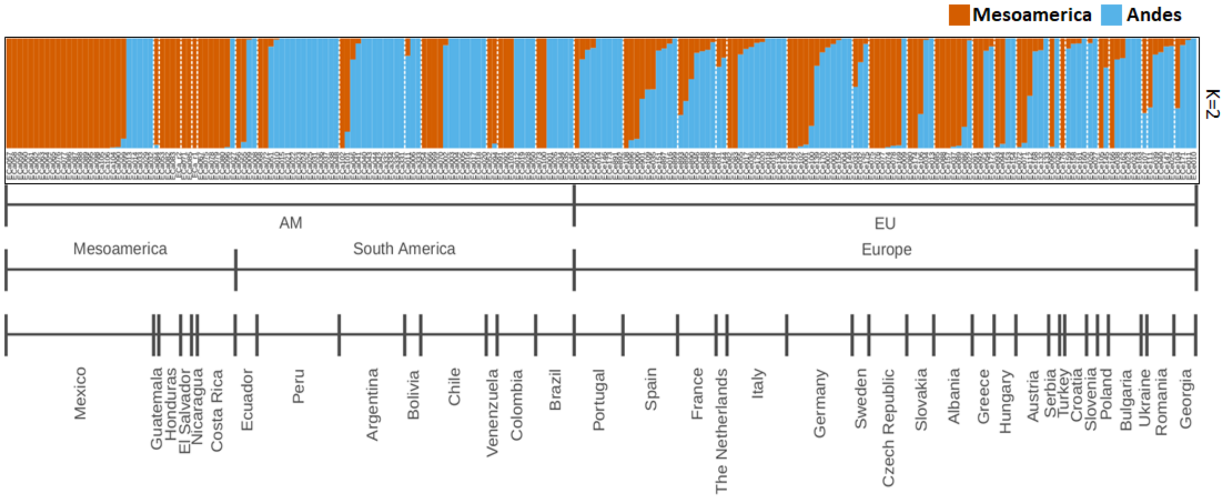

Supplementary Figure 13. Admixture plots showing the inferred ancestry in American (AM) and European (EU) accessions. Accessions are grouped by geographic origin, assuming K=2.

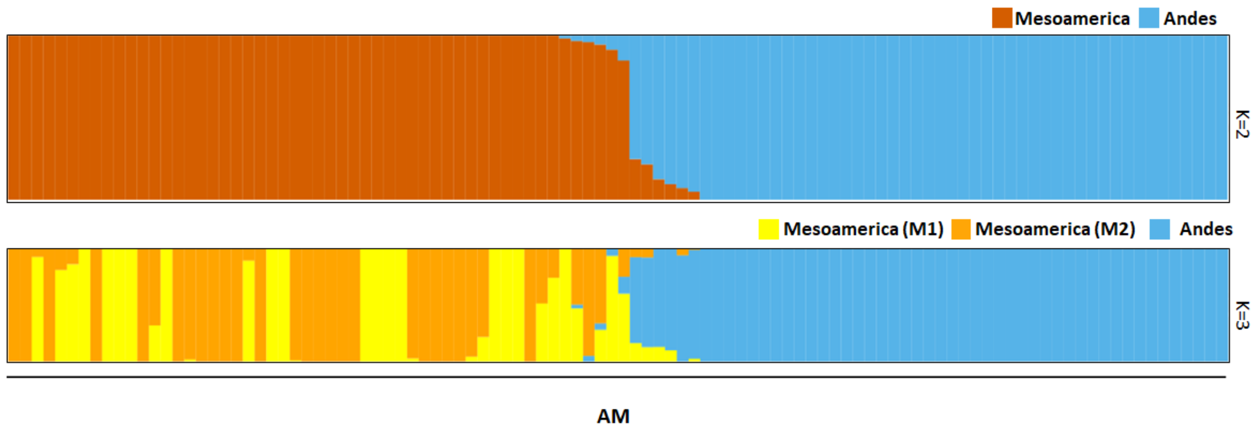

Supplementary Figure 14. Admixture plots showing the inferred ancestry in American (AM) accessions. Runs assuming K=2 and K=3 are shown.

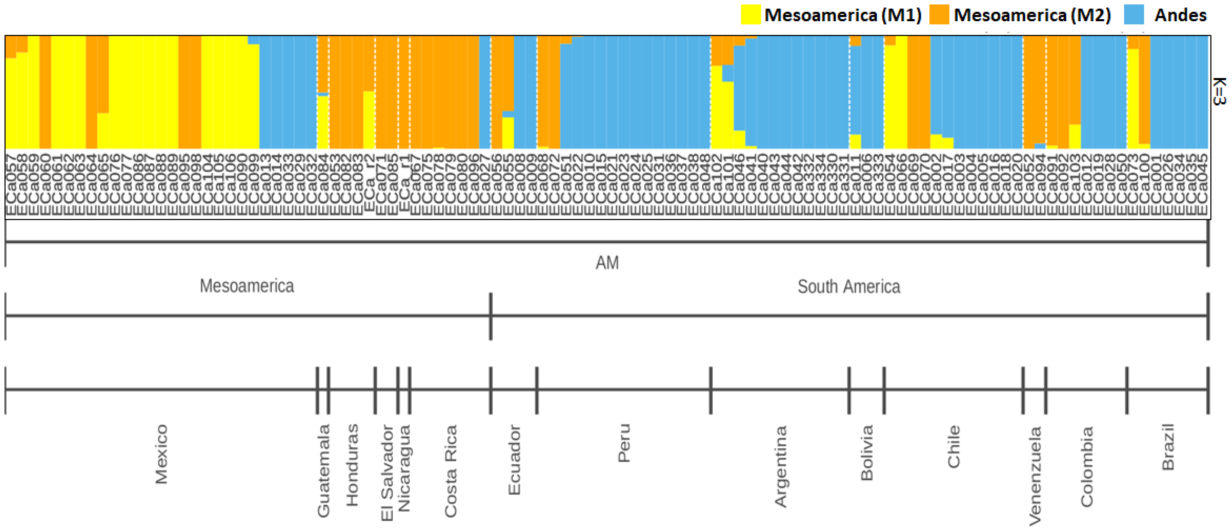

Supplementary Figure 15. Admixture plots showing the inferred ancestry in American (AM) accessions. Accessions are grouped by geographic origin, assuming K=3.

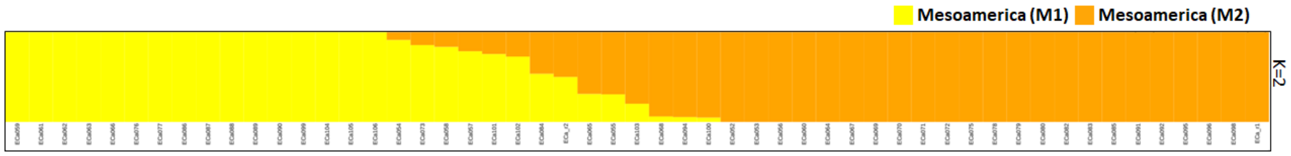

Supplementary Figure 16. Admixture plots showing the inferred ancestry in Mesoamerican accessions assuming K=2.

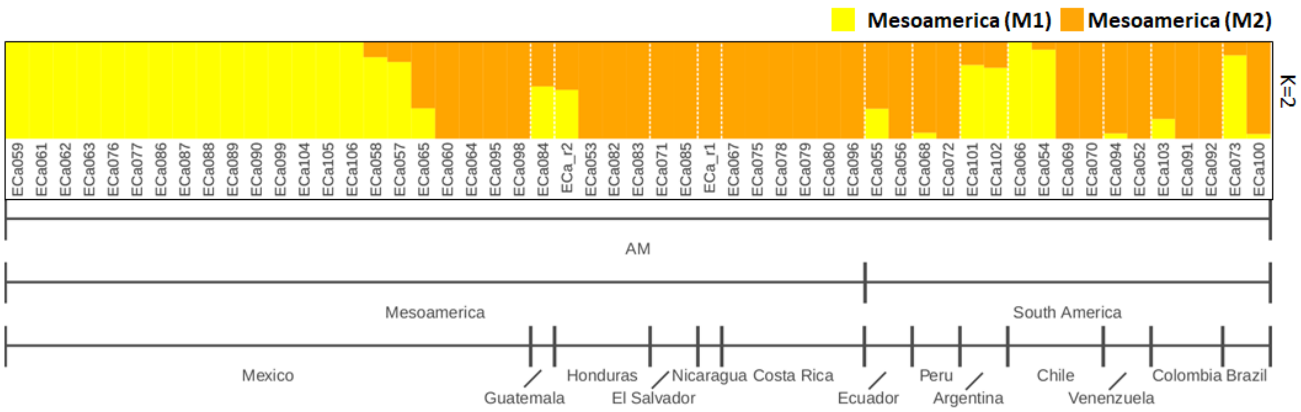

Supplementary Figure 17. Admixture plots showing the inferred ancestry in Mesoamerican accessions. Accessions are grouped by geographic origin, assuming K=2.

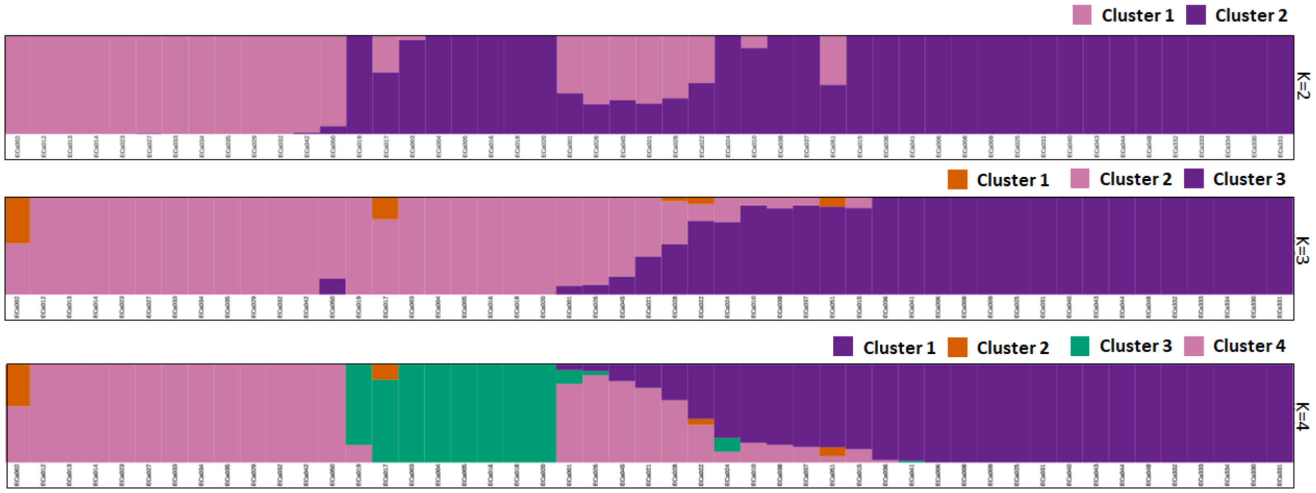

Supplementary Figure 18. Admixture plots showing the inferred ancestry in Andean accessions. Runs assuming K=2, K = 3 and K=4 are shown.

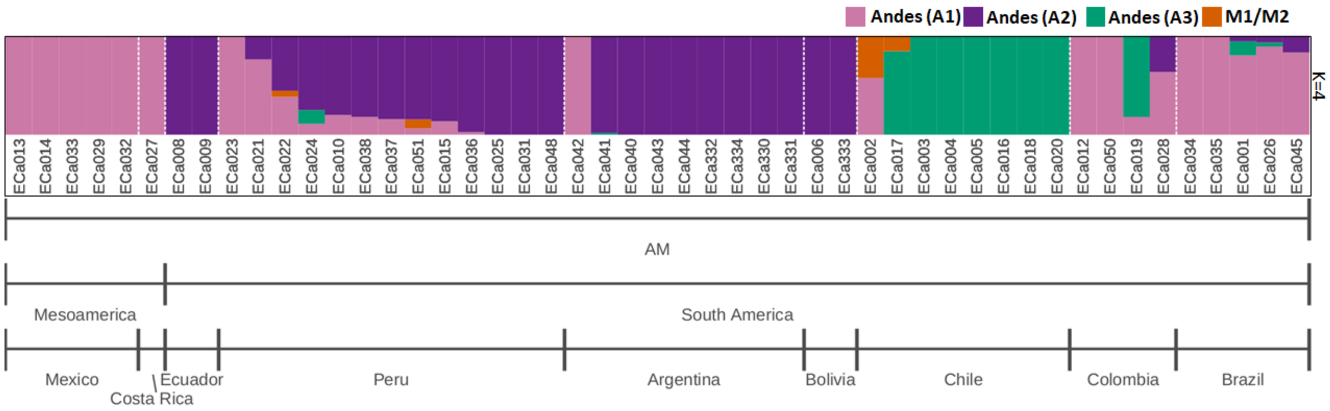

Supplementary Figure 19. Admixture plots showing the inferred ancestry in Andean accessions. Accessions are grouped by geographic origin, assuming K=4.

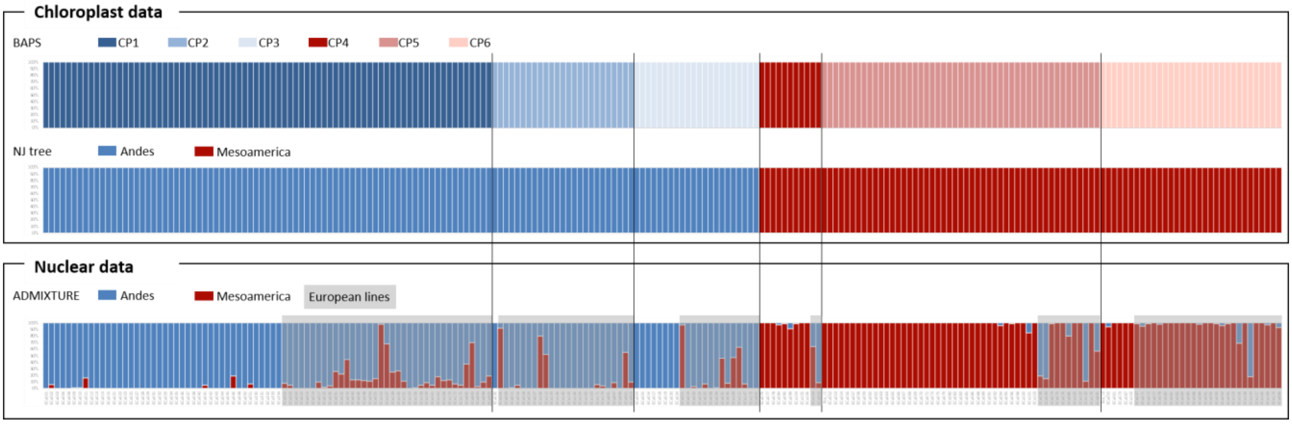

Supplementary Figure 20. Membership of each genotype to BAPS groups CP1, CP2, CP3, CP4, CP5 and CP6. The plots show the membership of each genotype to BAPS groups CP1, CP2, CP3, CP4, CP5 and CP6 and the relative assignment to Andean or Mesoamerican gene pool based on a NJ tree using chloroplast data. The results are compared with the percentages of membership (q) for each of the two clusters (Andes and Mesoamerica) identified by Admixture analysis by using nuclear data. Each genotype is represented by a vertical line.

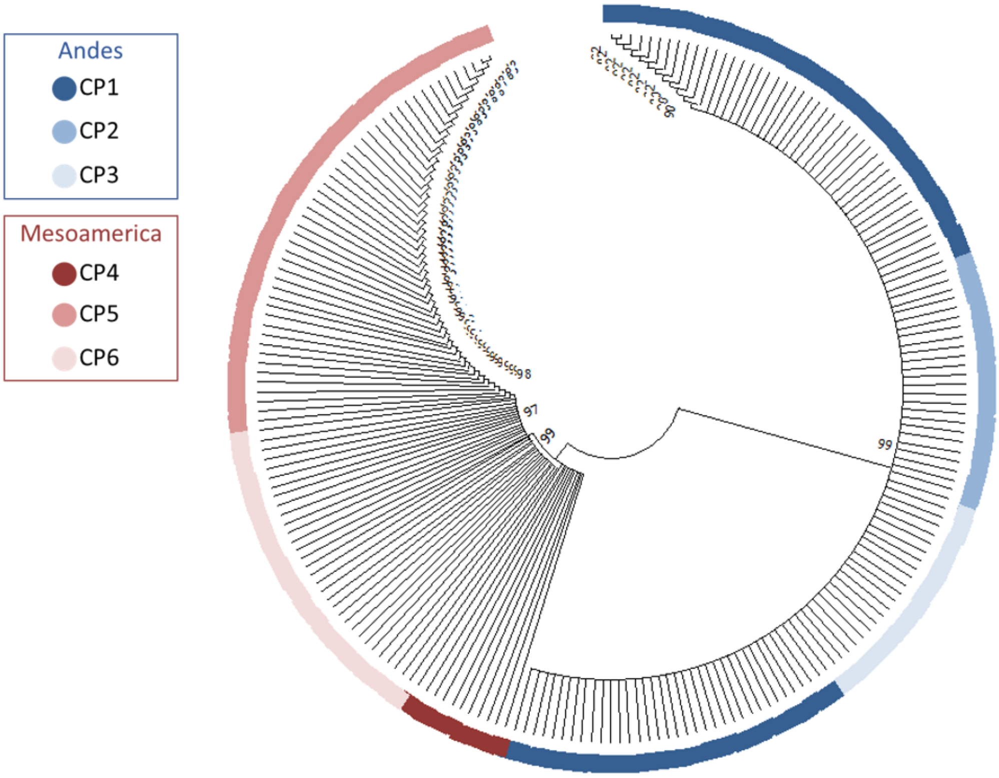

Supplementary Figure 21. Unrooted NJ tree showing the phylogenetic relationships among the different genotypes highlighted for membership to CP1, CP2, CP3, CP4, CP5 and CP6 BAPS groups.

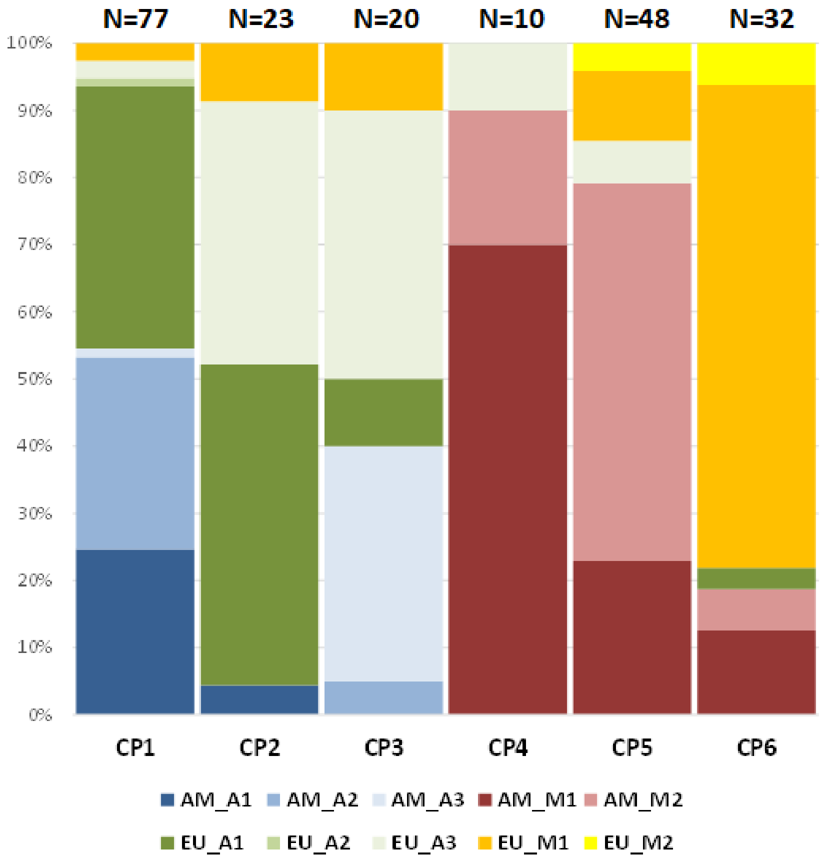

Supplementary Figure 22. Association between genetic groups identified by analysing chloroplast and nuclear data.

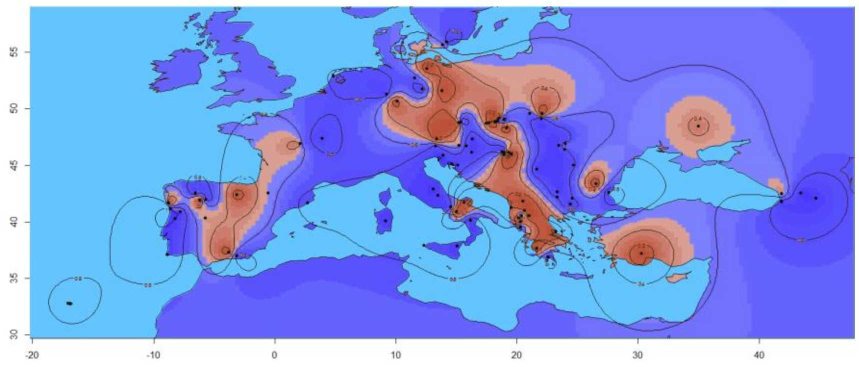

Supplementary Figure 23. Spatial interpolation of the geographic distribution of the Mesoamerican (red) and the Andean (blue) ancestry component in Europe, inferred by ADMIXTURE (K=2). Maps were designed using the map tools implemented in different R packages, such as *spatial*, *maps*, *fields*, *maptools*, *raster*, *rgdal*.

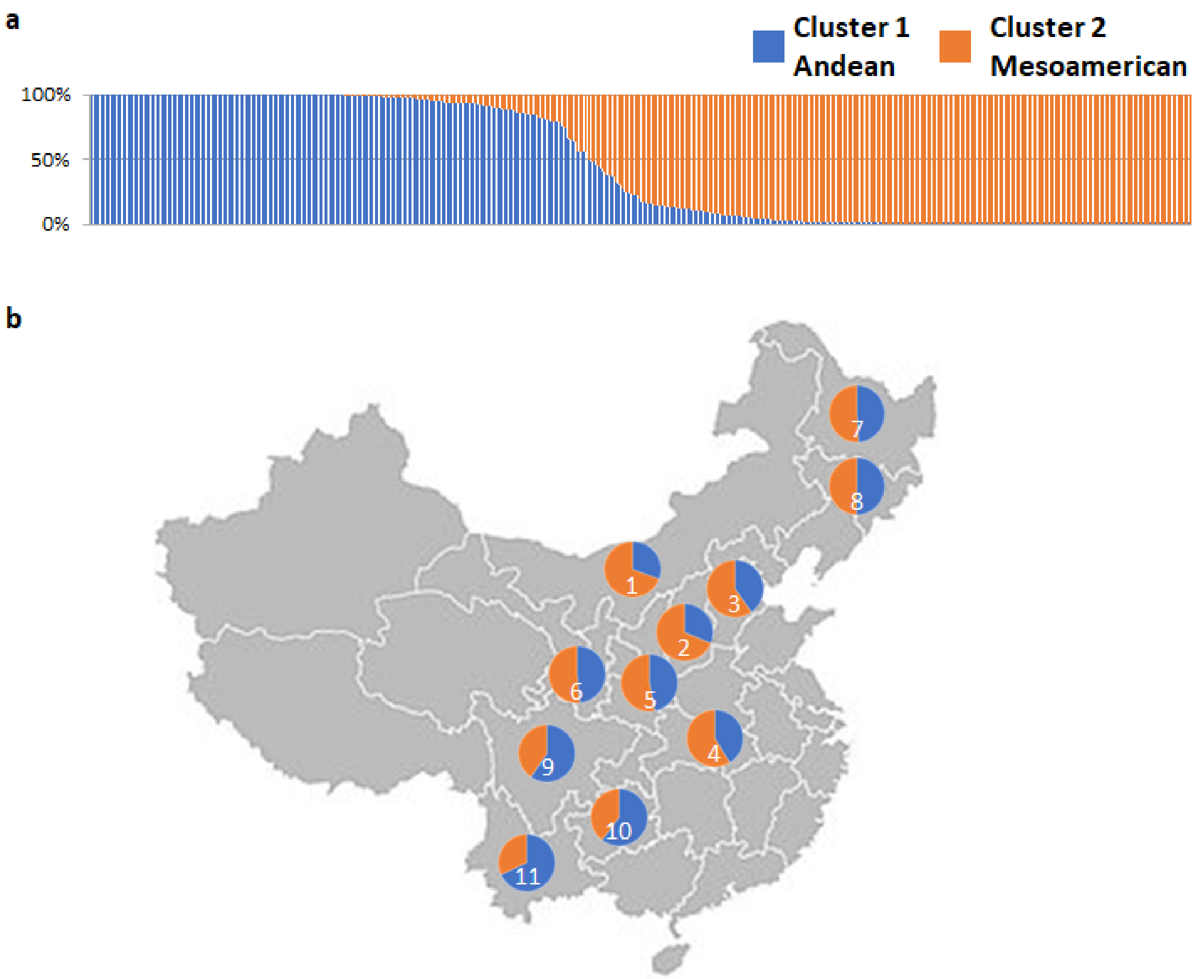

Supplementary Figure 24. Admixture analysis (K=2) on 457 Chinese landraces from the work of Wu *et al*.[33]. a) Result of the admixture analysis (K=2) by accessions, b) pie-chart showing the average percentage of the two ancestries (as identified by the K=2 adxmiture analysis) across each Chinese provinces for the landraces from Wu *et al*.[33]; only provinces with at least ten landraces have been reported in the map. Chinese provinces are numbered in (b) as following: China-Inner Mongolia, 1; Shanxi, 2; Hebei, 3; Hubei, 4; Shaanxi, 5; Gansu, 6; Heilongjiang, 7; Jilin, 8; Sichuan, 9; Guizhou, 10; Yunnan, 11. Blue; Cluster 1 (corresponding to the Andean gene pool), red; Cluster 2 (corresponding to the Mesoamerican gene pool). Source data are provided as a Source Data file.

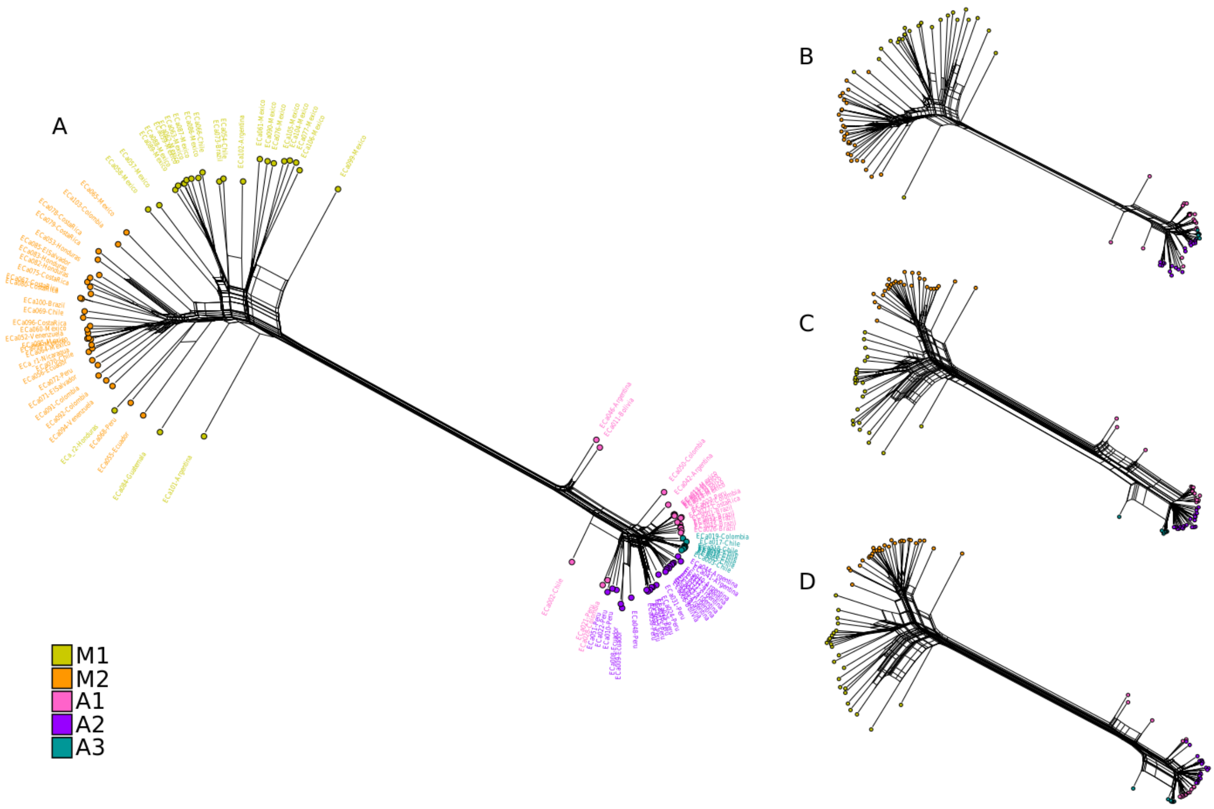

Supplementary Figure 25. Phylogenetic networks generated by SPLITTREES over American accessions. Networks were created using all coding (A) and all neutral regions (C). In plot (B) and (D) 3,000 randomly extracted coding or neutral regions were used, respectively. Individuals attributed to the Mesoamerican subgroups M1 and M2 are colored in yellow and orange. Individuals assigned to the Andean subgroups A1, A2 and A3 are reported in pink, purple and green.

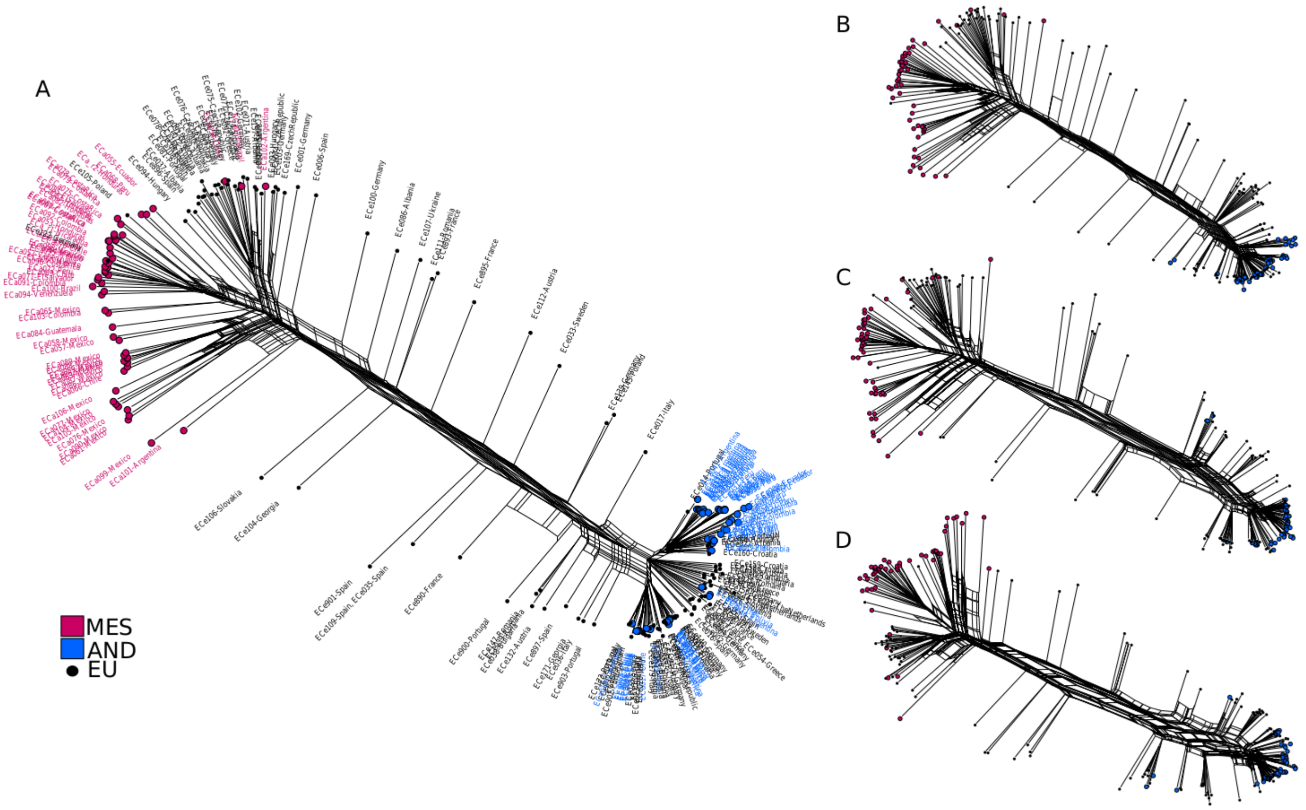

Supplementary Figure 26. Phylogenetic networks generated by SPLITTREES over American and European accessions. Networks were created using all the coding (A) an all the neutral regions (C). In plot (B) and (D) 3,000 randomly extracted coding or neutral regions were used. Individuals attributed to the Mesoamerican and Andean gene pools are colored in red and blue respectively. European individuals are indicated as black circles.

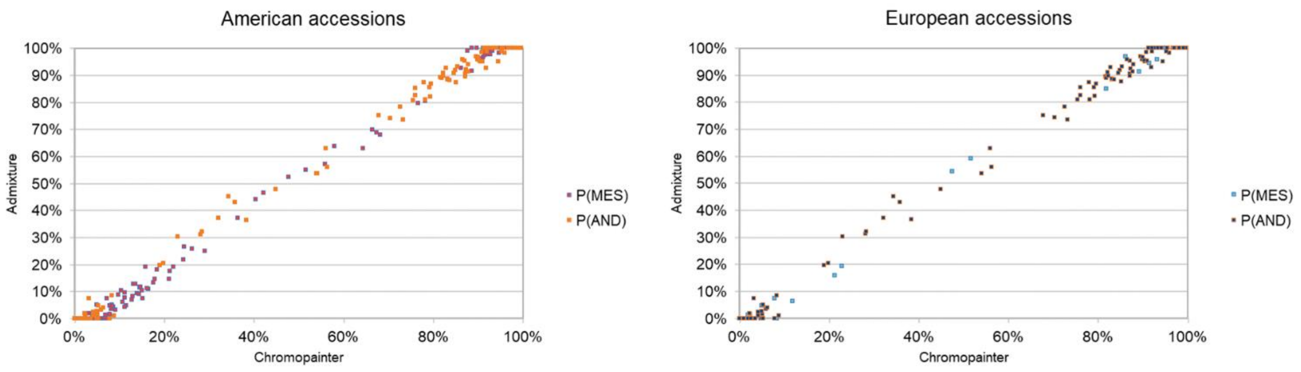

Supplementary Figure 27. Comparison of the estimated proportion of Mesoamerican (P(MES)) and Andean (P(AND)) components in the American (left) and European (right) accessions, using ChromoPainter and Admixture.

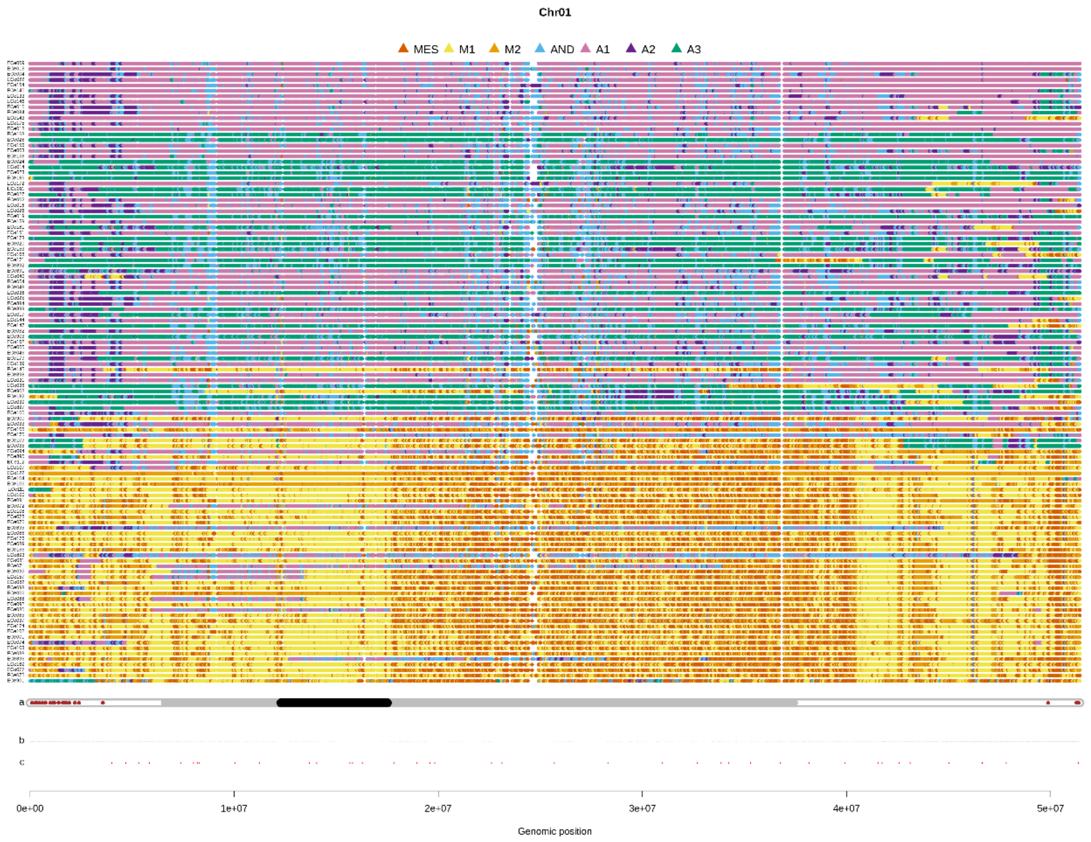

Supplementary Figure 28. Ancestry inference at the chromosomal level using ChromoPainter for the chromosome Chr01. The chromosome was divided in 10kb windows and each chunk was assigned to seven genetic groups, as highlighted with different colors in the legend (i.e., the five genetic groups M1, M2, A1, A2, A3 or the attribution to a Mesoamerican, MES or Andean, AND window, when a specific assignment to a genetic group was not supported; see Supplementary Note 19 for details). Individuals are ordered according to their estimated total proportion of Andean and Mesoamerican components. The chromosome structure (a), including the putative centromeric region (black), the pericentromeric region (gray) and the subtelomeric regions (brown), are reported below the plot. The density of genes (b) and the location of simple repeats (c) longer than 500bp are also reported.

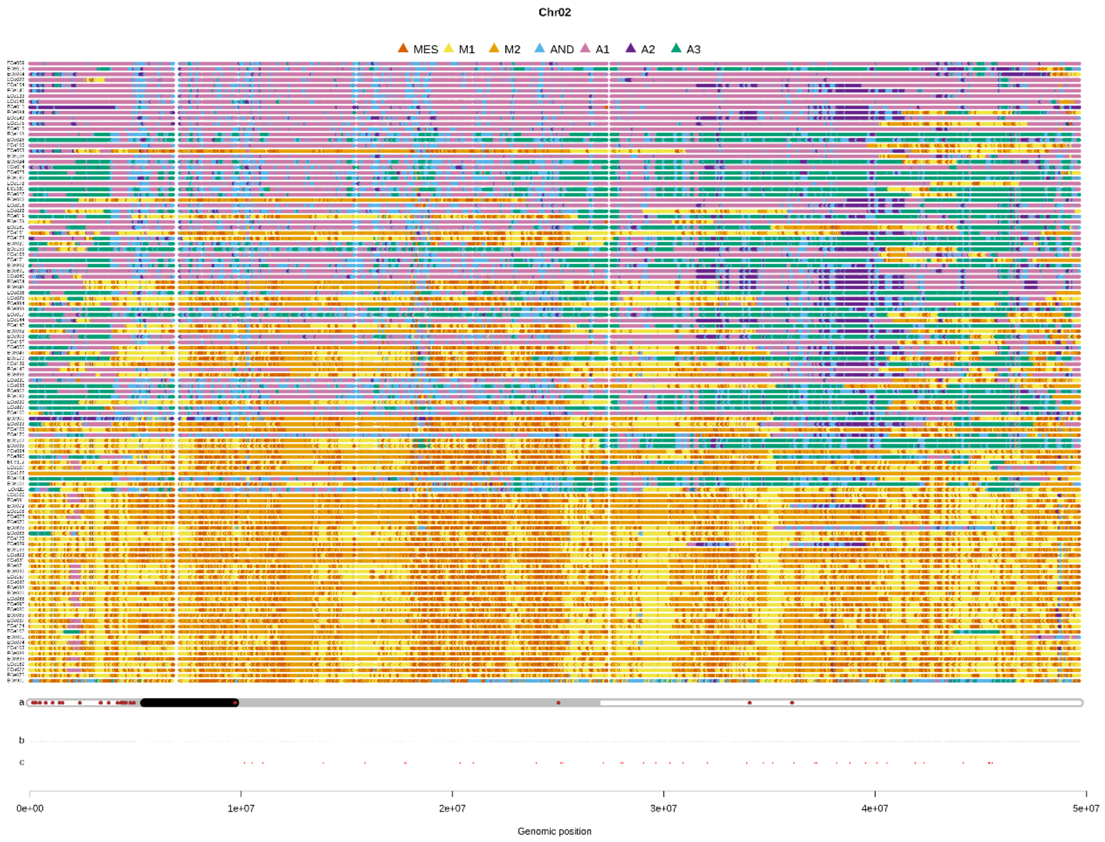

Supplementary Figure 29. Ancestry inference at the chromosomal level using ChromoPainter for the chromosome Chr02. The chromosome was divided in 10kb windows and each chunk was assigned to seven genetic groups, as highlighted with different colors in the legend (i.e., the five genetic groups M1, M2, A1, A2, A3 or the attribution to a Mesoamerican, MES or Andean, AND window, when a specific assignment to a genetic group was not supported; see Supplementary Note 19 for details). Individuals are ordered according to their estimated total proportion of Andean and Mesoamerican components. The chromosome structure (a), including the putative centromeric region (black), the pericentromeric region (gray) and the subtelomeric regions (brown), are reported below the plot. The density of genes (b) and the location of simple repeats (c) longer than 500bp are also reported.

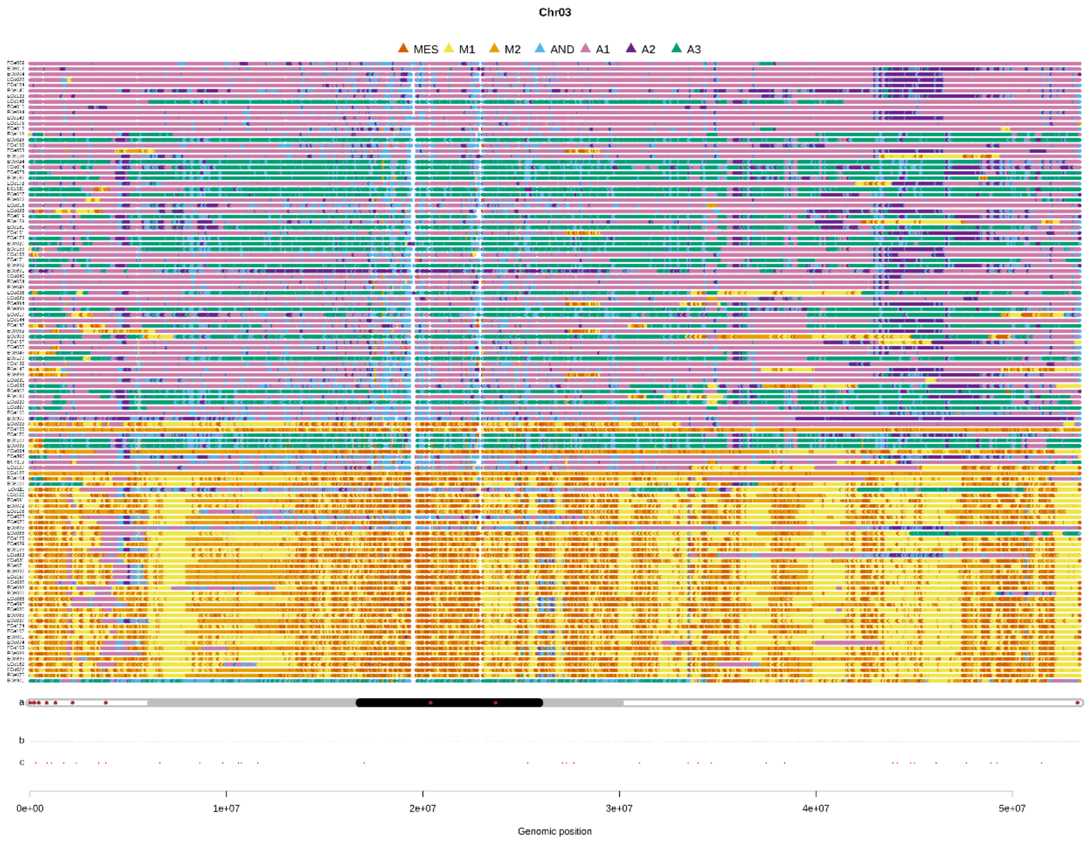

Supplementary Figure 30. Ancestry inference at the chromosomal level using ChromoPainter for the chromosome Chr03. The chromosome was divided in 10kb windows and each chunk was assigned to seven genetic groups, as highlighted with different colors in the legend (i.e., the five genetic groups M1, M2, A1, A2, A3 or the attribution to a Mesoamerican, MES or Andean, AND window, when a specific assignment to a genetic group was not supported; see Supplementary Note 19 for details). Individuals are ordered according to their estimated total proportion of Andean and Mesoamerican components. The chromosome structure (a), including the putative centromeric region (black), the pericentromeric region (gray) and the subtelomeric regions (brown), are reported below the plot. The density of genes (b) and the location of simple repeats (c) longer than 500bp are also reported.

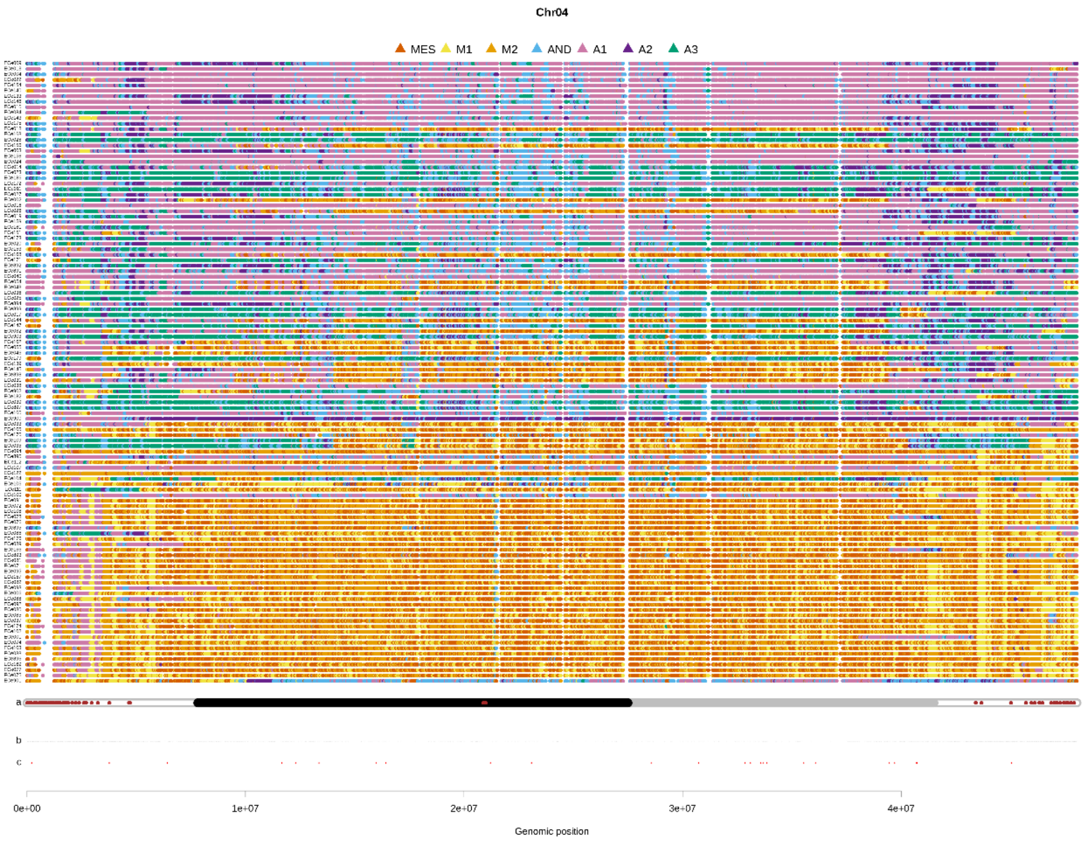

Supplementary Figure 31. Ancestry inference at the chromosomal level using ChromoPainter for the chromosome Chr04. The chromosome was divided in 10kb windows and each chunk was assigned to seven genetic groups, as highlighted with different colors in the legend (i.e., the five genetic groups M1, M2, A1, A2, A3 or the attribution to a Mesoamerican, MES or Andean, AND window, when a specific assignment to a genetic group was not supported; see Supplementary Note 19 for details). Individuals are ordered according to their estimated total proportion of Andean and Mesoamerican components. The chromosome structure (a), including the putative centromeric region (black), the pericentromeric region (gray) and the subtelomeric regions (brown), are reported below the plot. The density of genes (b) and the location of simple repeats (c) longer than 500bp are also reported.

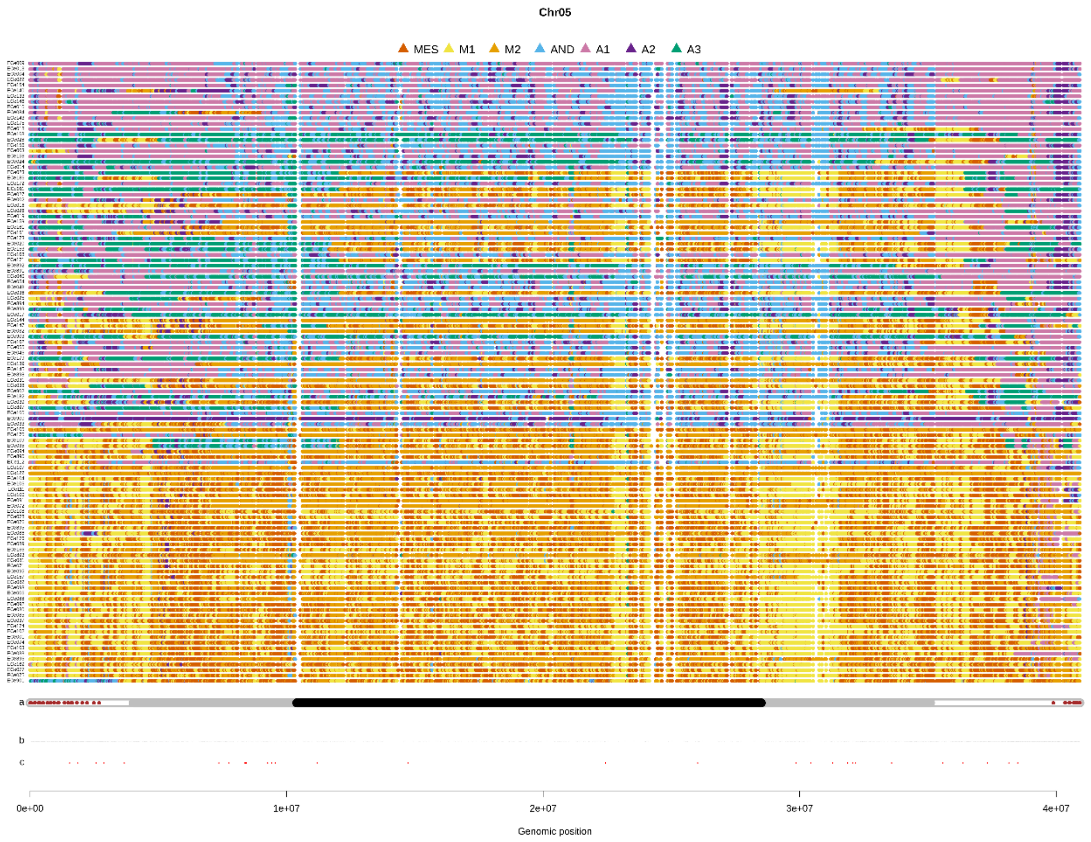

Supplementary Figure 32. Ancestry inference at the chromosomal level using ChromoPainter for the chromosome Chr05. The chromosome was divided in 10kb windows and each chunk was assigned to seven genetic groups, as highlighted with different colors in the legend (i.e., the five genetic groups M1, M2, A1, A2, A3 or the attribution to a Mesoamerican, MES or Andean, AND window, when a specific assignment to a genetic group was not supported; see Supplementary Note 19 for details). Individuals are ordered according to their estimated total proportion of Andean and Mesoamerican components. The chromosome structure (a), including the putative centromeric region (black), the pericentromeric region (gray) and the subtelomeric regions (brown), are reported below the plot. The density of genes (b) and the location of simple repeats (c) longer than 500bp are also reported.

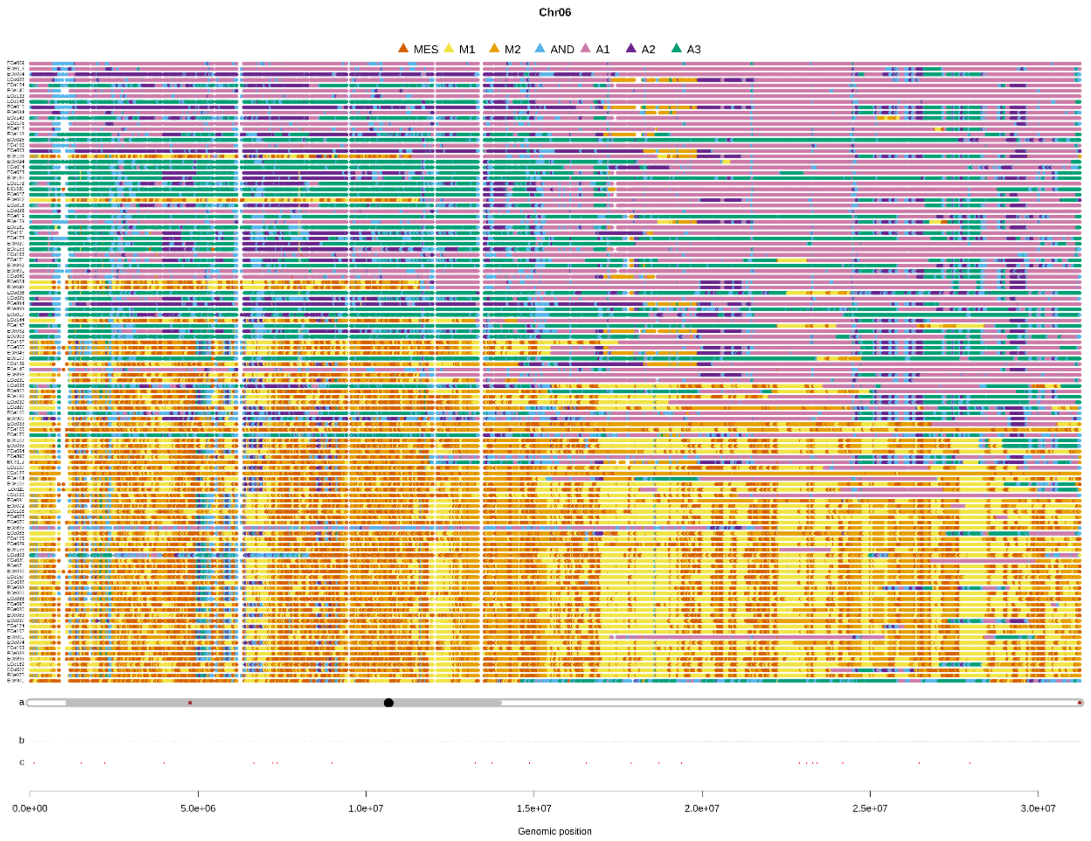

Supplementary Figure 33. Ancestry inference at the chromosomal level using ChromoPainter for the chromosome Chr06. The chromosome was divided in 10kb windows and each chunk was assigned to seven genetic groups, as highlighted with different colors in the legend (i.e., the five genetic groups M1, M2, A1, A2, A3 or the attribution to a Mesoamerican, MES or Andean, AND window, when a specific assignment to a genetic group was not supported; see Supplementary Note 19 for details). Individuals are ordered according to their estimated total proportion of Andean and Mesoamerican components. The chromosome structure (a), including the putative centromeric region (black), the pericentromeric region (gray) and the subtelomeric regions (brown), are reported below the plot. The density of genes (b) and the location of simple repeats (c) longer than 500bp are also reported.

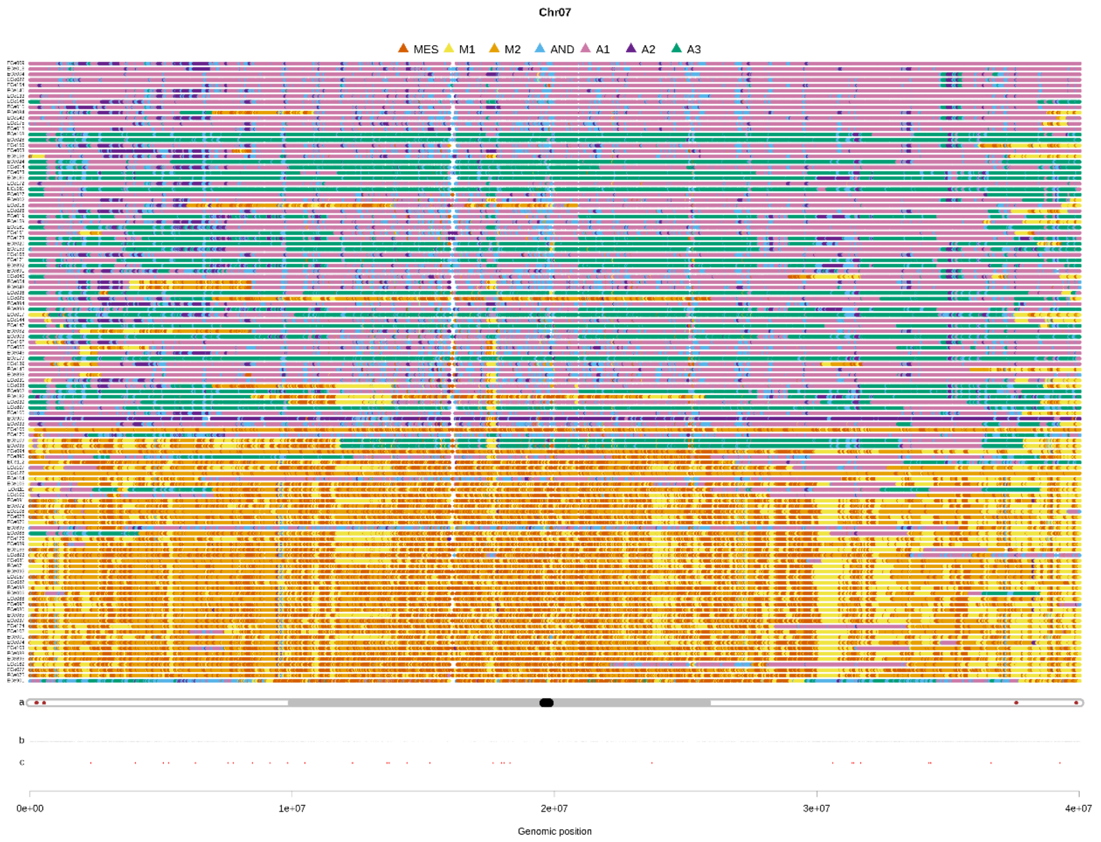

Supplementary Figure 34. Ancestry inference at the chromosomal level using ChromoPainter for the chromosome Chr07. The chromosome was divided in 10kb windows and each chunk was assigned to seven genetic groups, as highlighted with different colors in the legend (i.e., the five genetic groups M1, M2, A1, A2, A3 or the attribution to a Mesoamerican, MES or Andean, AND window, when a specific assignment to a genetic group was not supported; see Supplementary Note 19 for details). Individuals are ordered according to their estimated total proportion of Andean and Mesoamerican components. The chromosome structure (a), including the putative centromeric region (black), the pericentromeric region (gray) and the subtelomeric regions (brown), are reported below the plot. The density of genes (b) and the location of simple repeats (c) longer than 500bp are also reported.

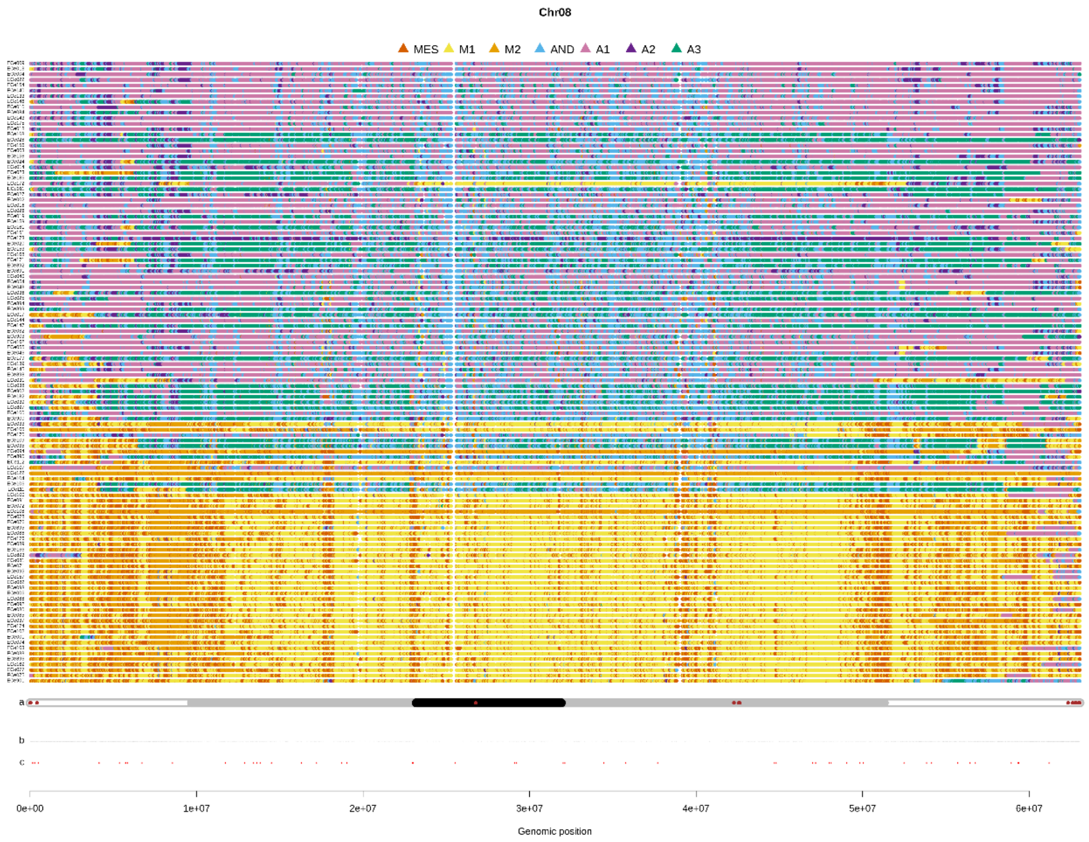

Supplementary Figure 35. Ancestry inference at the chromosomal level using ChromoPainter for the chromosome Chr08. The chromosome was divided in 10kb windows and each chunk was assigned to seven genetic groups, as highlighted with different colors in the legend (i.e., the five genetic groups M1, M2, A1, A2, A3 or the attribution to a Mesoamerican, MES or Andean, AND window, when a specific assignment to a genetic group was not supported; see Supplementary Note 19 for details). Individuals are ordered according to their estimated total proportion of Andean and Mesoamerican components. The chromosome structure (a), including the putative centromeric region (black), the pericentromeric region (gray) and the subtelomeric regions (brown), are reported below the plot. The density of genes (b) and the location of simple repeats (c) longer than 500bp are also reported.

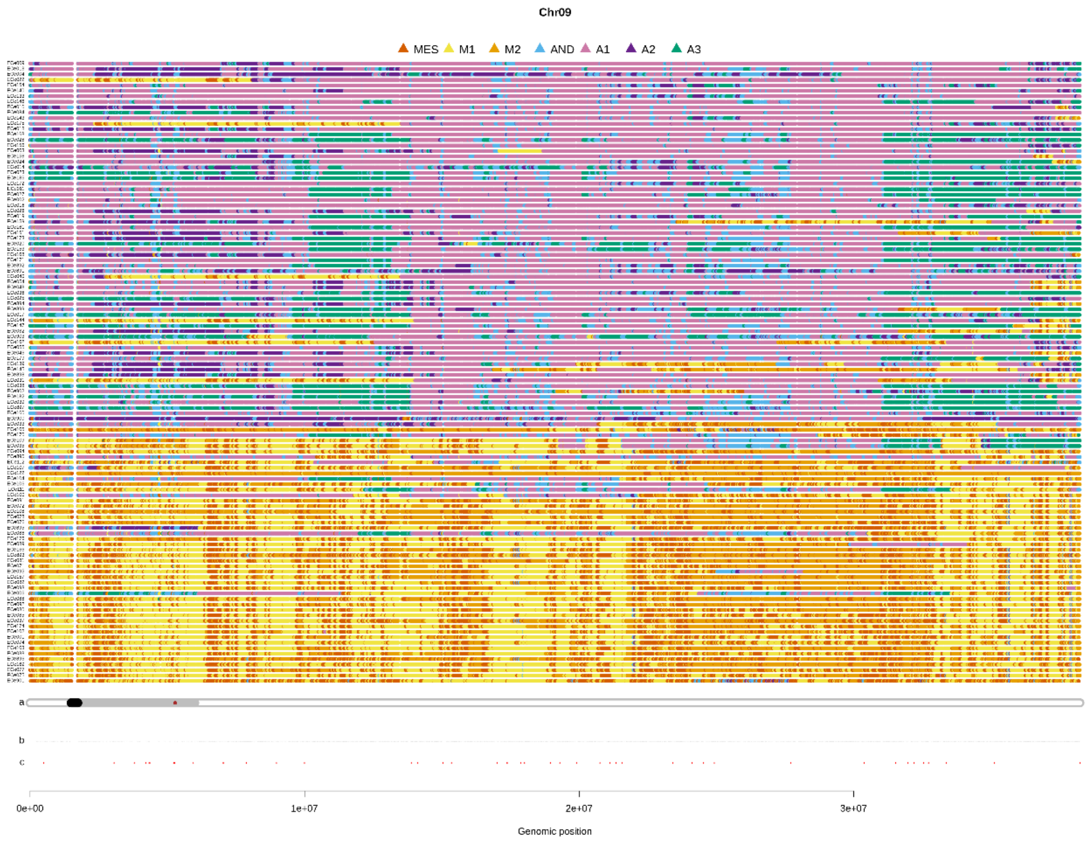

Supplementary Figure 36. Ancestry inference at the chromosomal level using ChromoPainter for the chromosome Chr09. The chromosome was divided in 10kb windows and each chunk was assigned to seven genetic groups, as highlighted with different colors in the legend (i.e., the five genetic groups M1, M2, A1, A2, A3 or the attribution to a Mesoamerican, MES or Andean, AND window, when a specific assignment to a genetic group was not supported; see Supplementary Note 19 for details). Individuals are ordered according to their estimated total proportion of Andean and Mesoamerican components. The chromosome structure (a), including the putative centromeric region (black), the pericentromeric region (gray) and the subtelomeric regions (brown), are reported below the plot. The density of genes (b) and the location of simple repeats (c) longer than 500bp are also reported.

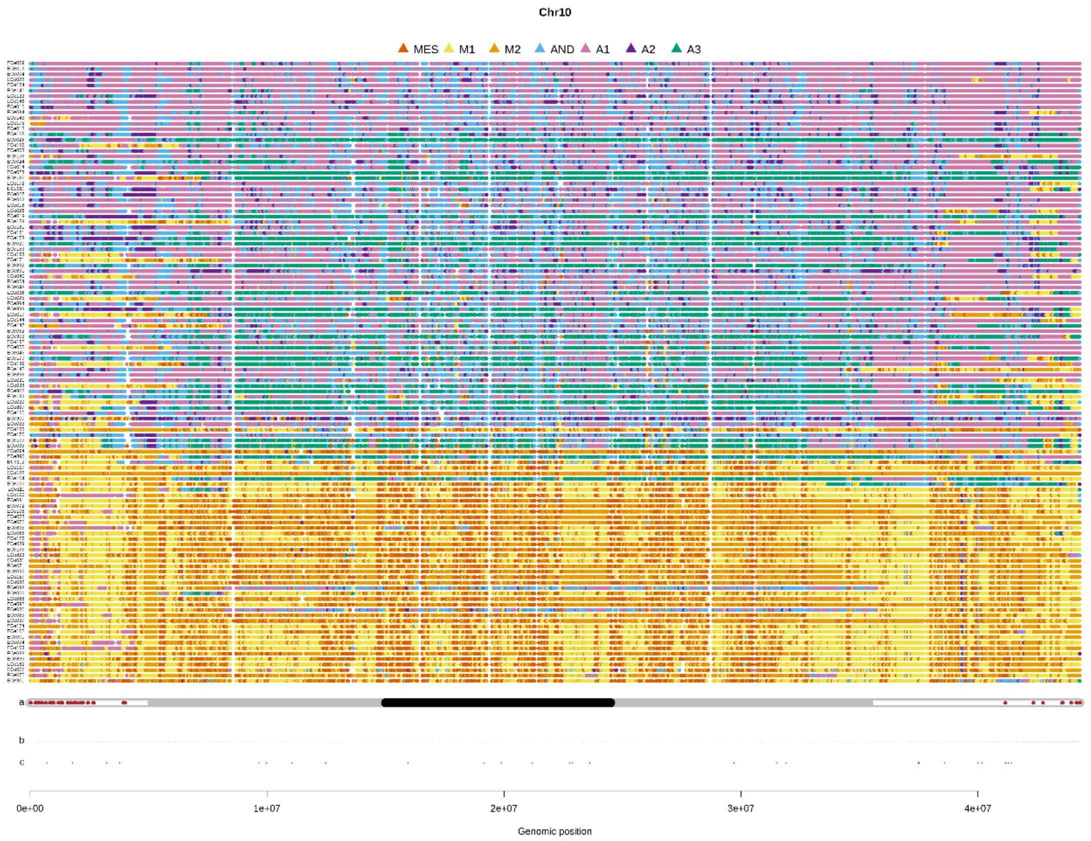

Supplementary Figure 37. Ancestry inference at the chromosomal level using ChromoPainter for the chromosome Chr10. The chromosome was divided in 10kb windows and each chunk was assigned to seven genetic groups, as highlighted with different colors in the legend (i.e., the five genetic groups M1, M2, A1, A2, A3 or the attribution to a Mesoamerican, MES or Andean, AND window, when a specific assignment to a genetic group was not supported; see Supplementary Note 19 for details). Individuals are ordered according to their estimated total proportion of Andean and Mesoamerican components. The chromosome structure (a), including the putative centromeric region (black), the pericentromeric region (gray) and the subtelomeric regions (brown), are reported below the plot. The density of genes (b) and the location of simple repeats (c) longer than 500bp are also reported.

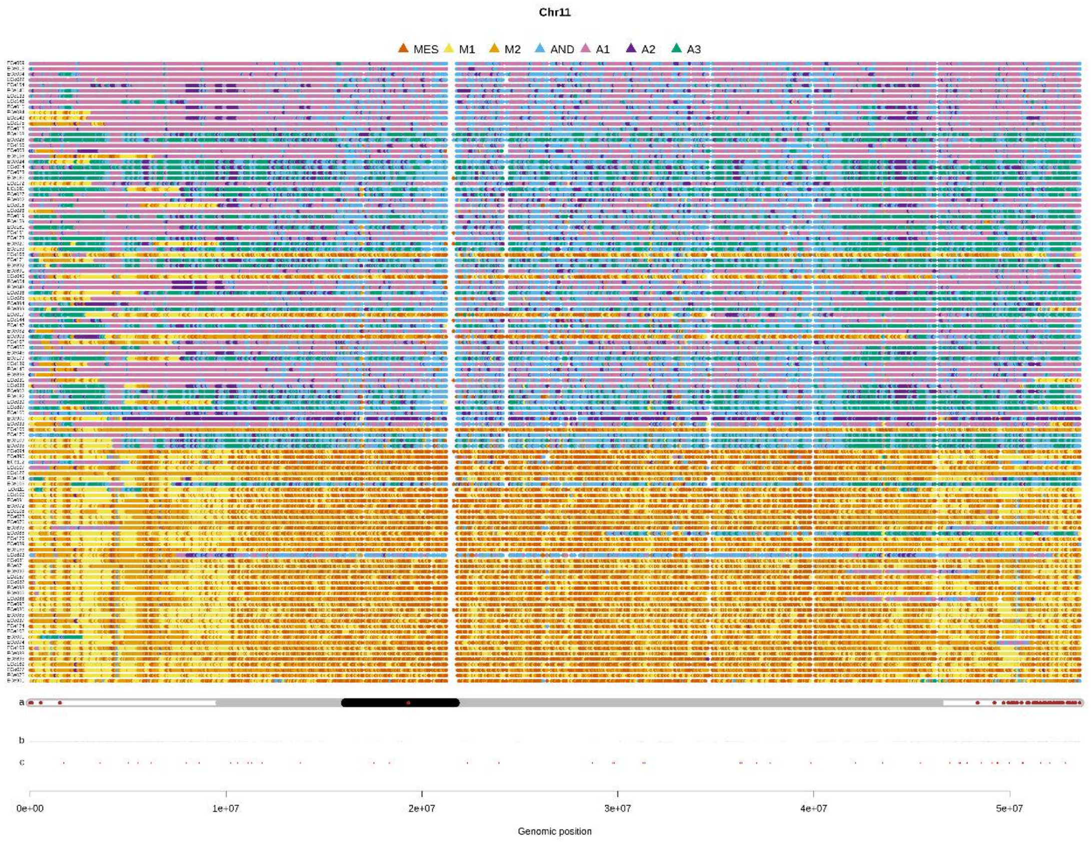

Supplementary Figure 38. Ancestry inference at the chromosomal level using ChromoPainter for the chromosome Chr11. The chromosome was divided in 10kb windows and each chunk was assigned to seven genetic groups, as highlighted with different colors in the legend (i.e., the five genetic groups M1, M2, A1, A2, A3 or the attribution to a Mesoamerican, MES or Andean, AND window, when a specific assignment to a genetic group was not supported; see Supplementary Note 19 for details). Individuals are ordered according to their estimated total proportion of Andean and Mesoamerican components. The chromosome structure (a), including the putative centromeric region (black), the pericentromeric region (gray) and the subtelomeric regions (brown), are reported below the plot. The density of genes (b) and the location of simple repeats (c) longer than 500bp are also reported.

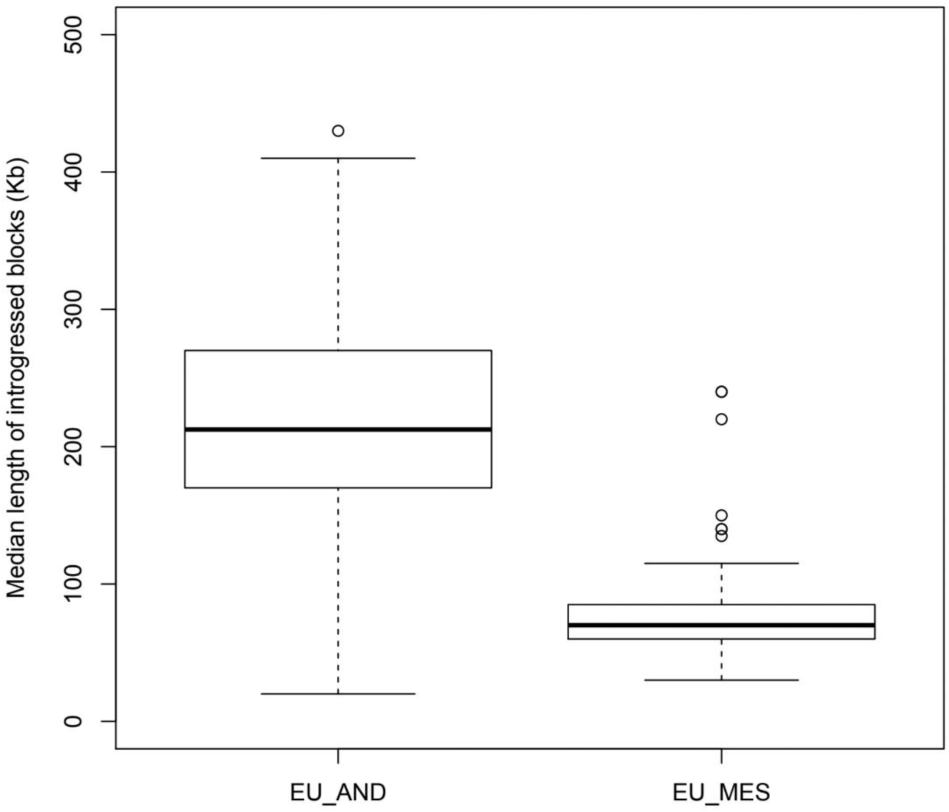

Supplementary Figure 39. Boxplots comparing the median length (kb) of the introgressed blocks identified in each EU_AND and EU_MES individuals excluding individuals with a proportion of introgessed genomes >=40%. Sample size (N. accessions), EU_AND=68, EU_MES=40. Box plots represent minimum, first quartile, median, third quartile and maximum. Source data are provided as a Source Data file.

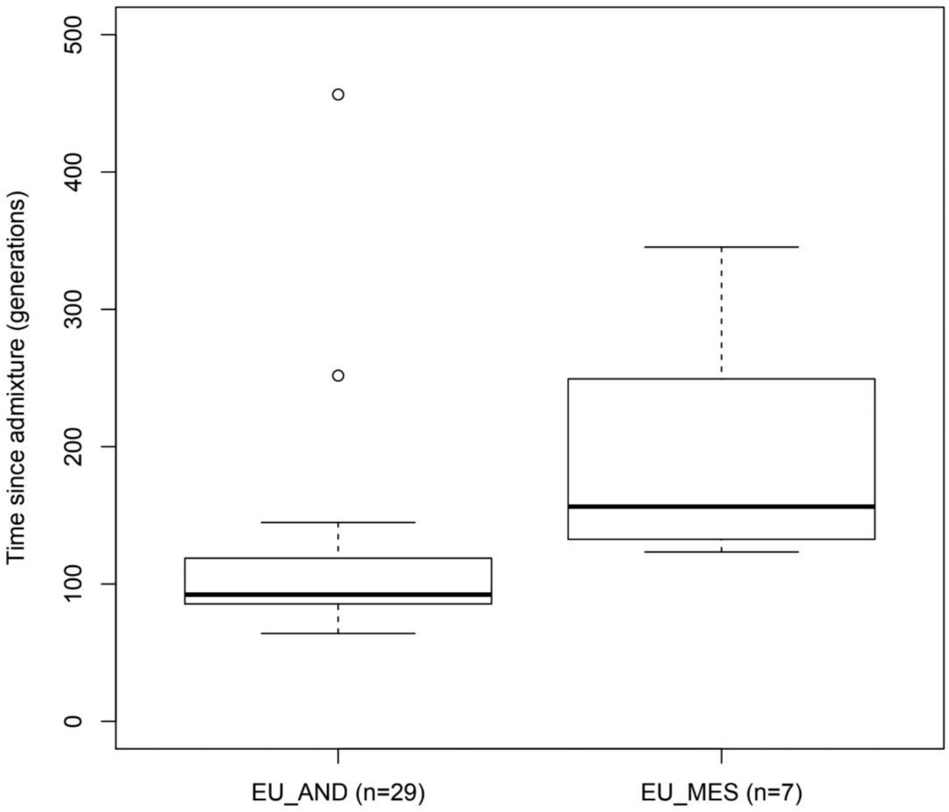

Supplementary Figure 40. Estimated time since admixture in European accessions having >=10% of introgressed genome from the opposite genetic pool. Box plots represent minimum, first quartile, median, third quartile and maximum. Source data are provided as a Source Data file.

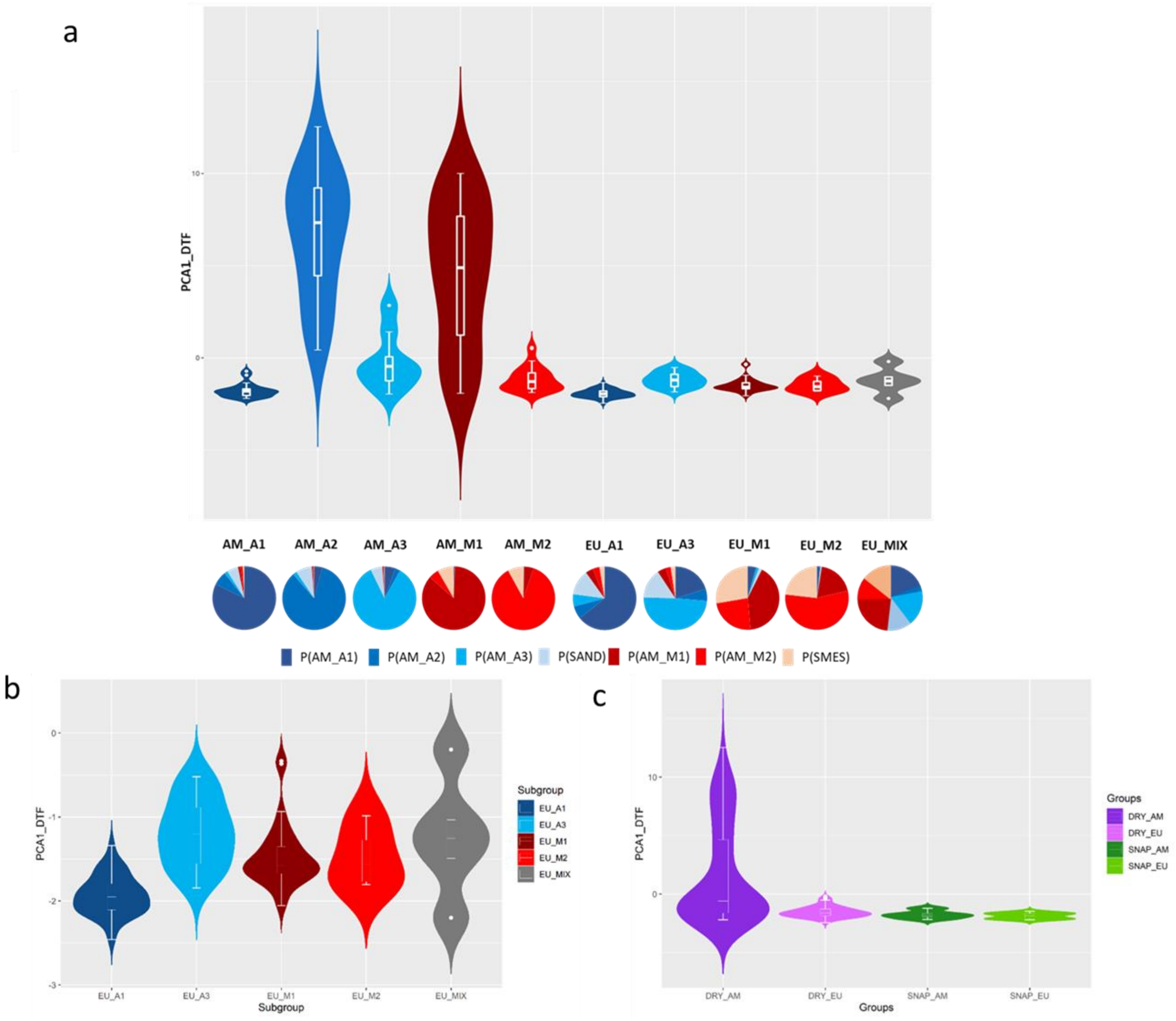

Supplementary Figure 41. Analysis of variance for the PC1 related to the DTF and photoperiod sensitivity (PC1_FLW) by genetic subgroups considering American and European accessions grouped by genetic group (a) (the same as Figure 2d in the main text), only European accessions grouped by genetic group (b) and comparing dry *versus* snap beans for each Continent. The PC1 was used as a representative phenotypic trait for DTF and photoperiod sensitivity and it explains the 68.8% of the total variance for these traits. The proportion of the genetic memberships [i.e., P(AM_A1), P(AM_A2), P(AM_A3), P(AM_M1), and P(AM_M2)] inferred from the donor accessions and composing the American and European accessions (grouped as mainly AM_A1, AM_A2, AM_A3_AM_M1, AM_M2, EU_A1, EU_A3, EU_M1, EU_M2 and EU_MIX) is shown in the pie charts below to the corresponding groups. P(SAND) and P(SMES) are the proportions of the genome that are shared among Andean and Mesoamerican groups, respectively. N. biologically independent samples (a), and (b) for the European (EU) subgroups, AM_A1 (20), AM_A2 (18), AM_A3 (8), AM_M1 (22), AM_M2 (31), EU_A1 (40), EU_A3 (18), EU_M1 (33), EU_M2 (4), EU_MIX (5). N. biologically independent samples (c), DRY_AM (94), DRY_EU (84), SNAP_AM (5), SNAP_EU (16). Box plots represent minimum, first quartile, median, third quartile and maximum. Source data are provided as Source Data file (for Supplementary Figure 41a,b the same as Figure 2d).

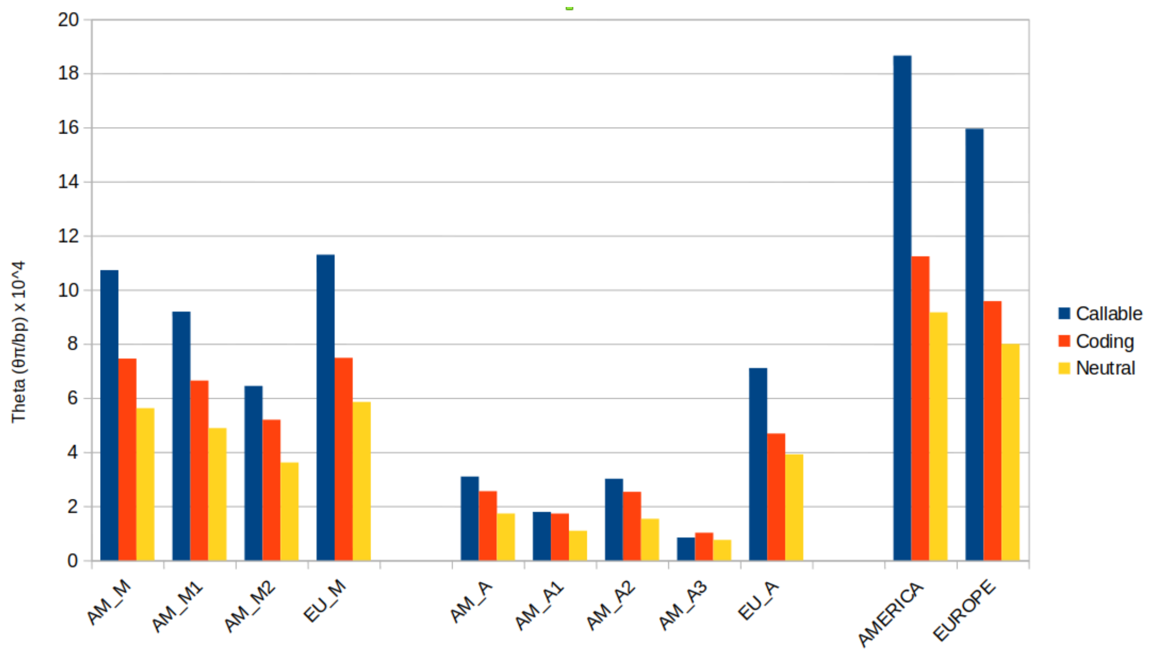

Supplementary Figure 42. Barplot of θ_{π}/bp x 10^4 for each group and genomic partition.

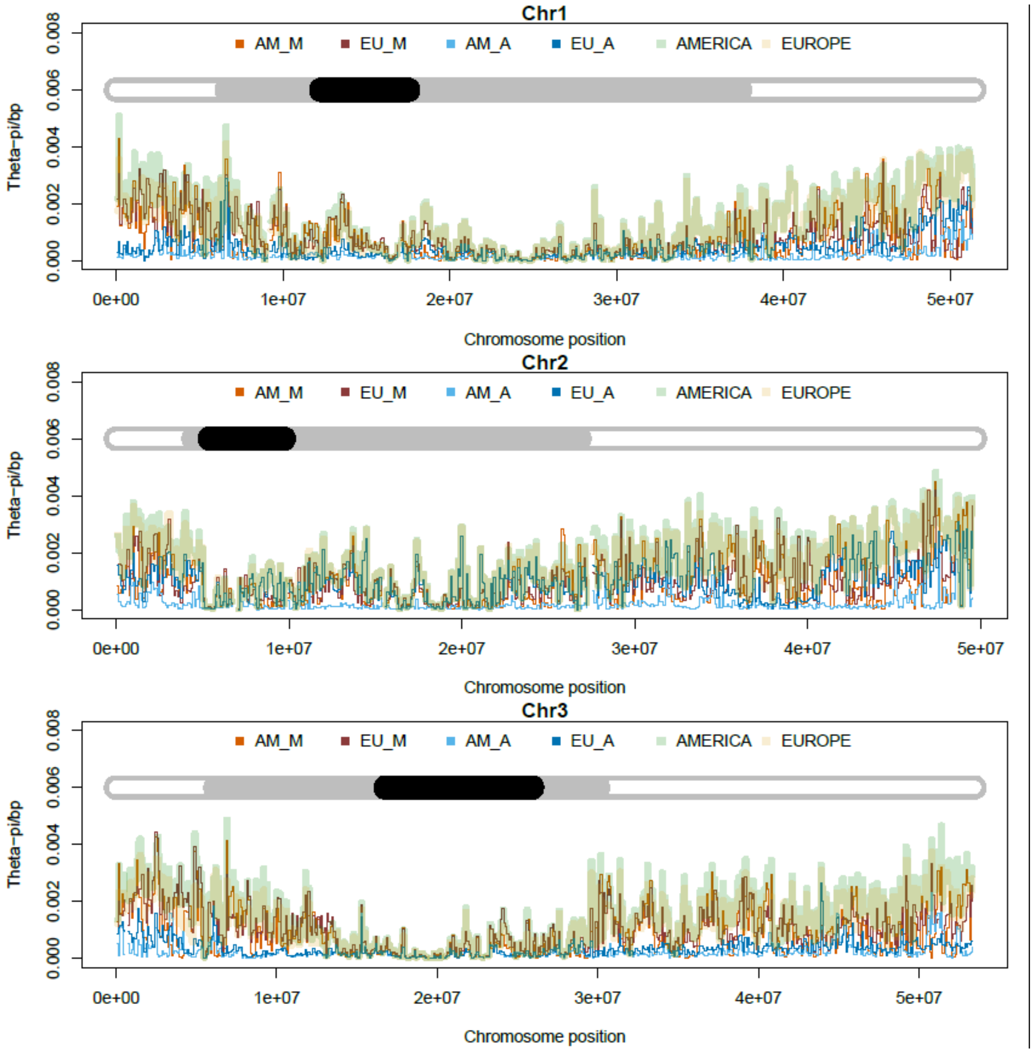

Supplementary Figure 43. Genetic diversity along the Chr01-Chr03 common bean chromosomes. Per-site θ_π was averaged over 100kb not-overlapping sliding windows. Centromeric (black) and pericentromeric (gray) regions for each chromosome are reported. Different colored lines refer to different groups of accessions).

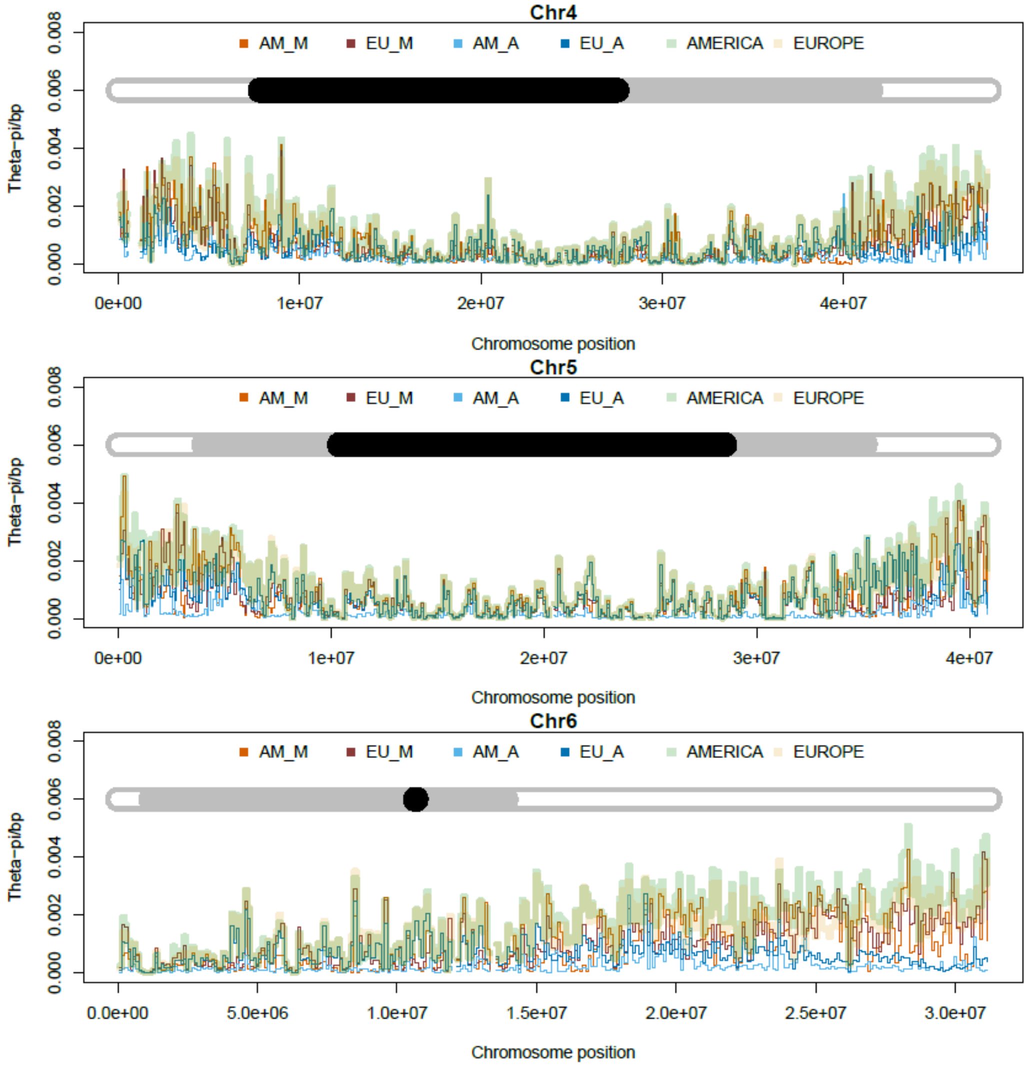

Supplementary Figure 44. Genetic diversity along the Chr04-Chr06 common bean chromosomes. Per-site θ_π was averaged over 100kb not-overlapping sliding windows. Centromeric (black) and pericentromeric (gray) regions for each chromosome are reported. Different colored lines refer to different groups of accessions).

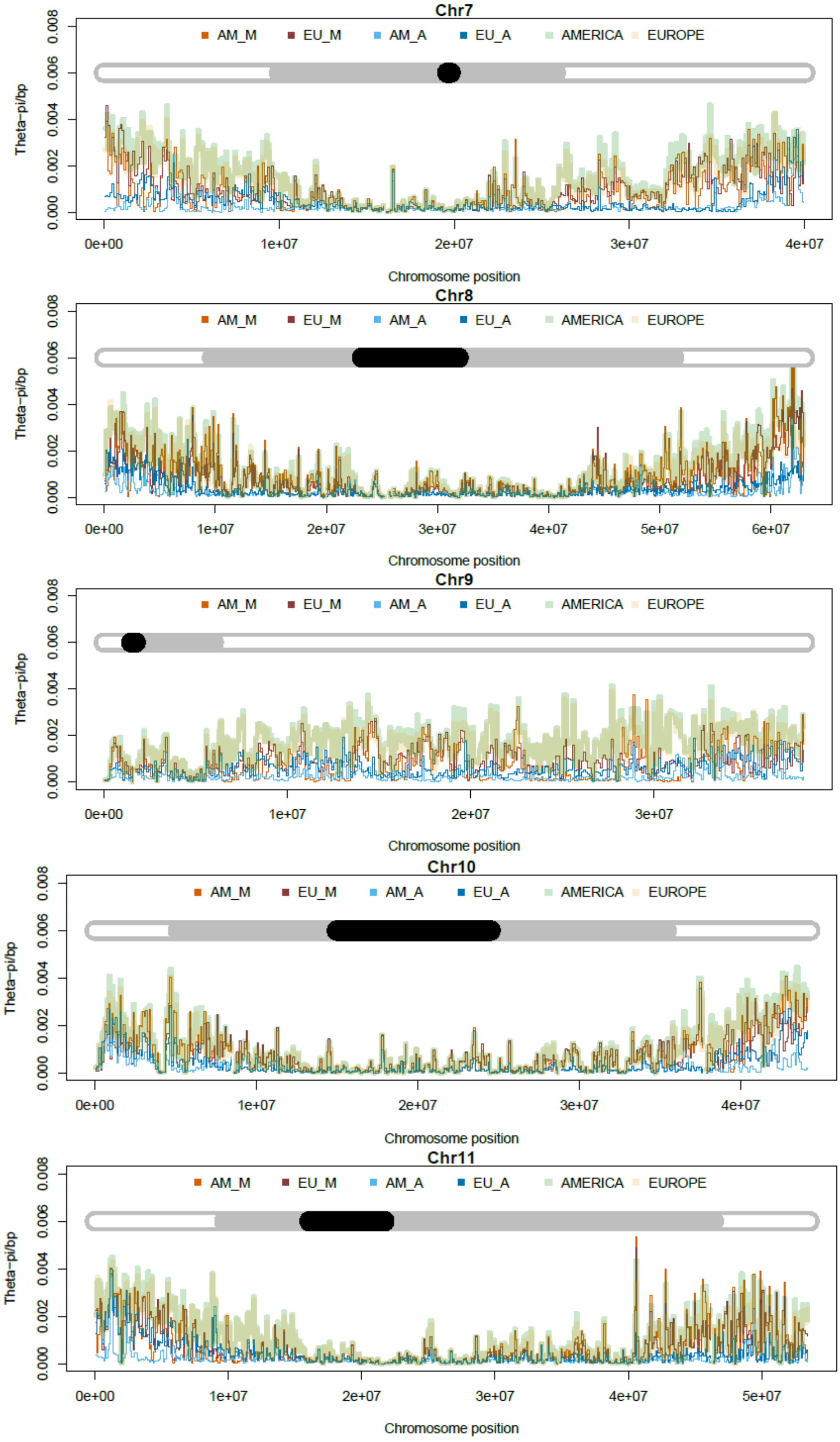

Supplementary Figure 45. Genetic diversity along the Chr07-Chr11 common bean chromosomes. Per-site θ_{π} was averaged over 100kb not-overlapping sliding windows. Centromeric (black) and pericentromeric (gray) regions for each chromosome are reported. Different colored lines refer to different groups of accessions).

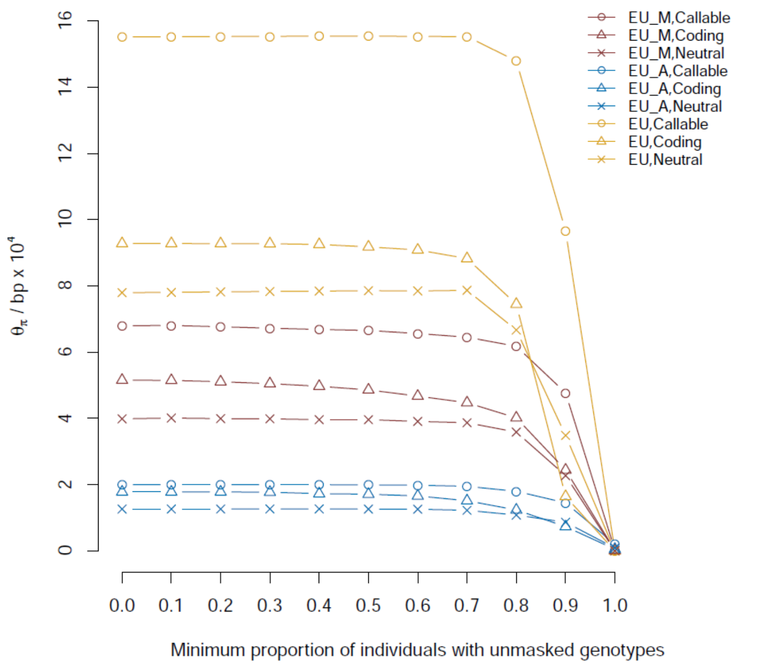

Supplementary Figure 46. The estimated within group θ_π using different proportion of not-masked individuals, in callable, coding and neutral regions. The X axis report the minimum proportion of not-masked individuals that each region should have to be included in the analysis, ranging from 0 (all regions contributing) to 1 (only regions without any masked individuals).

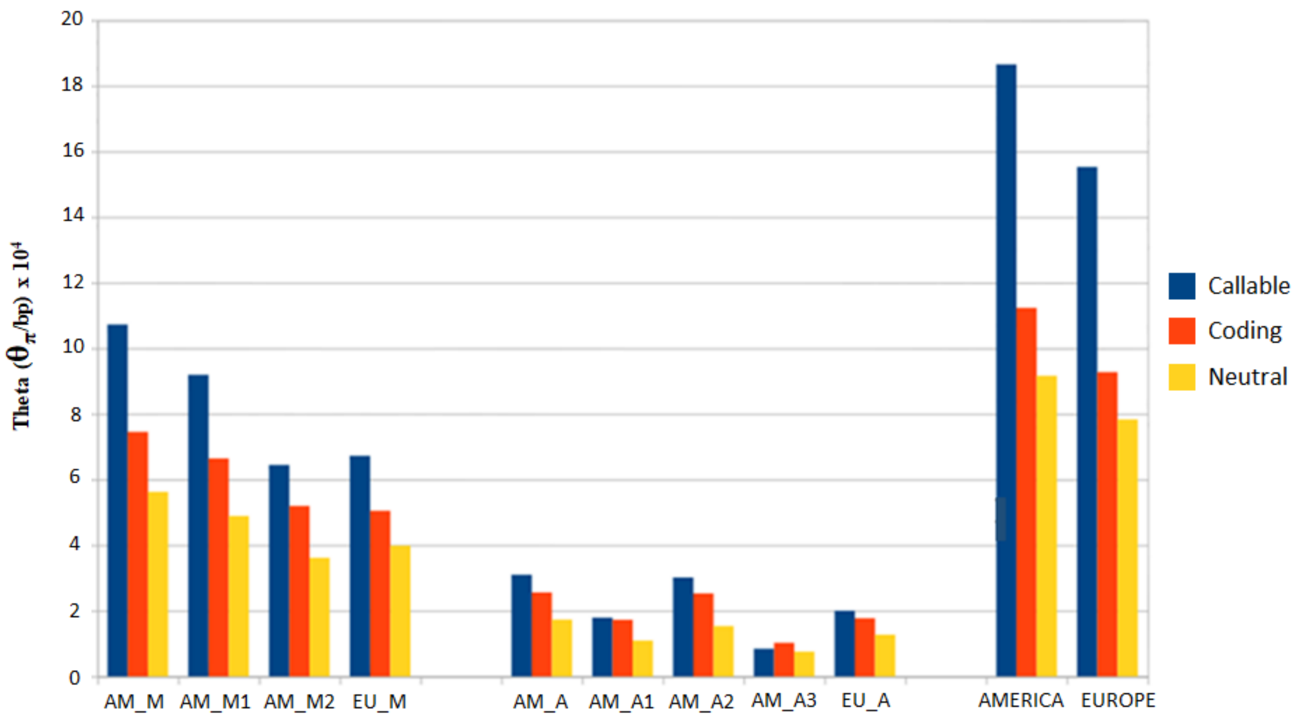

Supplementary Figure 47. Barplot of the per-site θ_π estimate (x 10^4) in the admixture masked dataset (PIND=0.3), for each group and genomic partition.

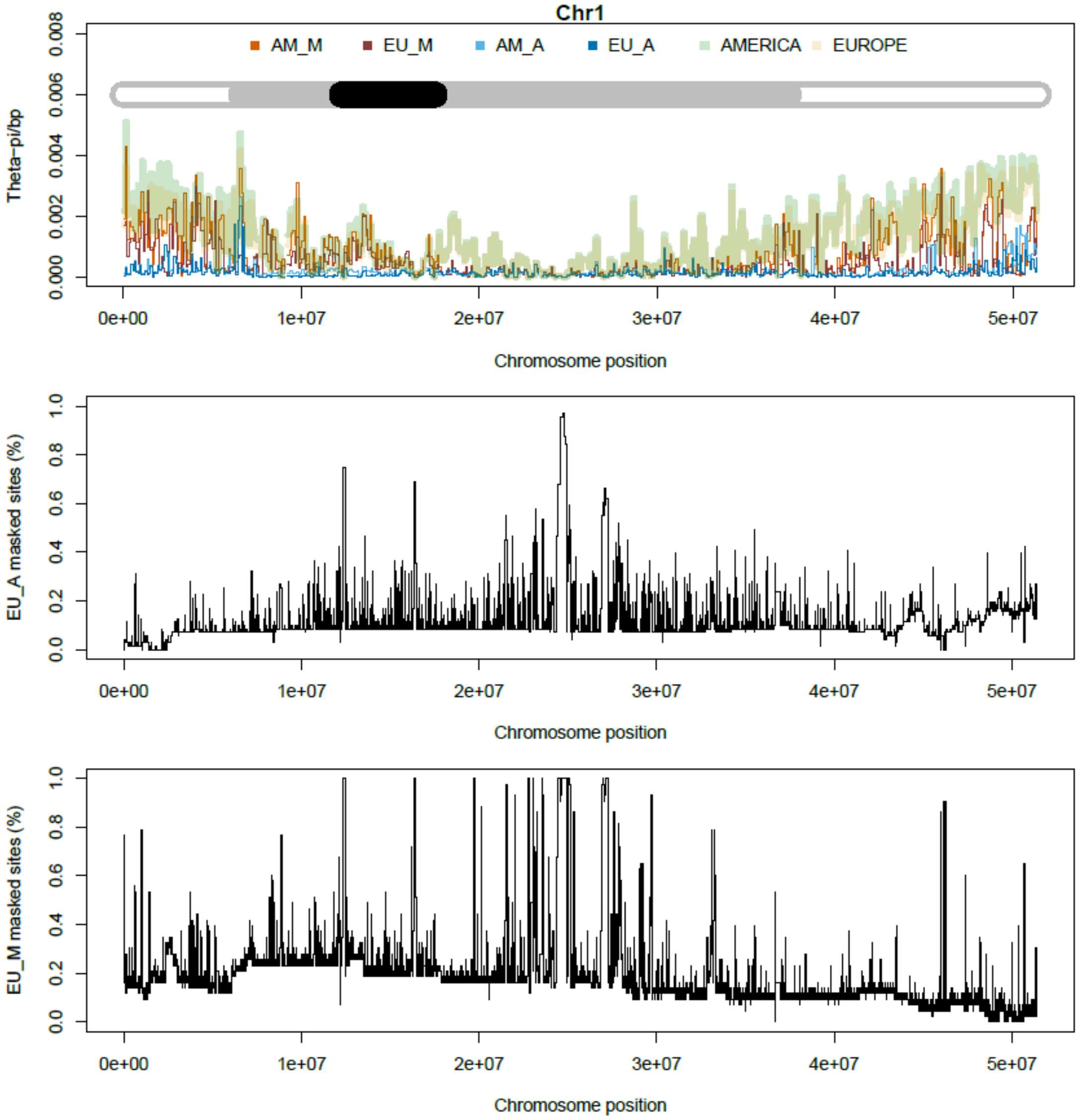

Supplementary Figure 48. Genetic diversity along the Chr01 common bean chromosome after the admixture masking. The per-site θ_π was averaged over 100kb not-overlapping sliding windows. Centromeric (black) and pericentromeric (gray) regions for each chromosome are reported. Different colored lines refer to different groups of accessions.

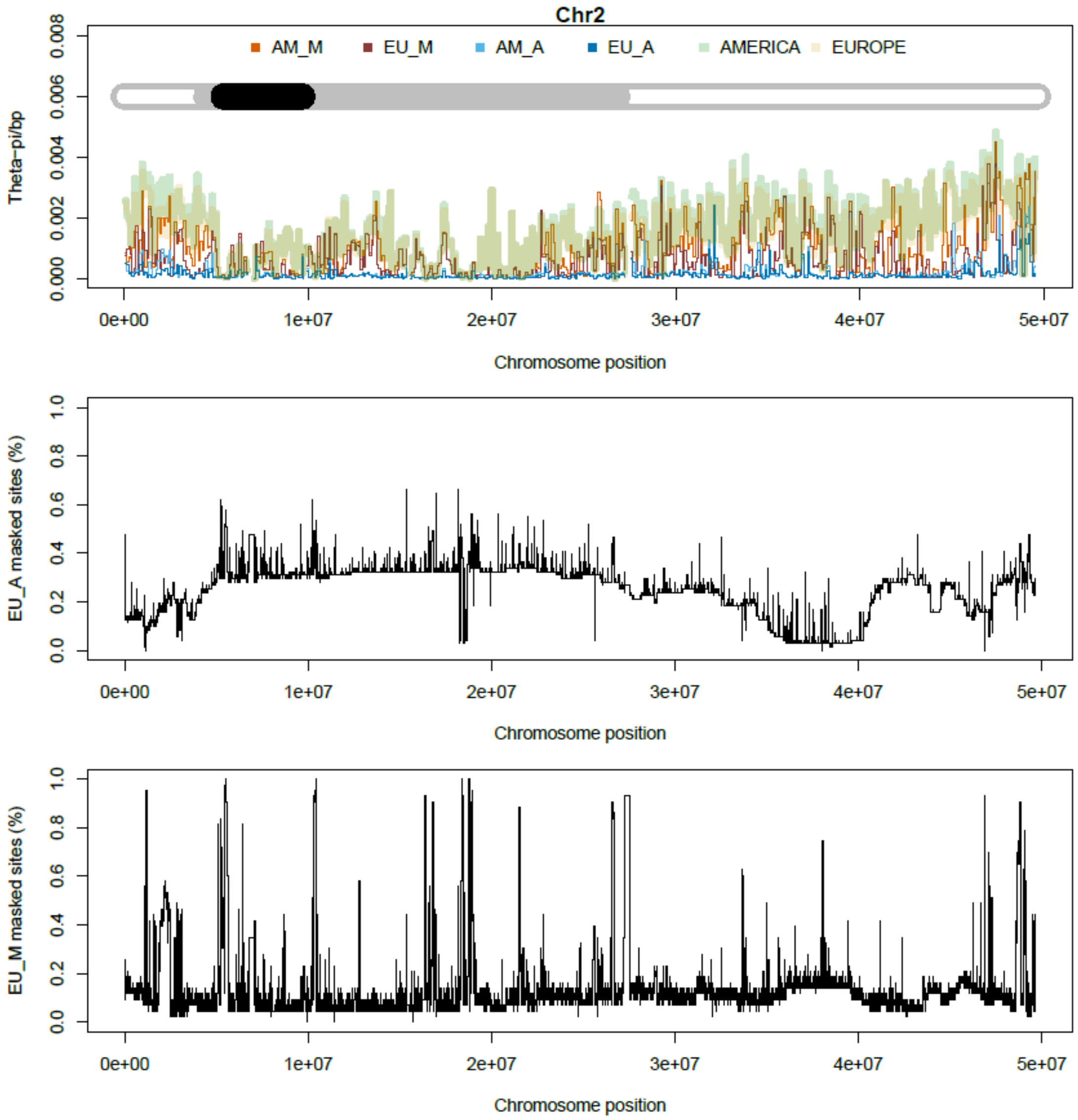

Supplementary Figure 49. Genetic diversity along the Chr02 common bean chromosome after the admixture masking. The per-site θ_{π} was averaged over 100kb not-overlapping sliding windows. Centromeric (black) and pericentromeric (gray) regions for each chromosome are reported. Different colored lines refer to different groups of accessions.

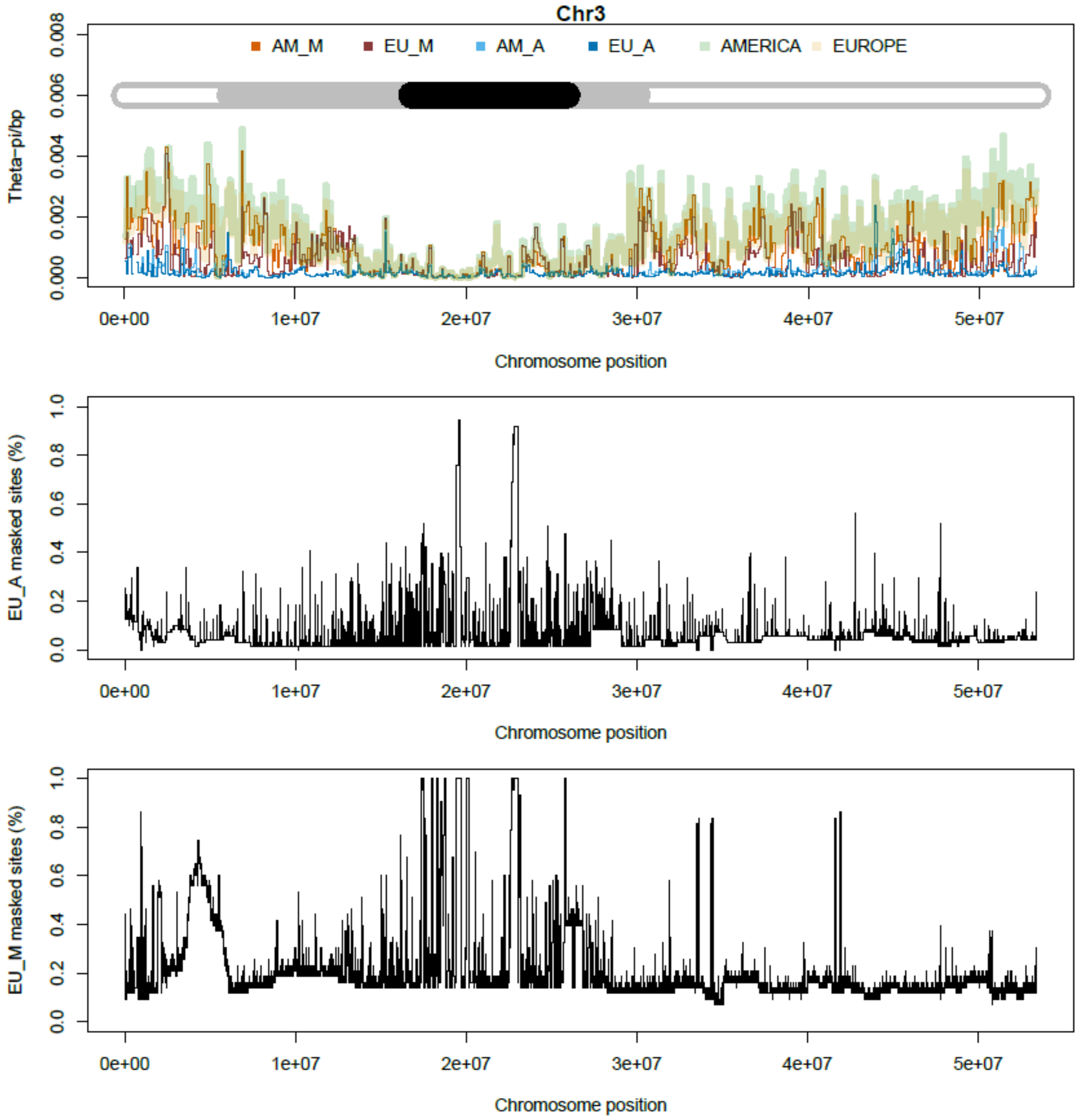

Supplementary Figure 50. Genetic diversity along the Chr03 common bean chromosome after the admixture masking. The per-site θ_π was averaged over 100kb not-overlapping sliding windows. Centromeric (black) and pericentromeric (gray) regions for each chromosome are reported. Different colored lines refer to different groups of accessions.

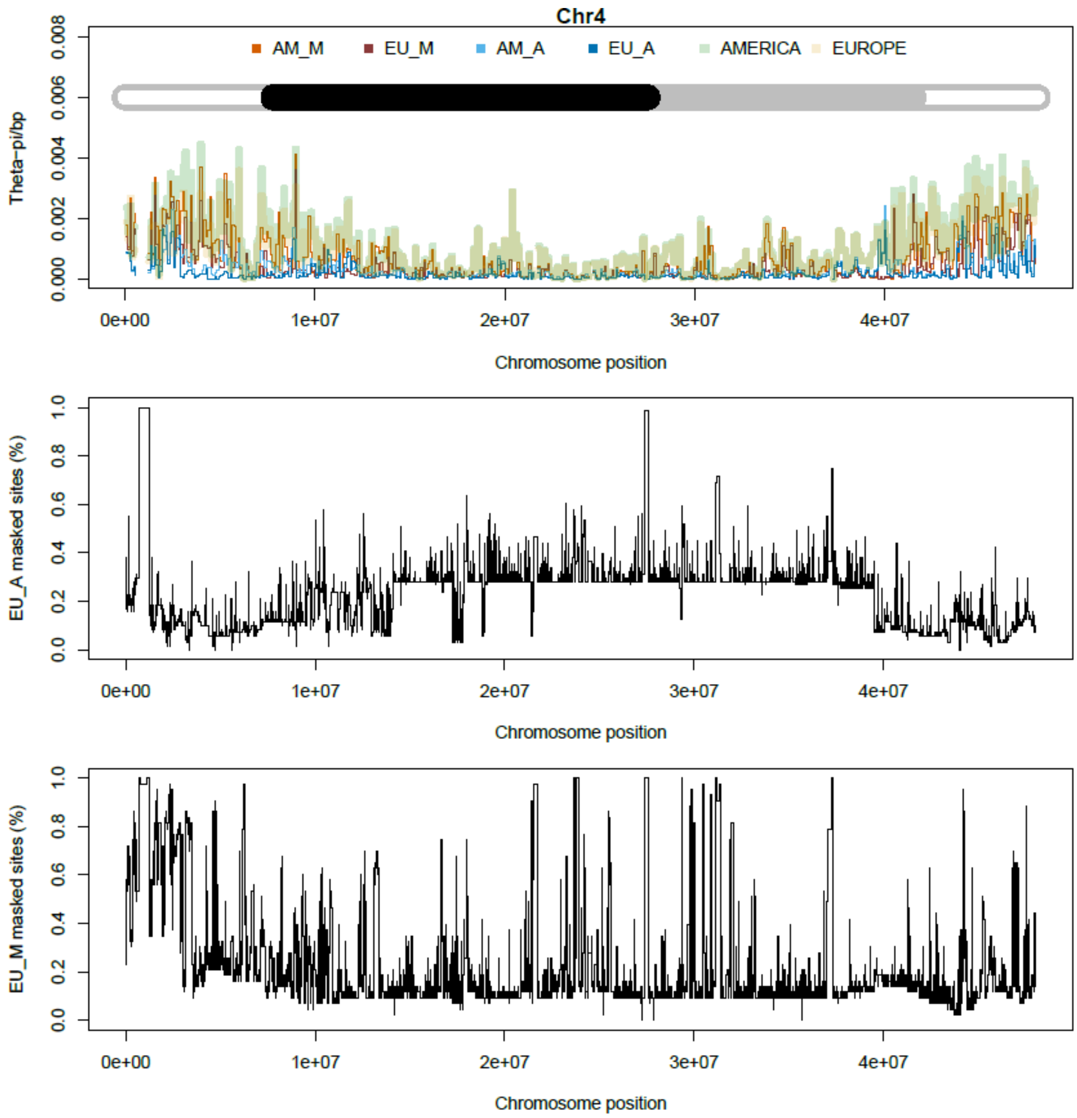

Supplementary Figure 51. Genetic diversity along the Chr04 common bean chromosome after the admixture masking. The per-site θ_π was averaged over 100kb not-overlapping sliding windows. Centromeric (black) and pericentromeric (gray) regions for each chromosome are reported. Different colored lines refer to different groups of accessions.

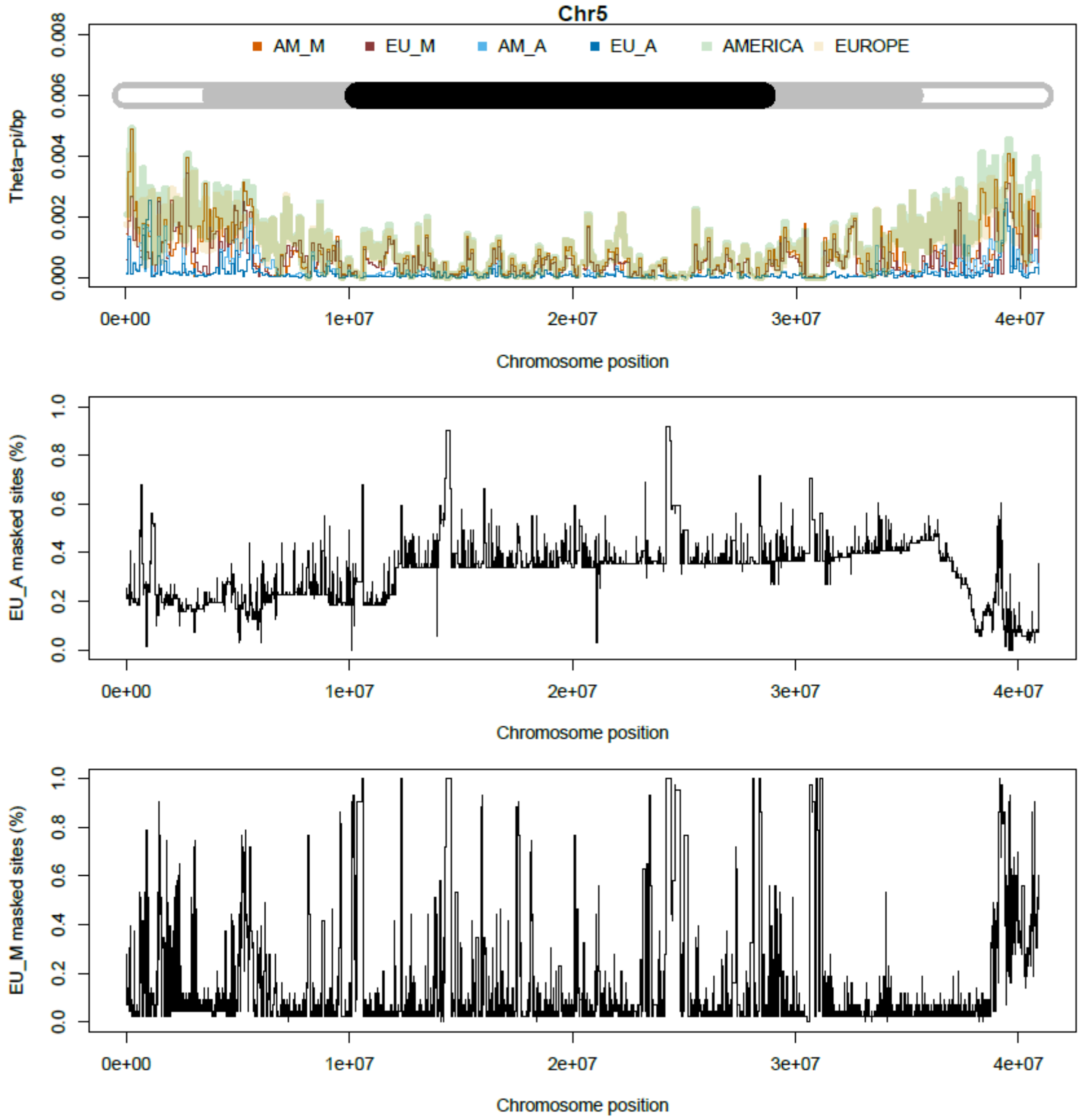

Supplementary Figure 52. Genetic diversity along the Chr05 common bean chromosome after the admixture masking. The per-site θ_π was averaged over 100kb not-overlapping sliding windows. Centromeric (black) and pericentromeric (gray) regions for each chromosome are reported. Different colored lines refer to different groups of accessions.

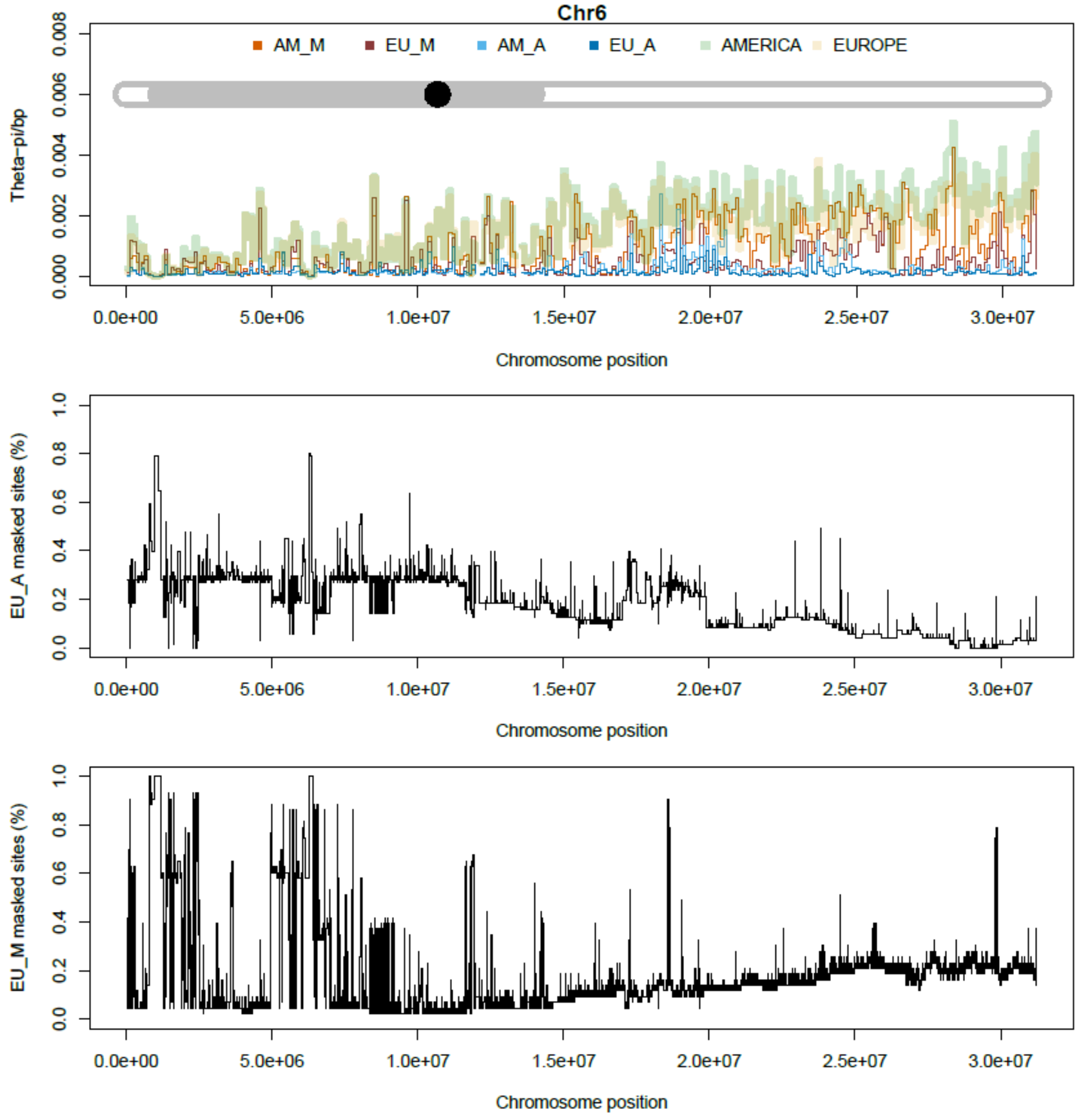

Supplementary Figure 53. Genetic diversity along the Chr06 common bean chromosome after the admixture masking. The per-site θ_π was averaged over 100kb not-overlapping sliding windows. Centromeric (black) and pericentromeric (gray) regions for each chromosome are reported. Different colored lines refer to different groups of accessions.

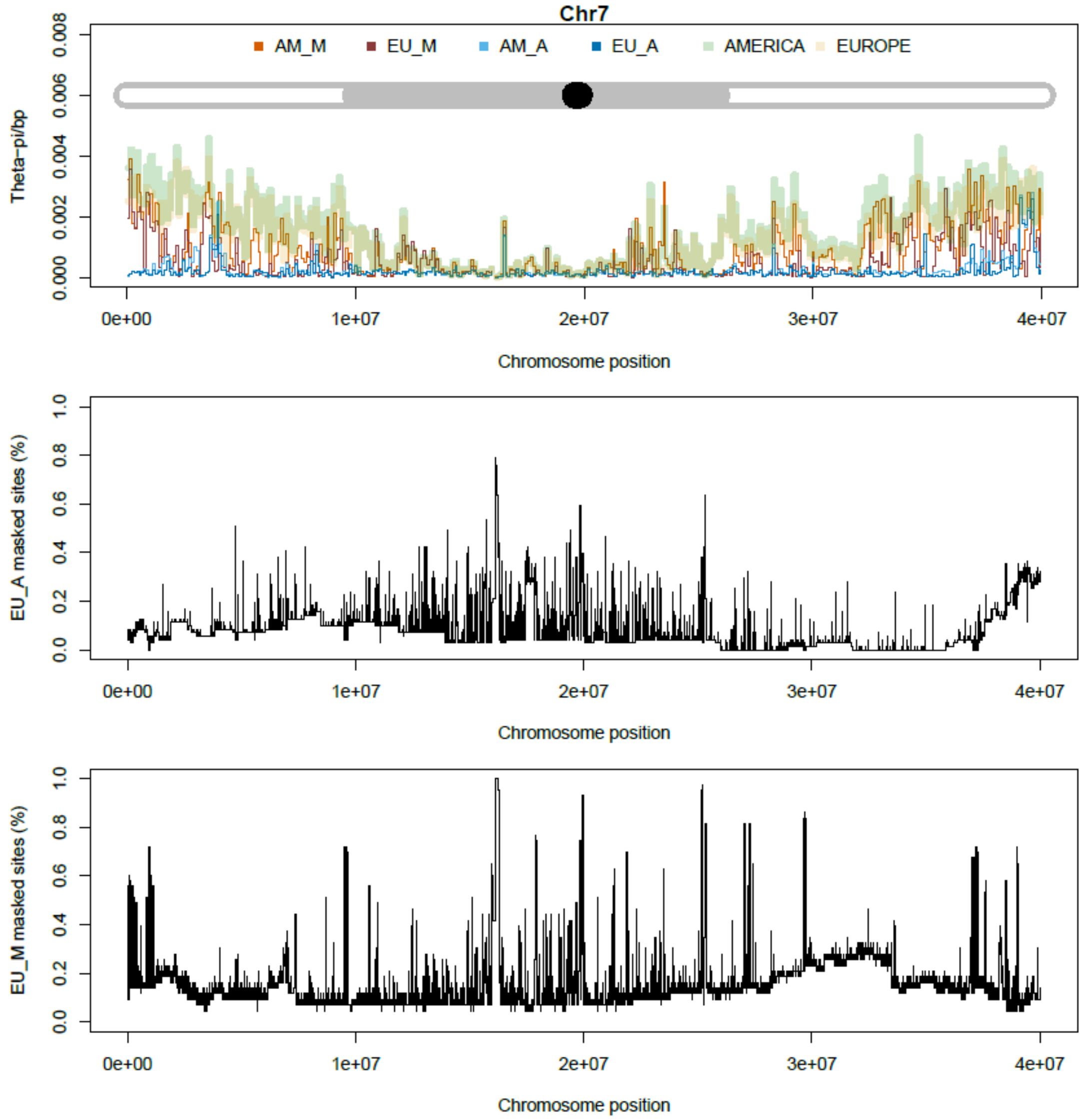

Supplementary Figure 54. Genetic diversity along the Chr07 common bean chromosome after the admixture masking. The per-site θ_π was averaged over 100kb not-overlapping sliding windows. Centromeric (black) and pericentromeric (gray) regions for each chromosome are reported. Different colored lines refer to different groups of accessions.

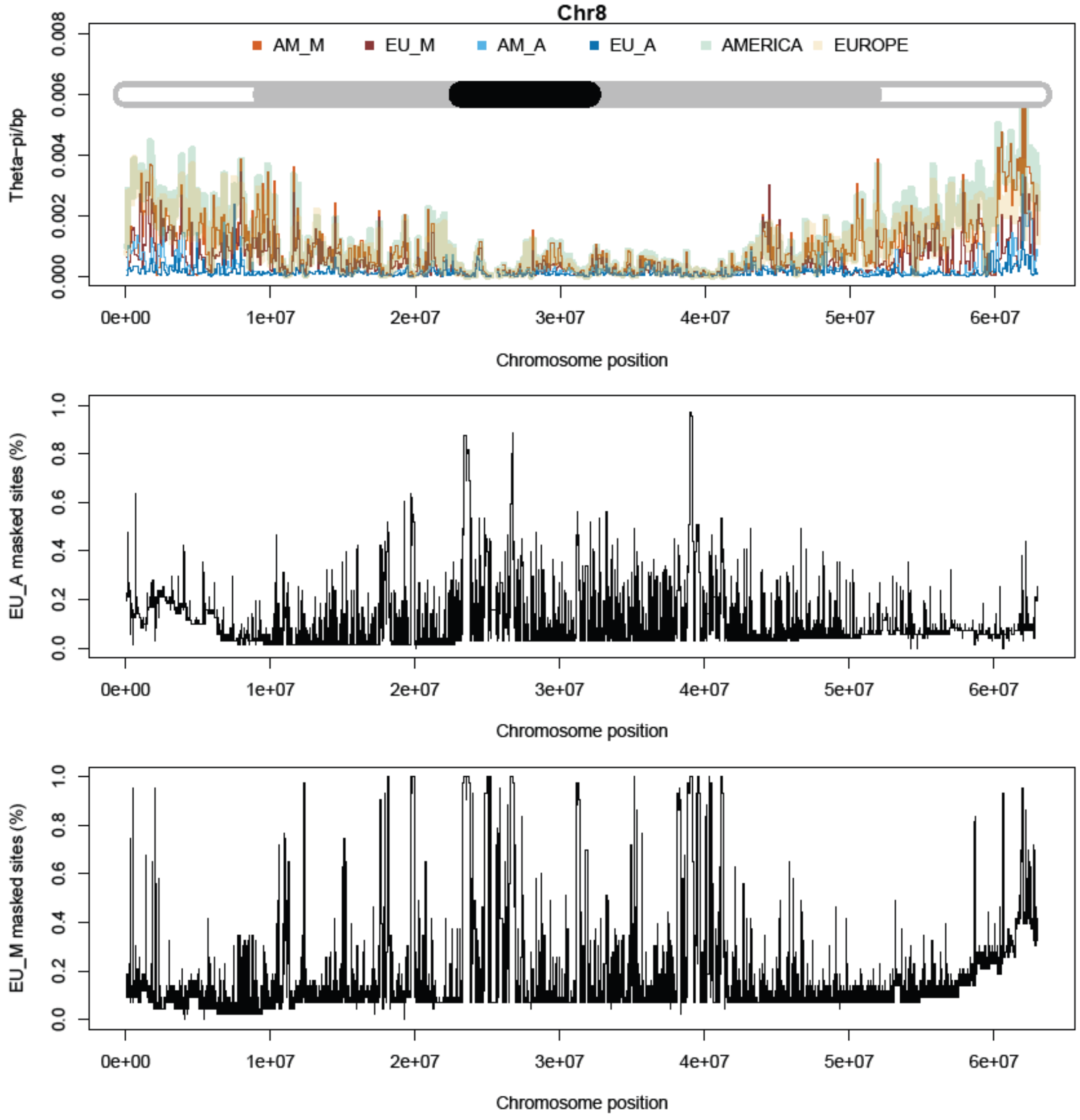

Supplementary Figure 55. Genetic diversity along the Chr08 common bean chromosome after the admixture masking. The per-site θ_π was averaged over 100kb not-overlapping sliding windows. Centromeric (black) and pericentromeric (gray) regions for each chromosome are reported. Different colored lines refer to different groups of accessions.

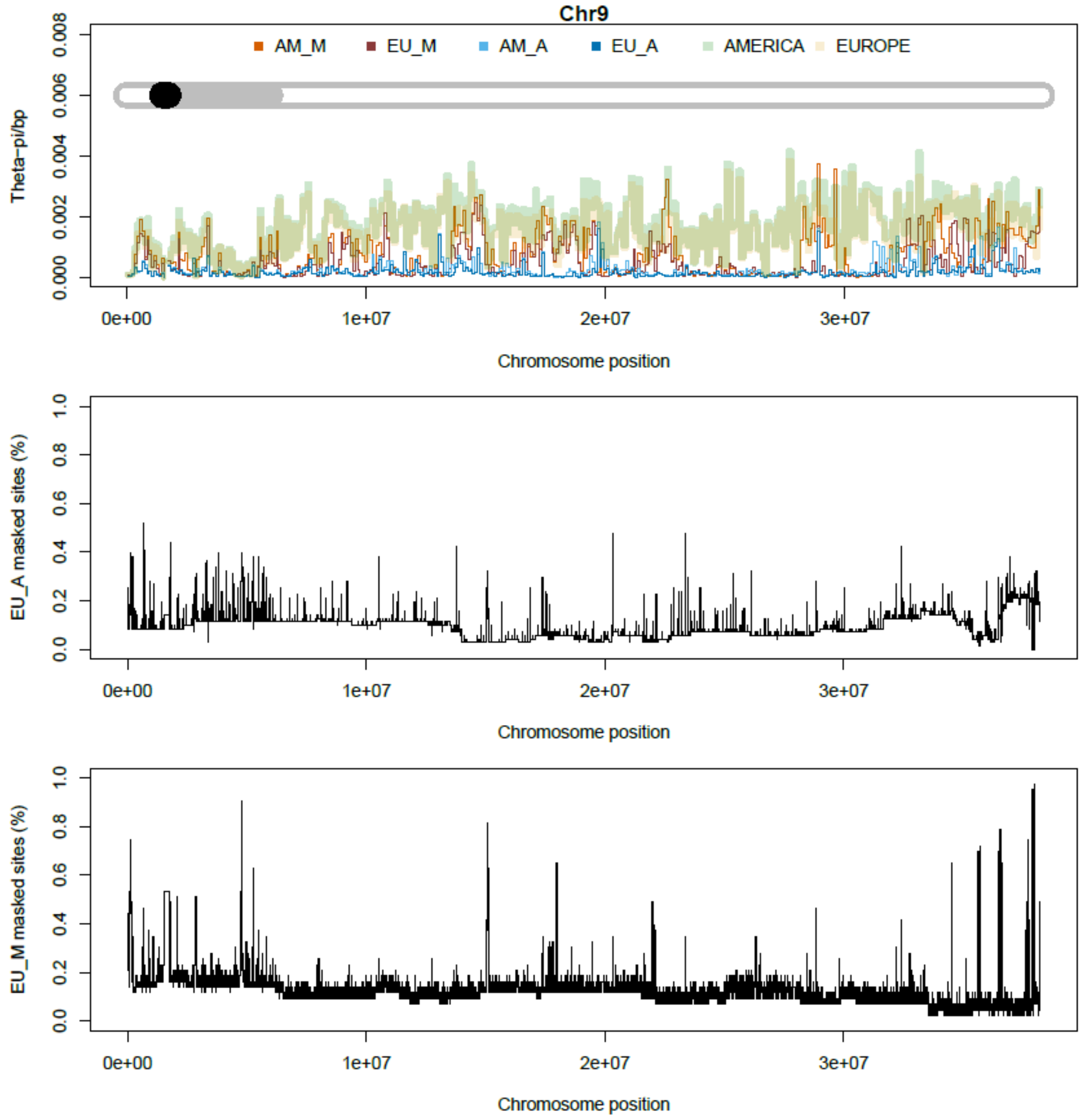

Supplementary Figure 56. Genetic diversity along the Chr09 common bean chromosome after the admixture masking. The per-site θ_π was averaged over 100kb not-overlapping sliding windows. Centromeric (black) and pericentromeric (gray) regions for each chromosome are reported. Different colored lines refer to different groups of accessions.

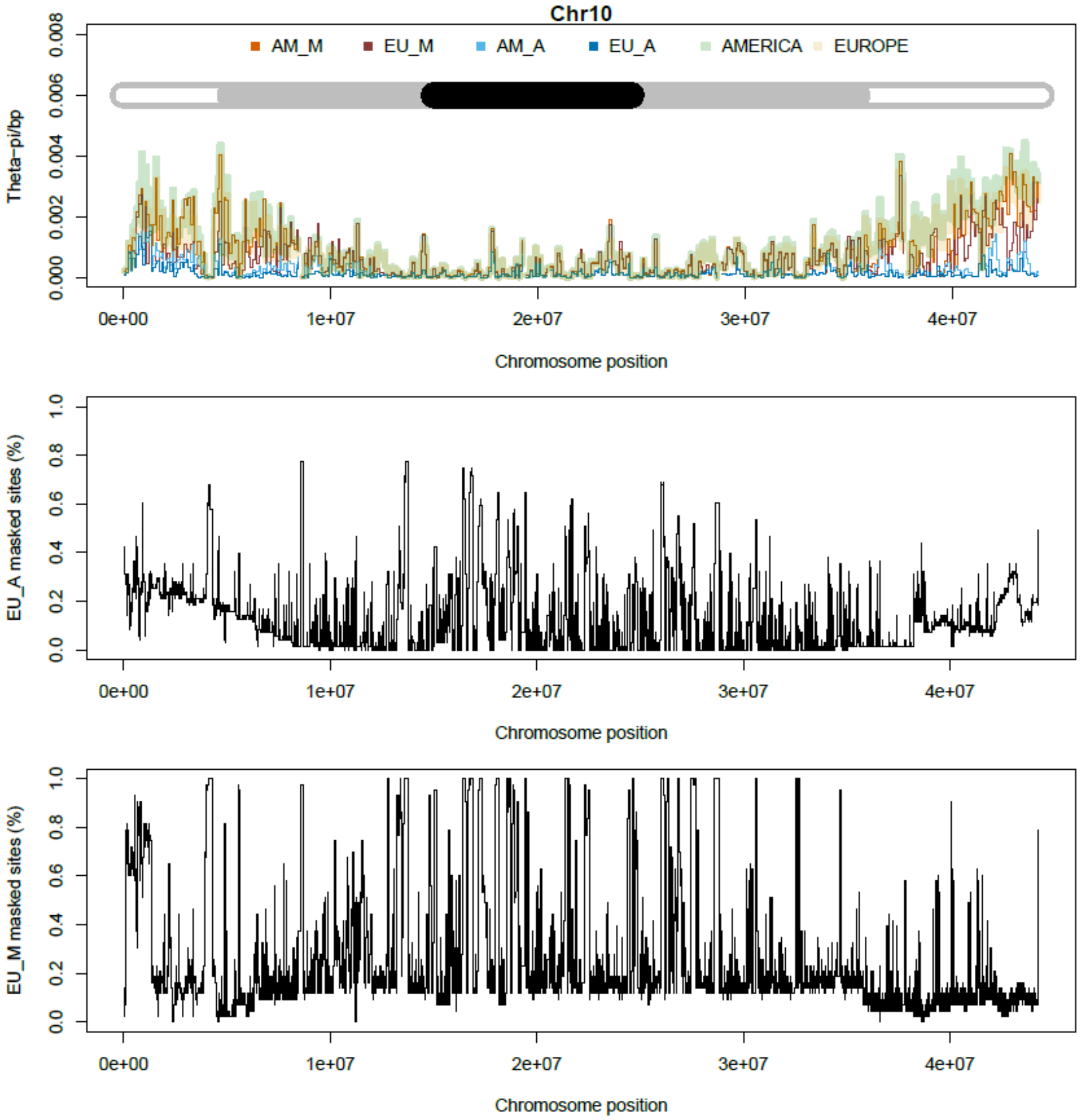

Supplementary Figure 57. Genetic diversity along the Chr10 common bean chromosome after the admixture masking. The per-site θ_π was averaged over 100kb not-overlapping sliding windows. Centromeric (black) and pericentromeric (gray) regions for each chromosome are reported. Different colored lines refer to different groups of accessions.

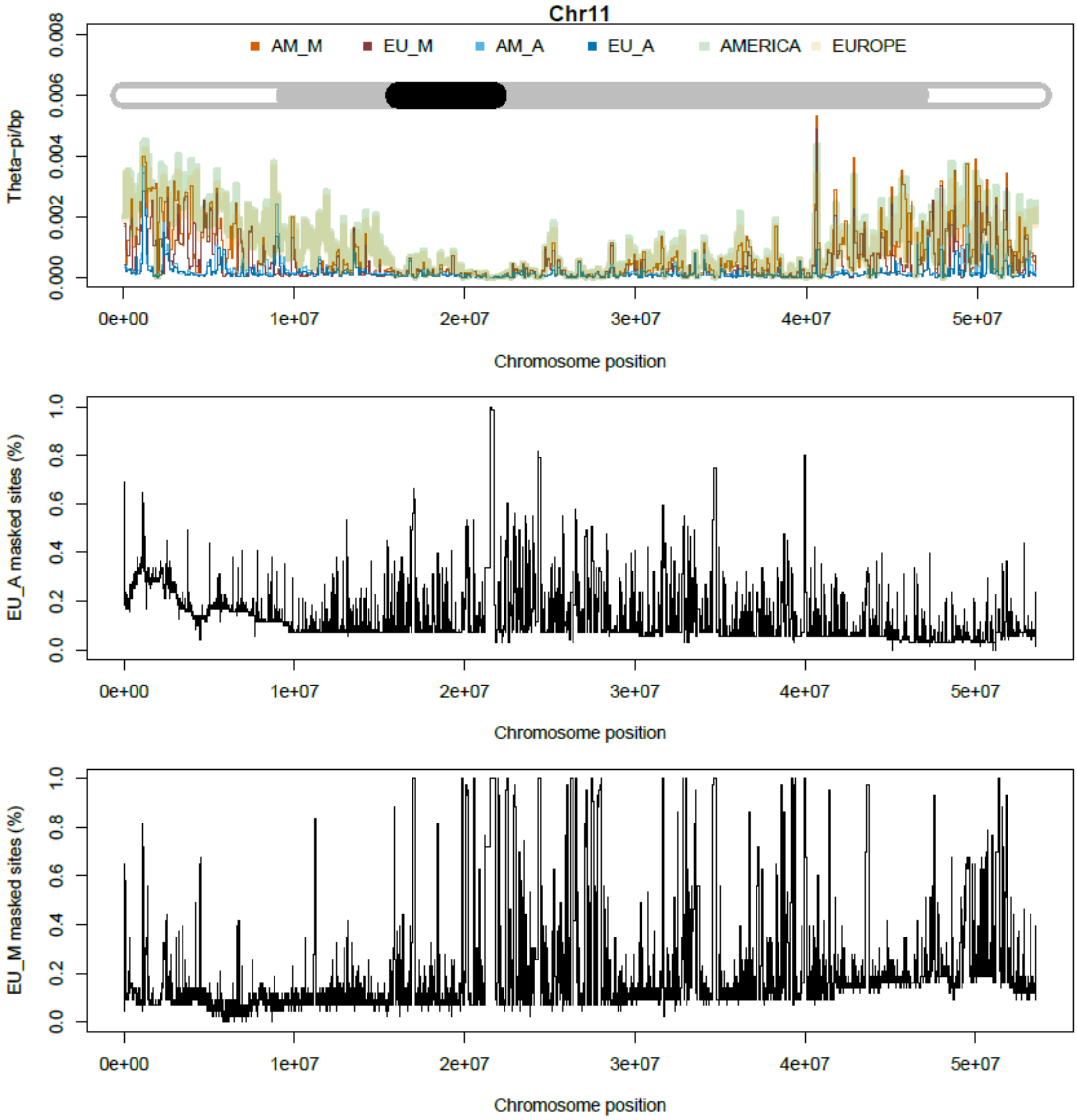

Supplementary Figure 58. Genetic diversity along the Chr11 common bean chromosome after the admixture masking. The per-site θ_π was averaged over 100kb not-overlapping sliding windows. Centromeric (black) and pericentromeric (gray) regions for each chromosome are reported. Different colored lines refer to different groups of accessions.

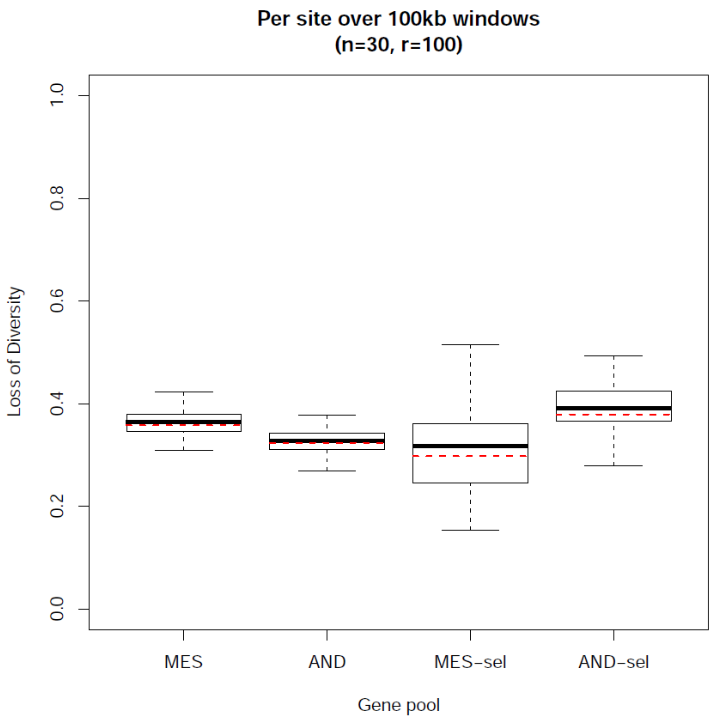

Supplementary Figure 59. The loss of diversity (LoD) in the Mesoamerican and Andean gene pools across 100 replicates having *n*=30, computed over the whole genome (MES, AND) or only in regions under selection (MES-sel, AND-sel). Black lines mark the median LoD score across replicates. The red dotted line indicates the estimated LoD without resampling the individuals. N=100 replicates for each boxplot). Box plots represent minimum, first quartile, median, third quartile and maximum.

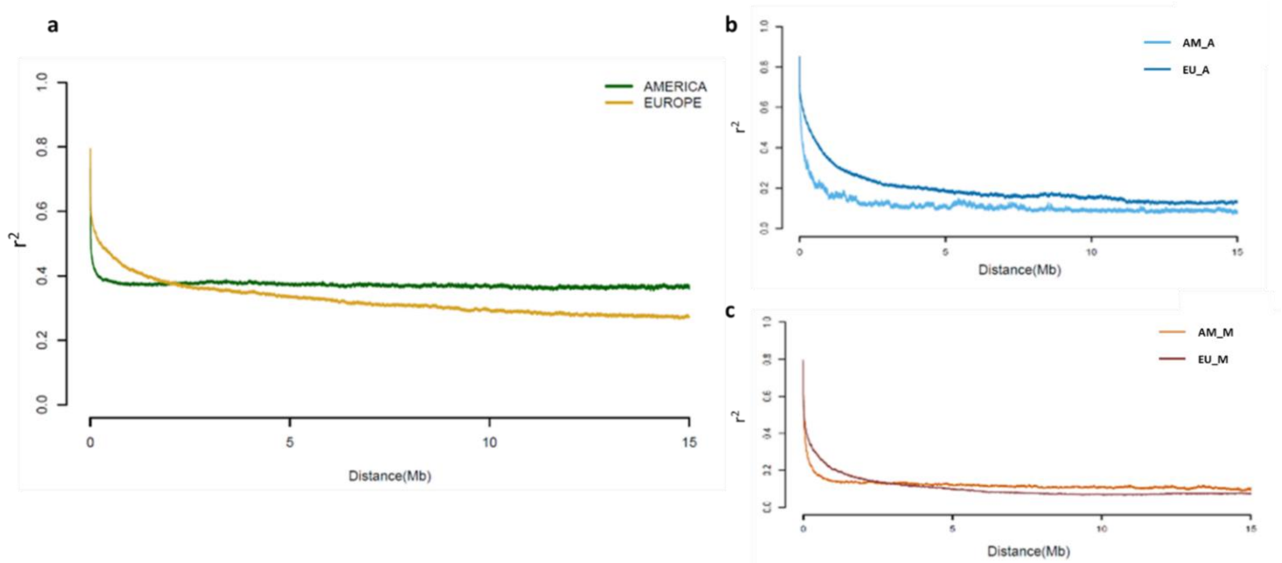

Supplementary Figure 60. LD decay in America and Europe at both the gene pool. a, LD decay comparing American and European accessions; **b**, LD decay in the Andean gene pool in Europe (EU_A) and in America (AM_A); **c**, LD decay in the Mesoamerican gene pool in Europe (EU_M) and in America (AM_M).

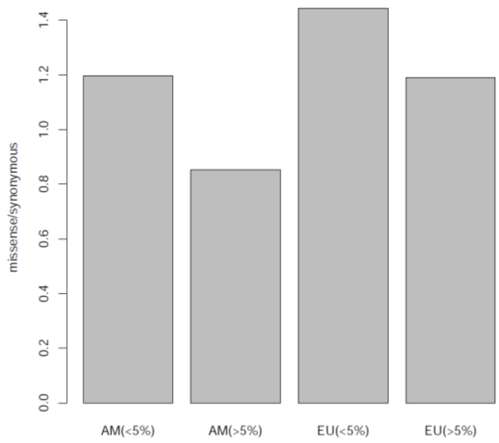

Supplementary Figure 61. Genome-wide genetic load evaluated at American or European private alleles, segregating at low (<5%) or high (>5%) frequencies.

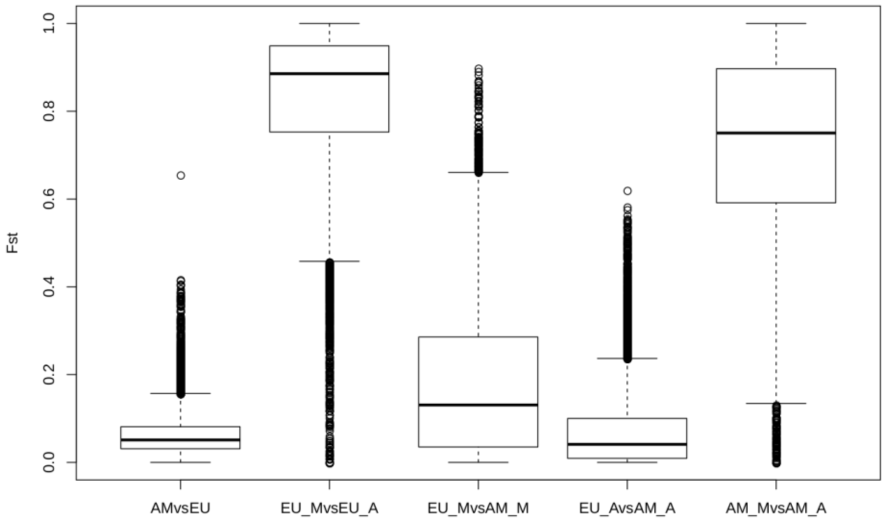

Supplementary Figure 62. Boxplot of the F_{ST} distribution computed in 10Kb not overlapping windows composed by more than 90% of callable regions. AM *vs* EU=8814, EU_M *vs* EU_A=8794, EU_M *vs* AM_M=8747, EU_A *vs* AM_A=8607, AM_M *vs* AM_A=8815, genomic windows. Box plots represent minimum, first quartile, median, third quartile and maximum.

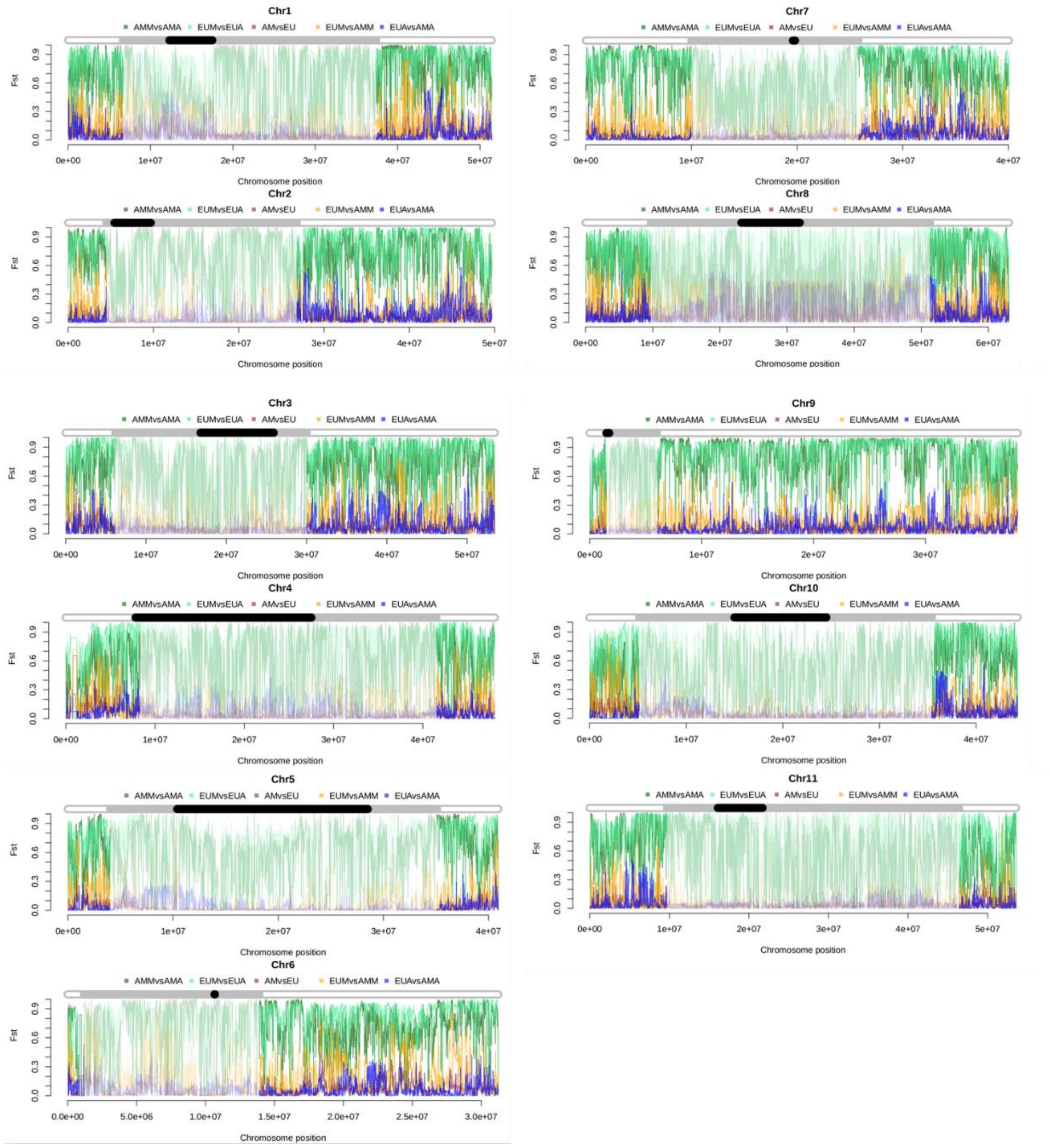

Supplementary Figure 63. F_{ST} variation along chromosomes.

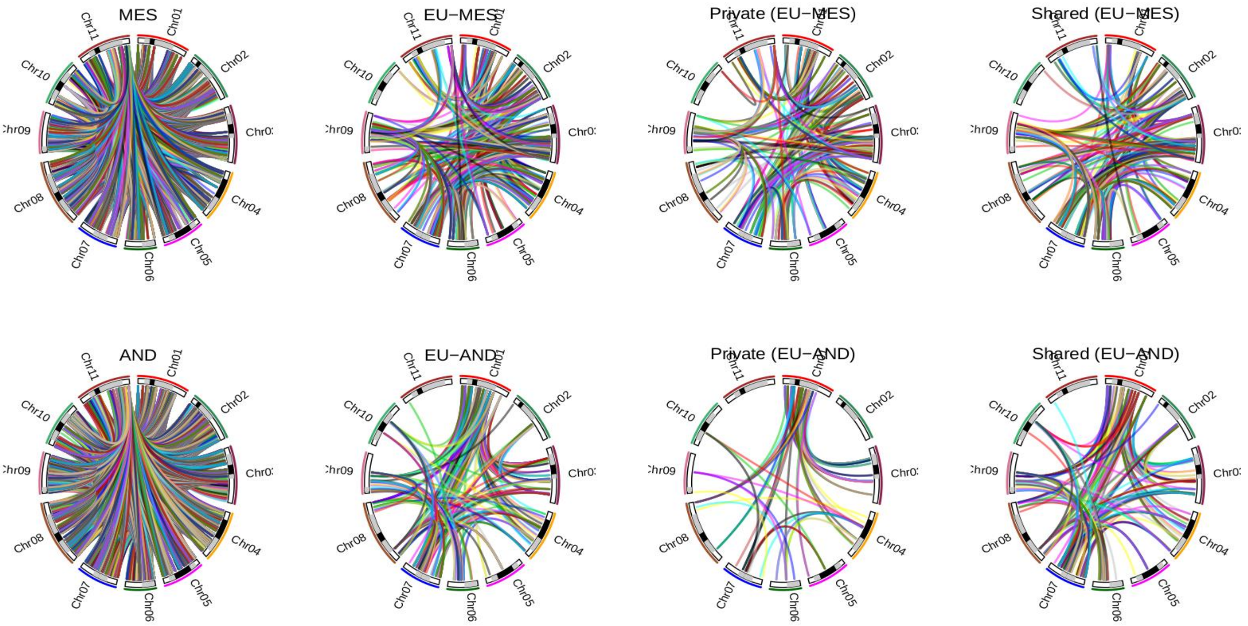

Supplementary Figure 64. Inter-chromosomal linkage disequilibrium in *P. vulgaris* over whole chromosomes. Colored links connect regions >=500kb showing an r^2 >= 0.8. Private and shared links refers to the European groups.

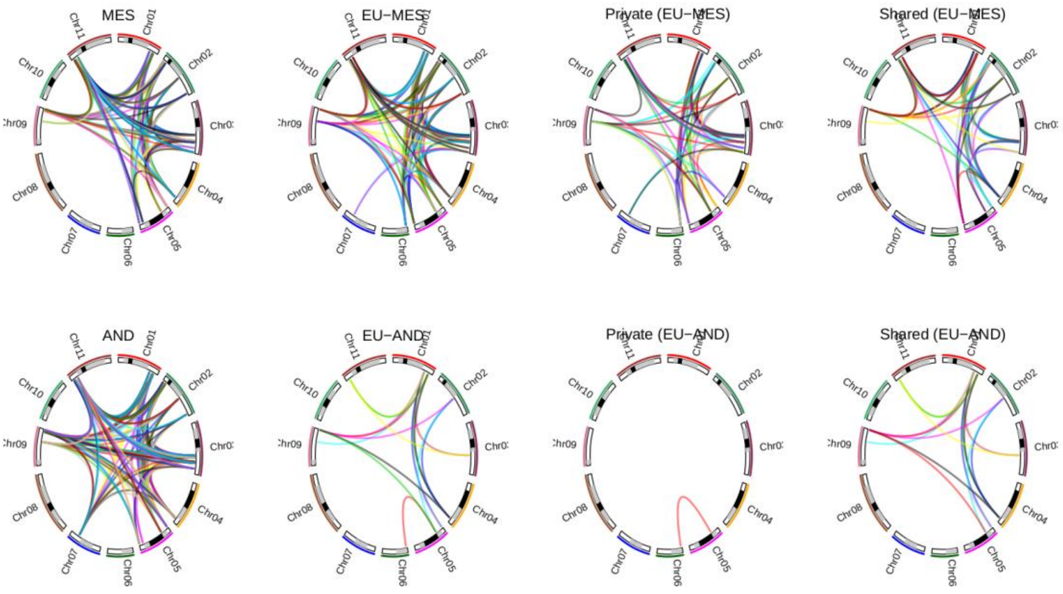

Supplementary Figure 65. Inter-chromosomal linkage disequilibrium in *P. vulgaris* over the "Extended" set of regions under selection. Colored links connect regions >=50kb showing a r^2 >= 0.8. Private and shared links refers to the European groups.

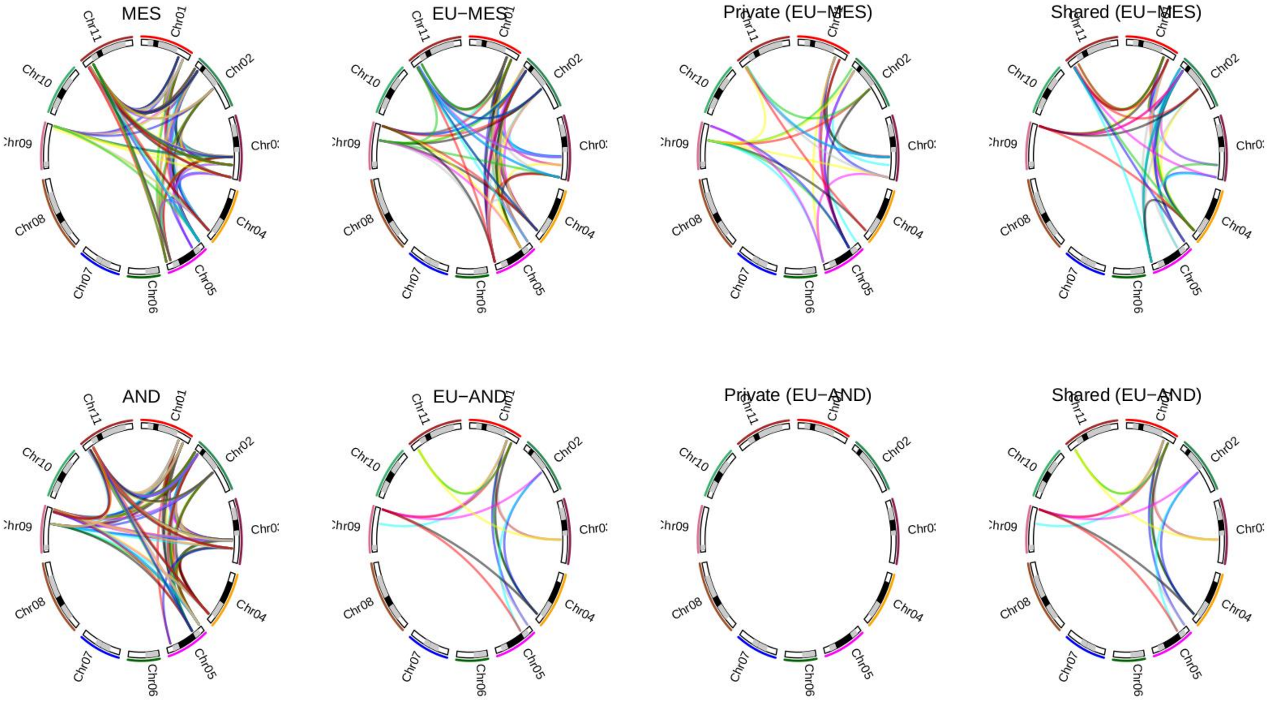

Supplementary Figure 66. Inter-chromosomal linkage disequilibrium in *P. vulgaris* over the "Restricted" set of regions under selection. Colored links connect regions >=50kb showing an r^2 >= 0.8. Private and shared links refers to the European groups.

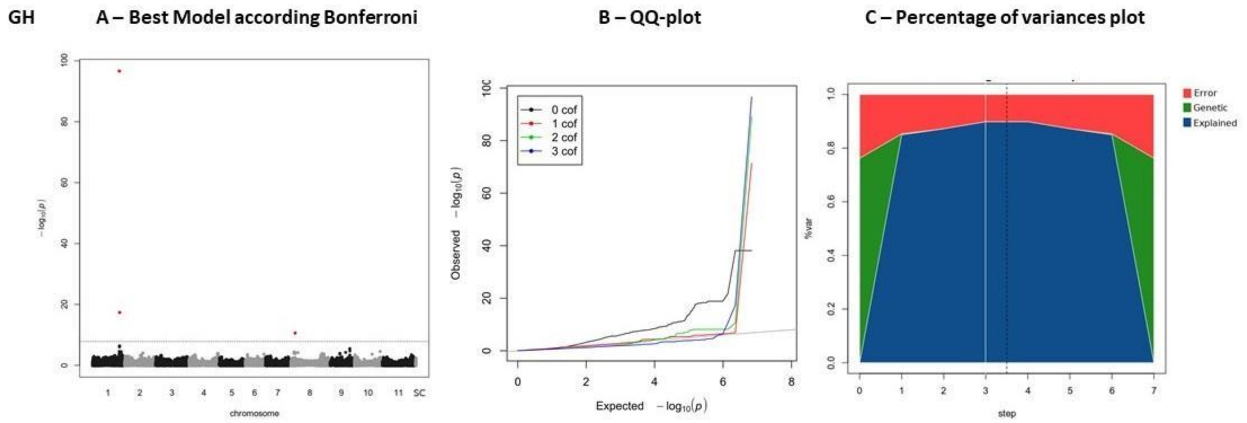

Supplementary Figure 67. Manhattan-plot (A), QQ-plot (B) and partition of variance plot (C) obtained from MLMM analysis on GH. (A) Red dots highlight the SNPs significantly associated with the trait. The dotted line indicates the Bonferroni corrected threshold at α = 0.05 (p = 2.91E-09). SC indicates the SNPs located on different scaffolds. **(B)** Each colored line shows the observed -logPvalues when markers are progressively added as cofactors into the model (p-value; two-sided, after Bonferroni correction). **(C)** The three differently colored areas indicate the proportion of variance components explained by the model when the associated SNPs are progressively added to the model and then removed (three forward and three backward steps). The white vertical line indicates the variance at the optimal model.

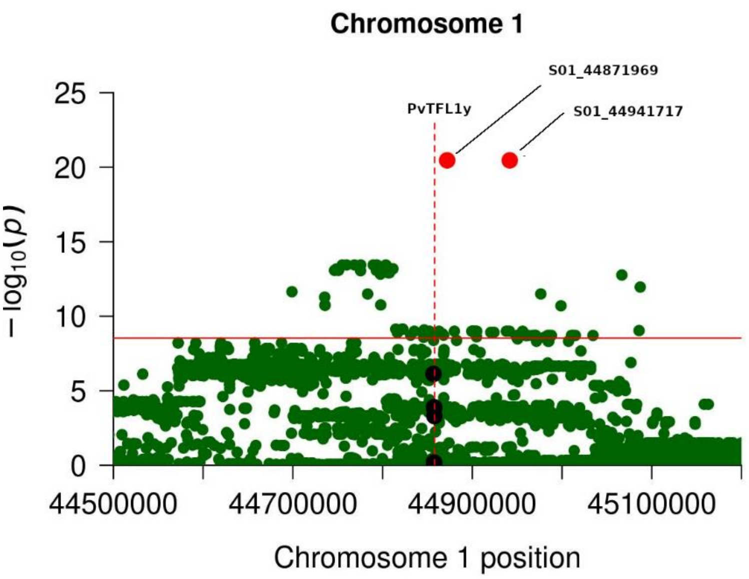

Supplementary Figure 68. Manhattan-plot obtained from MLM analysis on GH. Zoom in the region of Chromosome Pv01 where *PvTFL1y* (red dotted line) and the SNPs within it (black dots) are located. Red dots; significant SNPs associated to the GH trait (determinacy *vs* indeterminacy). The red horizontal line indicates the Bonferroni threshold at α = 0.05 (p = 2.911E-09).

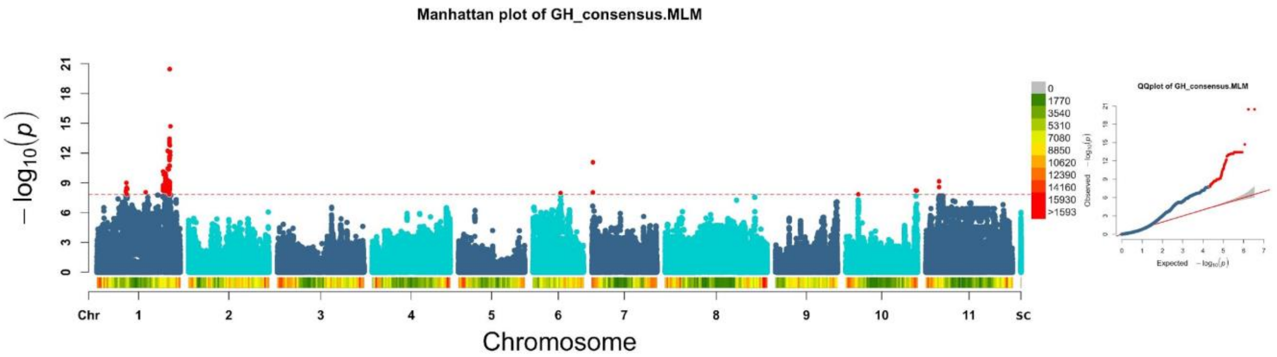

Supplementary Figure 69. Manhattan-plot (left) and QQ-plot (right) obtained from single-locus MLM analysis on growth habit (GH). Red dots highlight the SNPs significantly associated with the trait. The dashed red line indicates the Bonferroni threshold at α = 0.05 (p = 2.91E-09). SC indicates the SNPs located on scaffolds. Density map of markers (below each chromosome) and relative frequency scale (on the top right of the plot) are also illustrated.

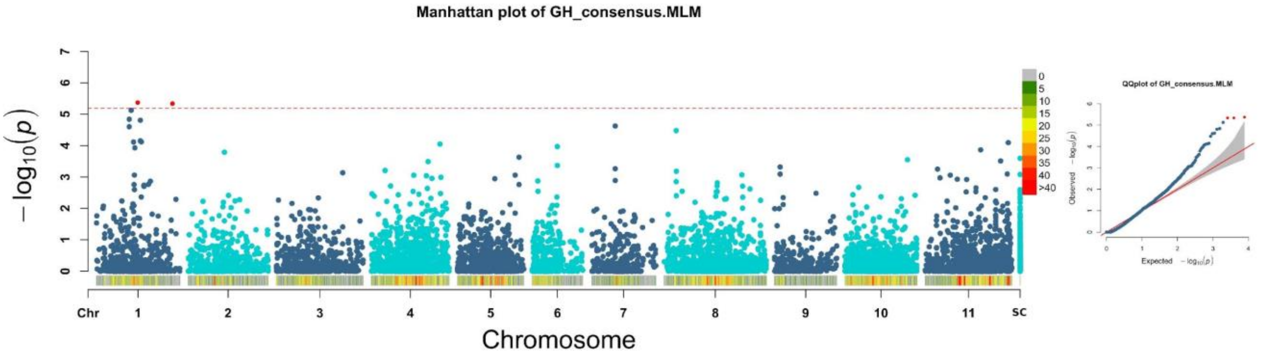

Supplementary Figure 70. Manhattan-plot (left) and QQ-plot (right) obtained from MLM analysis of CNV and growth habit (GH). Red dots highlight the CNVs significantly associated with the trait. The dashed red line indicates the Bonferroni threshold at α = 0.05 (*p*=6.484E-06). SC indicates the SNPs located on scaffolds. Density map of CNVs (below each chromosome) and relative frequency scale (on the top right of the plot) are also illustrated.

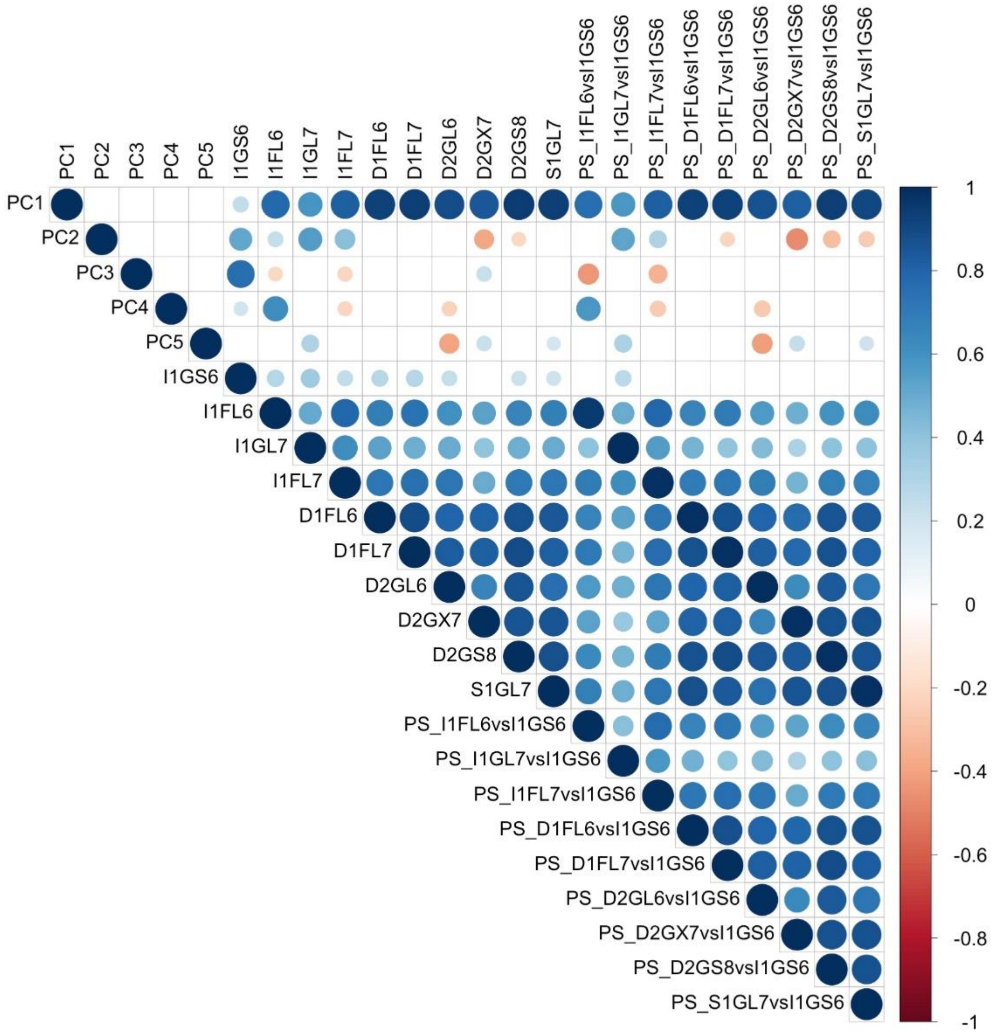

Supplementary Figure 71. Correlations between each PCs obtained from overall flowering data and DTF and PS data registered in each environment. The intensity of the color and the circle size are proportional to the correlation coefficients. Larger dark-blue dots indicate higher positive correlations, while larger red dots indicate higher negative correlations. The R corrplot package (https://github.com/taiyun/corrplot) was used to draw the correlation plot. No statistically significant associations are indicated as empty cells.

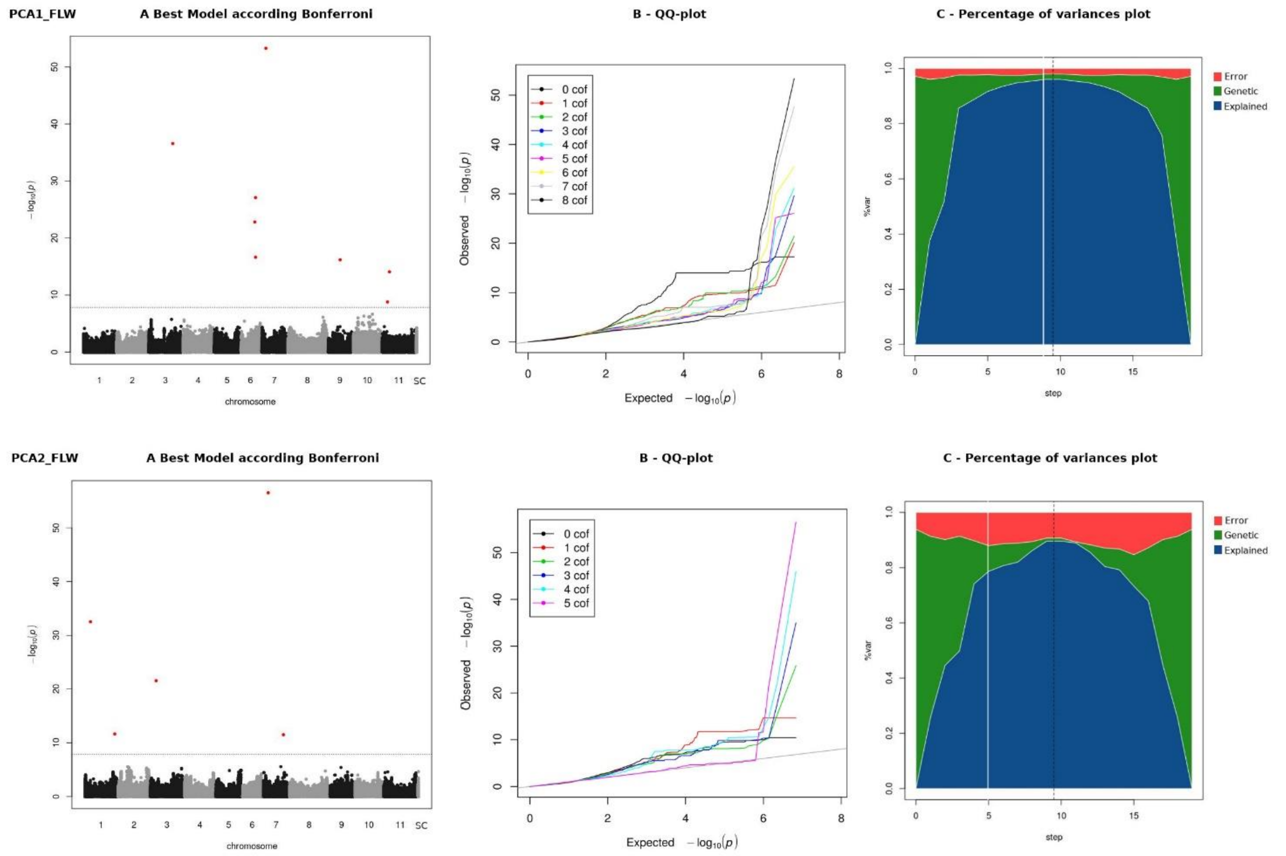

Supplementary Figure 72. Manhattan-plots (A), QQ-plots (B) and partition of variance plots (C) as obtained from MLMM analysis on the flowering principal components (PC1-PC2). (A) Red dots highlight the SNPs significantly associated with the trait. The dotted black line indicates the Bonferroni threshold at $\alpha = 0.05$ ($p = 2.91E-09$). SC indicates the SNPs located on scaffolds. **(B)** The colored lines show the observed -logPvalues when markers are progressively added as cofactors into the model (*p*-value; two-sided, after Bonferroni correction). **(C)** The three differently colored areas indicate the proportion of variance components explained by the model when the associated SNPs are progressively added to the model and then removed (forward and backward steps). The white vertical line indicates the variance at the optimal model.

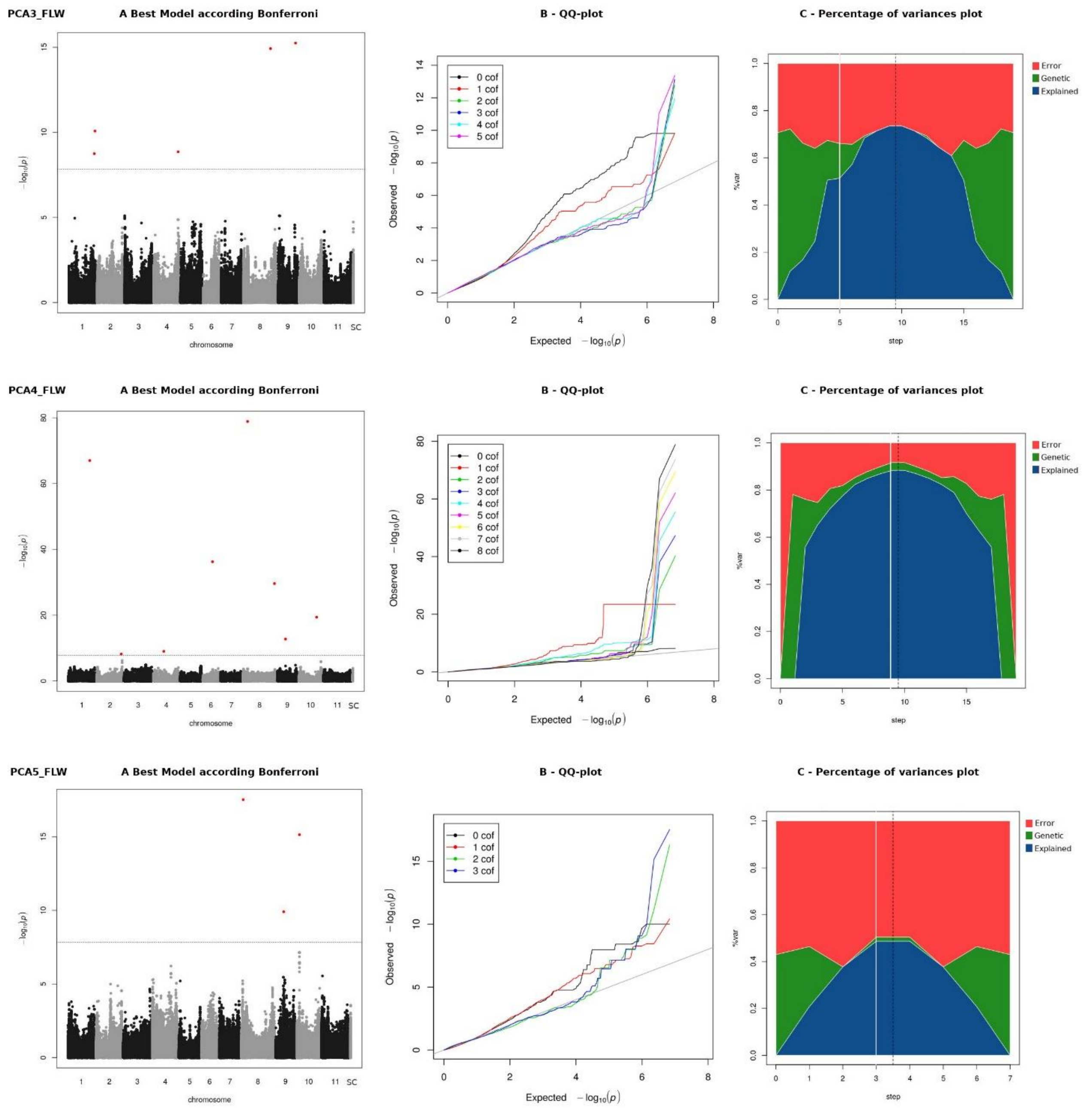

Supplementary Figure 73. Manhattan-plots (A), QQ-plots (B) and partition of variance plots (C) as obtained from MLMM analysis on the flowering principal components (PC3-PC5). (A) Red dots highlight the SNPs significantly associated with the trait. The dotted black line indicates the Bonferroni threshold at α = 0.05 (*p* = 2.91E-09). SC indicates the SNPs located on scaffolds. **(B)** The colored lines show the observed -logPvalues when markers are progressively added as cofactors into the model (*p*-value; two-sided, after Bonferroni correction). **(C)** The three differently colored areas indicate the proportion of variance components explained by the model when the associated SNPs are progressively added to the model and then removed (forward and backward steps). The white vertical line indicates the variance at the optimal model.

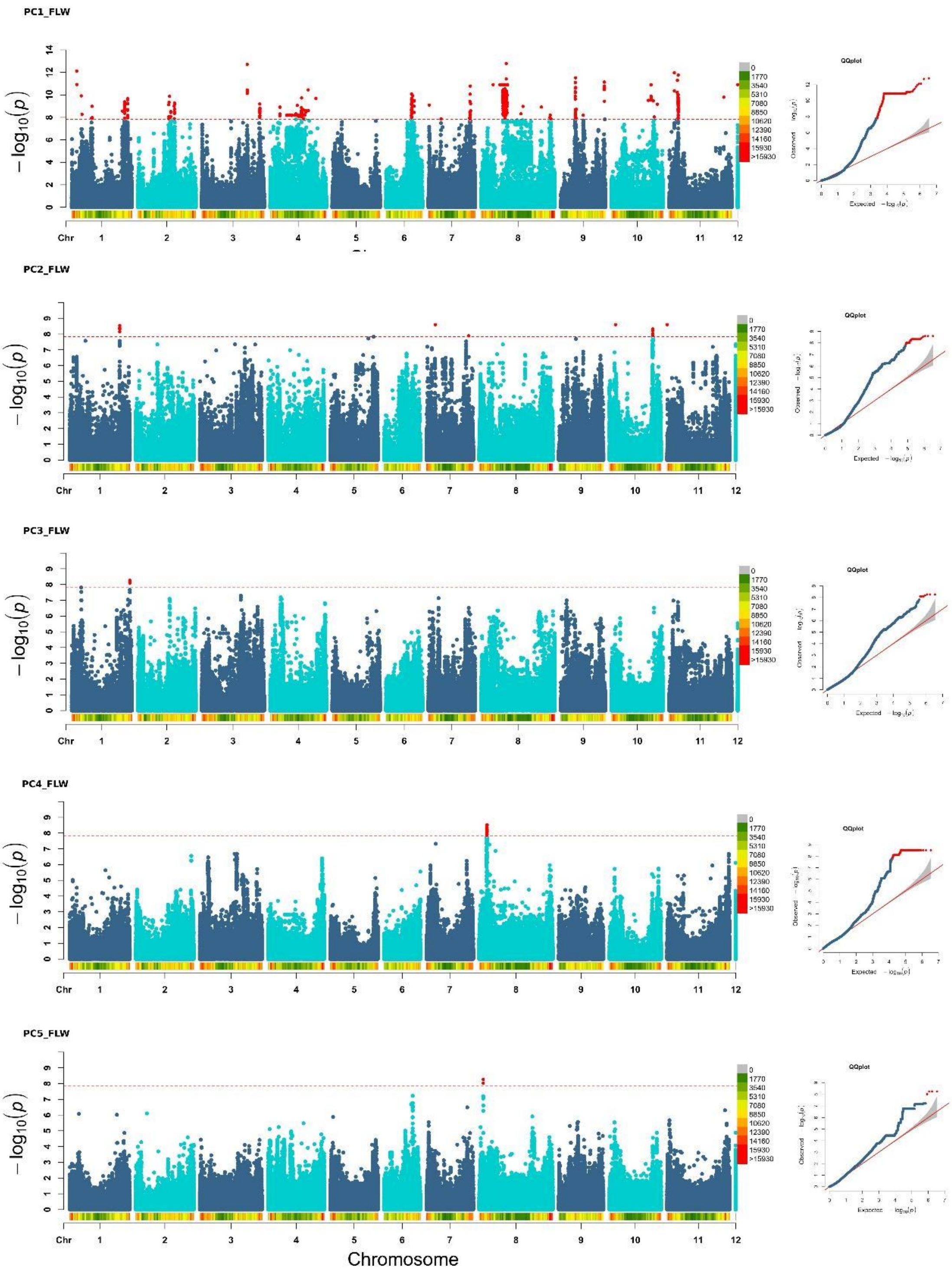

Supplementary Figure 74. Manhattan-plots (left) and QQ-plots (right) obtained from MLM analysis on SNPs and PCs on flowering (FLW). Red dots highlight the SNPs significantly associated with the trait. The dashed red line indicates the Bonferroni threshold at α = 0.05 (p = 2.91E-09). SC indicates the SNPs located on scaffolds. Density map of SNPs (below each chromosome) and relative frequency scale (the top right of the plot) are also illustrated.

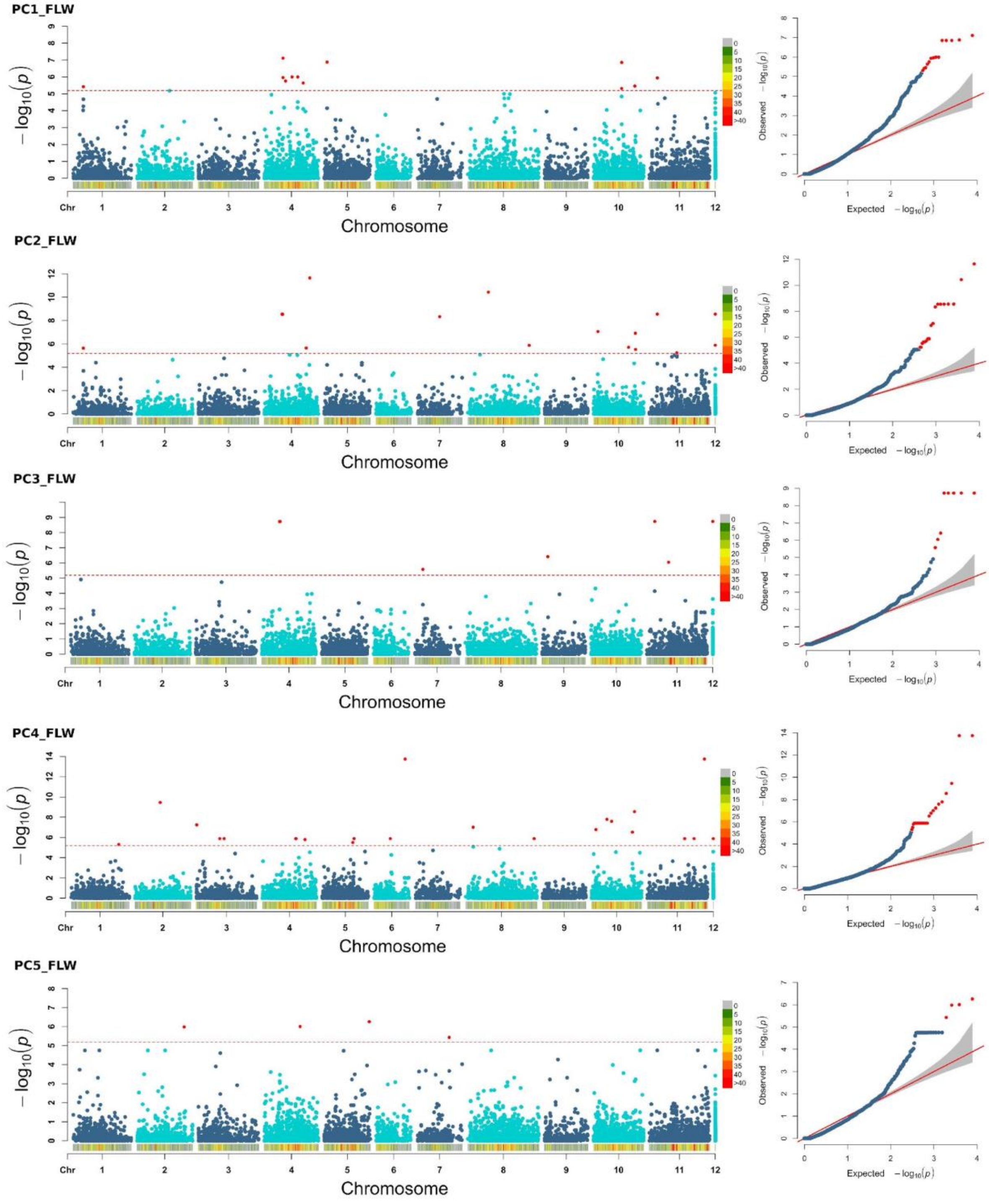

Supplementary Figure 75. Manhattan-plots (left) and QQ-plots (right) obtained from MLM analysis on CNVs and PCs on flowering (FLW). Red dots highlight the CNVs significantly associated with the trait. The dashed red line indicates the Bonferroni threshold at α = 0.05 (p = 6.484E-06). SC indicates the SNPs located on scaffolds. Density map of CNVs (below each chromosome) and relative frequency scale (on the top right of the plot) are also illustrated.

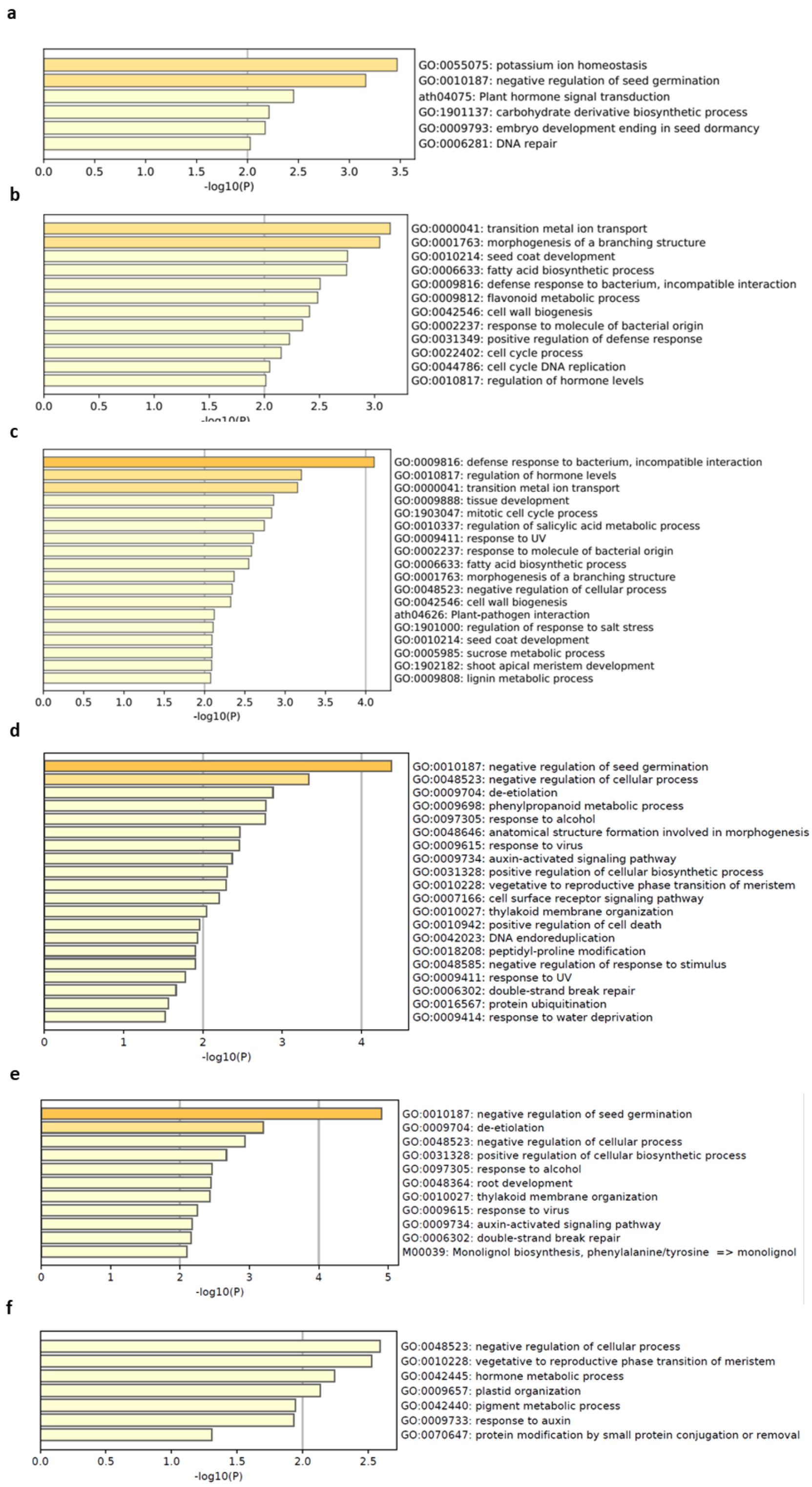

Supplementary Figure 76. Result of the gene enrichment analysis. GO classes representativity was investigated for genes located within QTLs regions (100 kb regions centered on significant GWAS peaks) associated to GH (a), and to DTF using both the MLM and the MLMM models (b and c), selection scan from the extended set (d), selection scan showing a significant F_{ST} between Europe and America (e), and adaptive introgression scan showing signature of novel selection (overlapping of selection, F_{ST} and excess of introgression signals) (f). The enrichment analysis has been performed and visualized using the Metascape tool[89]. -$\log_{10}$(*p*-value) has been used as a representation metric for ranking the enriched terms, and the statistical analysis has been performed using the default parameters[89].

Supplementary Table 1. Experimental codes for all the phenotyping experiments carried out on the BEAN_ADAPT Pv_core2. IPK, Leibniz Institute of Plant Genetics and Crop Plant Research, Gatersleben, Germany; UNIVPM, UNIBAS; Polytechnic University of Marche, Università degli Studi della Basilicata; MPIMP, Max-Planck-Institute of Molecular Plant Physiology, Golm, Germany; SERIDA, Regional Agrifood Research and Development Service, Villaviciosa, Spain.

Experimental code	Responsible institution	Country	Location	Environment	Year	Photoperiod
D1FL6	IPK	Germany	Gatersleben	Field	2016	Long-day
D1FL7	IPK	Germany	Gatersleben	Field	2017	Long-day
I1FL6	UNIVPM, UNIBAS	Italy	Potenza	Field	2016	Long-day
I1FL7	UNIVPM, UNIBAS	Italy	Potenza	Field	2017	Long-day
D2GL6	MPIMP	Germany	Golm	Greenhouse	2016	Long-day
I1GL7	UNIVPM, UNIBAS	Italy	Potenza	Greenhouse	2017	Long-day
S1GL7	SERIDA	Spain	Villaviciosa	Greenhouse	2017	Long-day
D2GS8	MPIMP	Germany	Golm	Greenhouse	2018	Short-day
I1GS6	UNIVPM, UNIBAS	Italy	Potenza	Greenhouse	2016	Short-day
D2Gx7	MPIMP	Germany	Golm	Greenhouse	2017	Intermediate

Supplementary Table 2. Environmental climatic data collected during the growing season in 2016 and 2017 from May to October.

	T min	T mean	T max	Rainfall (mm)	Humidity (%)
D1FL6	6.0	17.2	25.9	151.1	77.3
D1FL7	6.6	16.3	24.5	345.0	79.6

Supplementary Table 3. Environmental climatic data collected during the growing season in 2016 and 2017 from May to October.

	T min	T mean	T max	Rainfall (mm)	Humidity (%)
I1FL6	10.3	10.3	27.6	276.8	51.4
I1FL7	9.6	19.2	29.7	154.6	50.1

Supplementary Table 4. Details on flowering time data for each environment overall American and European accessions and within American and European groups. “No-flowering” refers to the number of accessions that did not reach flowering overall the trial. Broad sense heritability (h^2_B) was calculated either including or not the no-flowering individuals.

ENVIRONMENT	I1GS6	I1FL6	I1GL7	I1FL7	D1FL6	D1FL7	D2GL6	D2GX7	D2GS8	S1FL7
AM AND EU										
MIN	28.30	40.07	27.42	33.98	31.00	40.06	24.26	25.22	30.57	34.00
MAX	50.97	223.68	294.68	297.09	300.00	286.67	298.51	299.11	262.45	300.00
MEAN	37.63	56.64	46.83	55.73	93.49	85.84	66.12	80.31	66.81	91.14
h^2_B (NO-FLOWERING INCLUDED)	85.4%	52.4%	95.9%	95.5%	.	79.7%	98.1%	98.8%	63.1%	.
h^2_B (NO-FLOWERING EXCLUDED)	85.4%	94.2%	75.4%	97.3%	.	73.1%	88.5%	93.7%	72.1%	.
AM										
MIN	29.22	42.13	27.42	34.72	31.00	41.71	26.91	25.22	30.57	46.00
MAX	50.97	223.68	294.68	297.09	300.00	286.67	298.51	299.11	262.45	300.00
MEAN	39.03	65.03	60.90	68.89	129.83	114.65	93.40	122.59	90.23	127.13
No-flowering	0	3	10	5	25	22	17	29	29	27
EU										
MIN	28.30	40.07	27.42	33.98	45.00	40.06	24.26	25.22	30.57	34.00
MAX	47.96	57.23	46.02	58.19	106.00	136.07	68.97	64.73	89.60	69.00
MEAN	36.28	47.79	32.76	42.97	57.92	58.55	39.95	38.04	44.33	54.38
No-flowering	0	0	0	0	0	0	0	0	0	0

Supplementary Table 5. Number and percentage of SNP effects by region.

Type (alphabetical order)	Count	Percent
DOWNSTREAM	1,237,394	20.711%
EXON	131,273	2.197%
INTERGENIC	2,737,645	45.821%
INTRON	422,411	7.07%
SPLICE_SITE_ACCEPTOR	153	0.003%
SPLICE_SITE_DONOR	137	0.002%
SPLICE_SITE_REGION	10,004	0.167%
TRANSCRIPT	6,691	0.112%
UPSTREAM	1,365,988	22.863%
UTR_3_PRIME	35,653	0.597%
UTR_5_PRIME	27,345	0.458%

Supplementary Table 6. Number and percentage of indel effects by region.

Type (alphabetical order)	Count	Percent
DOWNSTREAM	401,306	23.175%
EXON	5,703	0.329%
GENE	1	0%
INTERGENIC	731,084	42.219%
INTRON	122,452	7.071%
SPLICE_SITE_ACCEPTOR	128	0.007%
SPLICE_SITE_DONOR	152	0.009%
SPLICE_SITE_REGION	2,561	0.148%
TRANSCRIPT	2,217	0.128%
UPSTREAM	446,835	25.804%
UTR_3_PRIME	11,434	0.66%
UTR_5_PRIME	7,791	0.45%

Supplementary Table 7. Quantiles describing the distribution of the median length (Kb) of the introgressed blocks inferred for each individual attributed to the EU_AND or the EU_MES groups, tabulated by chromosome.

	EU_AND						EU_MES				
	Min	25%	50%	75%	Max		Min	25%	50%	75%	Max
Chr01	0	20	40	138	1970		20	54	70	113	295
Chr02	0	81	503	900	4770		0	25	38	95	480
Chr03	0	20	40	408	6130		20	80	110	245	1810
Chr04	0	31	90	260	840		30	95	153	225	560
Chr05	0	63	160	320	2330		30	40	50	60	230
Chr06	0	20	165	390	1790		0	30	50	70	560
Chr07	0	20	60	355	2145		0	50	50	90	1130
Chr08	0	25	38	70	4720		20	50	50	93	1524
Chr09	0	0	60	950	8630		0	80	90	135	4780
Chr10	0	20	38	70	1635		20	40	73	106	320
Chr11	0	30	50	168	1440		0	48	60	106	810

Supplementary Table 8. The number of haplotype clusters estimated by the cross-validation procedure implemented in fastPHASE. The analysis was replicated including all accessions (AM+EU) or only American samples (AM).

Chromosome	Hap. Clusters (AM+EU)	Hap. Clusters (AM)
Chr01	20	10
Chr02	20	20
Chr03	20	10-20
Chr04	20	10-20
Chr05	30	10-20
Chr06	10	10-20
Chr07	20	10-20
Chr08	20	10
Chr09	30	10
Chr10	20	10-20
Chr11	20	20-30

Supplementary Table 9. Multivariate correlation analysis between climatic variables and values of membership to different genetic subgroups. For each pairwise correlation, the climatic data at each collection site and the membership values of each accession were used; r, count and *p*-value refer to Pearson's correlation, number of comparisons, and the associated two-sided *p* values, respectively. No multiple comparisons adjustments were made.

Percentage of membership to genetic subgroup	Geospatial or climatic variable	Correlation (r)	Count	*p*-value
P(AM_M1)	bio2	0.19	110	0.050
P(AM_M1)	bio4	0.20	110	0.040
P(AM_M1)	bio7	0.22	110	0.020
P(AM_M2)	bio4	0.19	110	0.051
P(AM_M2)	bio7	0.18	110	0.054
P(AM_A1)	bio2	-0.25	110	0.009
P(AM_A1)	bio5	-0.19	110	0.042
P(AM_A1)	tmax7	-0.19	110	0.042
P(AM_A1)	tmax8	-0.20	110	0.035
P(AM_A3)	Lat	-0.35	110	0.0001
P(AM_A3)	bio1	0.27	110	0.005
P(AM_A3)	bio5	0.22	110	0.019
P(AM_A3)	bio6	0.21	110	0.028
P(AM_A3)	bio10	0.24	110	0.010
P(AM_A3)	bio11	0.24	110	0.012
P(AM_A3)	tmin4	0.26	110	0.006
P(AM_A3)	tmin5	0.25	110	0.008
P(AM_A3)	tmin6	0.22	110	0.020
P(AM_A3)	tmin7	0.23	110	0.016
P(AM_A3)	tmin8	0.25	110	0.008
P(AM_A3)	tmin9	0.26	110	0.007
P(AM_A3)	tmax4	0.23	110	0.016
P(AM_A3)	tmax7	0.20	110	0.032
P(AM_A3)	tmax8	0.24	110	0.011
P(AM_A3)	tmax9	0.29	110	0.002

BIO1 = Annual Mean Temperature
BIO2 = Mean Diurnal Range (Mean of monthly (max temp - min temp))
BIO4 = Temperature Seasonality (standard deviation *100)
BIO7 = Temperature Annual Range (BIO5-BIO6)
BIO5 = Max Temperature of Warmest Month
BIO6 = Min Temperature of Coldest Month
BIO10 = Mean Temperature of Warmest Quarter
BIO11 = Mean Temperature of Coldest Quarter
tmin = minimum temperature; the number indicate the month
tmax = maximum temperature; the number indicate the month
lat = latitude

Supplementary Table 10. Comparisons of the mean values for PC1_FLW between genetic subgroups using Tukey-Kramer HSD. Subgroups showing different letters are significantly different. Significantly different mean values for the flowering have been tested using all the accessions or repeating the analysis considering only European accessions. N.A; not applied, American accessions not included.

	Tukey-Kramer HSD		
Subgroups	Level (all the accessions)	Level (European accessions)	Mean
AM_A1	C	N.A	-1.77
AM_A2	A	N.A	6.76
AM_A3	C	N.A	-0.19
AM_M1	B	N.A	4.58
AM_M2	C	N.A	-1.16
EU_A1	C	B	-1.93
EU_A3	C	A	-1.18
EU_M1	C	A	-1.47
EU_M2	C	A/B	-1.48
EU_MIX	C	A	-1.23

Supplementary Table 11. Comparisons of the mean values for the PC1 on the secondary metabolite profile between genetic subgroups using Tukey-Kramer HSD. Subgroups showing different letters are significantly different.

Tukey-Kramer HSD		
Subgroups	Level	Mean
AM_A1	A	18.89
AM_A2	A	13.22
AM_A3	A	15.68
AM_M1	C	-18.02
AM_M2	D	-29.42
EU_A1	A	18.15
EU_A3	A	14.14
EU_M1	B	-9.21
EU_M2	B/C	-14.84
EU_MIX	B	-2.73

Supplementary Table 12. Genetic diversity (θ_π/bp x 10^4) measured in different groups of accessions according to geographic origin, computed using callable, coding or neutral genomic regions.

	AM_M	AM_M1	AM_M2	EU_M	AM_A	AM_A1	AM_A2	AM_A3	EU_A	AMERICA	EUROPE
Sample size	36	15	21	43	30	11	14	5	71	66	114
CALLABLE	10.7	9.2	6.4	11.3	3.1	1.8	3.0	0.8	7.1	18.7	15.9
CODING	7.5	6.6	5.2	7.5	2.6	1.7	2.5	1.0	4.7	11.2	9.6
NEUTRAL	5.6	4.9	3.6	5.9	1.7	1.1	1.5	0.8	3.9	9.2	8.0

Supplementary Table 13. Genetic diversity (θ_π/bp x 10^4) after the admixture masking (PIND=0.3) process, measured in different groups of accessions according to geographic origin and for each genomic partition. Groups containing masked variants are underlined.

	AM_M	AM_M1	AM_M2	EU_M	AM_A	AM_A1	AM_A2	AM_A3	EU_A	AMERICA	EUROPE
Sample size	36	15	21	43	30	11	14	5	71	66	114
CALLABLE	10.7	9.2	6.4	6.7	3.1	1.8	3.0	0.8	2.0	18.7	15.5
CODING	7.5	6.6	5.2	5.0	2.6	1.7	2.5	1.0	1.8	11.2	9.3
NEUTRAL	5.6	4.9	3.6	4.0	1.7	2.5	1.5	0.8	1.3	9.2	7.8

Supplementary Table 14. Genetic differentiation between groups estimated by the Weir & Cockerham F_{ST} estimator. The F_{ST} is calculated over 10kb not overlapping windows from the admixture-masked dataset. The mean over all distribution (full) or retaining only windows composed by more than 90% of callable regions, is reported. The first and third quartile of the distribution are indicated in brackets.

Compared Groups	Mean F_{ST} (full)	Mean F_{ST} (90% callable wind.)
AMERICA vs EUROPE	0.06 (0.02-0.07)	0.07 (0.03-0.08)
EU_M vs EU_A	0.75 (0.65-0.93)	0.83 (0.75-0.95)
AM_M vs AM_A	0.65 (0.48-0.87)	0.72 (0.59-0.90)
EU_M vs AM_M	0.15 (0.02-0.24)	0.18 (0.04-0.29)
EU_A vs AM_A	0.07 (0.01-0.09)	0.08 (0.01-0.10)

Supplementary Table 15. Number of putative genomic regions target of excess of introgression of Andean alleles into the Mesoamerican gene pool (AND-->MES) or vice versa (MES-->AND), in Europe. The total size (Kb) of the contributing regions is reported in brackets.

Chromosome	Chr. length	Nr AND-->MES	Nr MES-->AND
Chr01	51,433,939	10 (291.6 kb)	0
Chr02	49,670,989	5 (56.1 kb)	0
Chr03	53,438,756	22 (1,095.9 kb)	0
Chr04	48,048,378	18 (882.4 kb)	0
Chr05	40,923,498	13 (86.1 kb)	3 (17.4 kb)
Chr06	31,236,378	13 (278.7 kb)	0
Chr07	40,041,001	19 (453.9 kb)	0
Chr08	63,048,260	14 (336.8 kb)	1 (5.3 kb)
Chr09	38,250,102	6 (189.2 kb)	0
Chr10	44,302,882	6 (143.4 kb)	0
Chr11	53,580,169	5 (174.4 kb)	0

Supplementary Table 16. Regions showing strong inter-chromosomal LD levels that were private of the EU_A group, among the "Extended" set of regions under selection. The genomic position (chromosome, start and end position) is reported for both the regions involved in the inter-chromosomal LD.

Chromosome 1			Chromosome 2		
Chromosome	Start	End	Chromosome	Start	End
5	10102074	10333238	6	1584954	1649852

Supplementary Table 17. Genomic position of the SNPs associated to flowering time and photoperiod sensitivity based on MLMM analysis. The five principal components (PC1-PC5) were obtained from a Principal Component Analysis conducted on flowering time and photoperiod sensitivity data collected in ten different environments (see Supplementary Note 6). The two-sided *p* values were obtained from the MLMM analysis using the mBonf criterion.

Trait	Model	SNP	Chromosome	Position	*p* value
PC2	MLMM	S01_8600671	1	8600671	2.92E-33
PC4	MLMM	S01_40607156	1	40607156	1.02E-67
PC2	MLMM	S01_48049738	1	48049738	2.30E-12
PC3	MLMM	S01_48461310	1	48461310	1.79E-09
PC3	MLMM	S01_49726184	1	49726184	8.46E-11
PC4	MLMM	S02_46715203	2	46715203	6.56E-09
PC3	MLMM	S03_9220681	3	9220681	2.82E-22
PC1	MLMM	S03_38920530	3	38920530	8.39E-43
PC4	MLMM	S04_19617126	4	19617126	1.01E-09
PC3	MLMM	S04_45961875	4	45961875	1.40E-09
PC4	MLMM	S06_18304428	6	18304428	5.74E-37
PC1	MLMM	S06_22375105	6	22375105	1.33E-22
PC1	MLMM	S06_23656275	6	23656275	2.41E-22
PC1	MLMM	S06_23673299	6	23673299	8.32E-36
PC1	MLMM	S07_6699599	7	6699599	1.52E-58
PC2	MLMM	S07_7653755	7	7653755	3.01E-57
PC3	MLMM	S07_32379405	7	32379405	3.13E-12
PC5	MLMM	S08_3456813	8	3456813	2.97E-18
PC4	MLMM	S08_8746566	8	8746566	1.35E-79
PC3	MLMM	S08_51583794	8	51583794	1.19E-15
PC4	MLMM	S08_59927054	8	59927054	2.47E-30
PC4	MLMM	S09_15364000	9	15364000	1.85E-13
PC5	MLMM	S09_15403327	9	15403327	1.23E-10
PC1	MLMM	S09_19302293	9	19302293	8.03E-19
PC3	MLMM	S09_33791935	9	33791935	5.63E-16
PC5	MLMM	S10_4848788	10	4848788	7.12E-16
PC4	MLMM	S10_33903375	10	33903375	4.08E-20
PC1	MLMM	S11_8828512	11	8828512	5.12E-16
PC1	MLMM	S11_11967671	11	11967671	6.65E-17

Supplementary references

1. Cortinovis, G., Di Vittori, V., Bellucci, E., Bitocchi, E. & Papa, R. Adaptation to novel environments during crop diversification. *Curr. Opin. Plant Biol.* **56**, 218–222 (2020).
2. Brush, S.B. In Situ Conservation of Landraces in Centers of Crop Diversity. *Crop Sci.* **35**, 346-354 (1995).
3. Perales, R.H., Brush, S.B. & Qualset, C.O. Dynamic Management of Maize Landraces in Central Mexico. *Econ. Bot.* **57**, 21-34 (2003).
4. Rodriguez, M., Rau, D., Papa, R. & Attene, G. Genotype by environment interactions in barley (Hordeum vulgare L.): different responses of landraces, recombinant inbred lines and varieties to Mediterranean environment. *Euphytica* **163**, 231–247 (2008)
5. Bitocchi, E. *et al*. Introgression from modern hybrid varieties into landrace populations of maize (*Zea mays* ssp. *mays* L.) in central Italy. *Mol. Ecol.* **18**, 603-621 (2009).
6. Bellucci, E. *et al*. Population structure of barley landrace populations and gene-flow with modern varieties. *PLoS ONE* **8**, e83891 (2013).
7. Dwivedi, S.L. *et al*. Landrace germplasm for improving yield and abiotic stress adaptation. *Trends Plant Sci*. **21**, 31-42 (2016).
8. Russell, J. *et al*. Exome sequencing of geographically diverse barley landraces and wild relatives gives insights into environmental adaptation. *Nat. Genet*. **48**, 1024–1030 (2016).
9. Perez de Souza, L. *et al*. Multi-tissue integration of transcriptomic and specialized metabolite profiling provides tools for assessing the common bean (*Phaseolus vulgaris*) metabolome. *Plant J*. **97**, 1132-1153 (2019).
10. Wei, T. & Simko, V. R package "corrplot": Visualization of a Correlation Matrix (Version 0.84). Available from https://github.com/taiyun/corrplot (2017).
11. Peterson, B.G. *et al*. PerformanceAnalytics: Econometric Tools for Performance and Risk Analysis (Version 2.0.4). Available from https://github.com/braverock/PerformanceAnalytics (2020).
12. Dahl, A. *et al*.A multiple-phenotype imputation method for genetic studies. *Nat. genet.* **47**, 466 (2016).

13. Schmutz, J. *et al*. A reference genome for common bean and genome-wide analysis of dual domestications. *Nat. Genet*. **46**, 707-713 (2014).

14. Li, H. Aligning sequence reads, clone sequences and assembly contigs with BWA-MEM. *arXiv* 1303.3997 [q-bio.GN] (2013).

15. Li, H. *et al.* The Sequence Alignment/Map format and SAMtools. *Bioinformatics* **25**, 2078-2079 (2009).

16. Li, H. A statistical framework for SNP calling, mutation discovery, association mapping and population genetical parameter estimation from sequencing data. *Bioinformatics* **27**, 2987-2993 (2011).

17. McKenna, A. *et al*. The Genome Analysis Toolkit: A MapReduce framework for analyzing next-generation DNA sequencing data. *Genome Res*. **20**, 1297-1303 (2010).

18. Van der Auwera, G.A. *et al*. From FastQ data to high confidence variant calls: the Genome Analysis Toolkit best practices pipeline. *Curr. Protoc. Bioinformatics*. **43**, 11.10.1-11.10.33 (2013).

19. Cleary, J.G. *et al*. Comparing Variant Call Files for Performance Benchmarking of Next-Generation Sequencing Variant Calling Pipelines. *bioRxiv* **023754**, doi: https://doi.org/10.1101/023754 (2015).

20. Cingolani, P. *et al*. A program for annotating and predicting the effects of single nucleotide polymorphisms, SnpEff. *Fly* **6**, 80-92 (2012).

21. Wang, X.H. *et al.* CNVcaller: highly efficient and widely applicable software for detecting copy number variations in large populations. *Gigascience* **6**, 1-12 (2017).

22. Chaisson, M.J. & Tesler, G. Mapping single molecule sequencing reads using basic local alignment with successive refinement (BLASR): application and theory. *Bmc Bioinform*. **13**, 238 (2012).

23. Danecek, P. *et al*. The variant call format and VCFtools. *Bioinformatics* **27**, 2156-8 (2011).

24. Browning, S. R. & Browning, B. L. Rapid and accurate haplotype phasing and missing data inference for whole genome association studies by use of localized haplotype clustering. *Am. J. Hum. Genet.* **81**, 1084-1097 (2007).

25. Alexander, D.H., Novembre, J. & Lange, K. Fast model-based estimation of ancestry in unrelated individuals. *Genome Res.* **19**, 1655-64 (2009).

26. Rossi, M. *et al.* Linkage disequilibrium and population structure in wild and domesticated populations of *Phaseolus vulgaris* L. *Evol. Appl*. **2**, 504–522 (2009).

27. Bitocchi, E. *et al*. Mesoamerican origin of the common bean (Phaseolus vulgaris L.) is revealed by sequence data. *Proc. Natl. Acad. Sci. USA* **109**, E788–E796 (2012).

28. Bitocchi, E. *et al*. Molecular analysis of the parallel domestication of the common bean (*Phaseolus vulgaris*) in Mesoamerica and the Andes. *New Phytol.* **197**, 300–313 (2013).

29. Corander, J., Waldmann, P. & Sillanpää, M.J. Bayesian analysis of genetic differentiation between populations. *Genetics* **163**, 367-74 (2003).

30. Corander, J., Marttinen, P., Sirén, J. & Tang, J. Enhanced Bayesian modelling in BAPS software for learning genetic structures of populations. *BMC Bioinform.* **9**, 539 (2008).

31. Kumar, S., Stecher, G., Li, M., Knyaz, C. & Tamura, K. MEGA X: Molecular Evolutionary Genetics Analysis across Computing Platforms. *Mol. Biol. Evol.* **35**, 1547–49 (2018).

32. Nei, M. & Kumar, S. Molecular Evolution and Phylogenet-ics. Oxford University Press, New York (2000).

33. Wu, J. *et al*. Resequencing of 683 common bean genotypes identifies yield component trait associations across a north–south cline. *Nat. Genet.* **52**, 118–125 (2020).

34. Purcell, S. *et al*. PLINK: a tool set for whole-genome association and population-based linkage analyses. *Am. J. Hum. Genet*. **81**, 559-75 (2007).

35. Schliep, K.P. phangorn: phylogenetic analysis in R. *Bioinformatics* **27**, 592-3 (2011).

36. Huson, D.H. & Bryant, D. Application of phylogenetic networks in evolutionary studies. *Mol. Biol. Evol.* **23**, 254-267 (2006).

37. Bryant, D. & Moulton, V. Neighbor-net: an agglomerative method for the construction of phylogenetic networks. *Mol. Biol. Evol*. **21**, 255-65 (2004).

38. Lawson, D.J., Hellenthal, G., Myers, S. & Falush, D. Inference of population structure using dense haplotype data. *PLoS Genet.* **8**, e1002453 (2012).

39. Quinlan, A. R. & Hall, I. M. BEDTools: a flexible suite of utilities for comparing genomic features. *Bioinformatics* **26**, 841–842 (2010).

40. Janzen, T. & Miró Pina, V. Estimating the time since admixture from phased and unphased molecular data. *Mol. Ecol. Resour.* **22**, 908– 926 (2022).

41. Blair, M.W. *et al*. Uneven recombination rate and linkage disequilibrium across a reference SNP map for common bean (*Phaseolus vulgaris* L.). *PLoS ONE* **13**, e0189597 (2018).

42. Fariello, M.I., Boitard, S., Naya, H., SanCristobal, M. & Servin, B. Detecting signatures of selection through haplotype differentiation among hierarchically structured populations. *Genetics* **193**, 929-41 (2013).

43. Scheet, P. & Stephens, M. A fast and flexible statistical model for large-scale population genotype data: applications to inferring missing genotypes and haplotypic phase. *Am. J. Hum. Genet.* **78**, 629-44 (2006).

44. Hijmans, R. J., Cameron, S. E., Parra, J.L., Jones, P. G. & Jarvis, A. Very high resolution interpolated climate surfaces for global land areas. *Int. J. Climatol*. **25**, 1965–1978 (2005).

45. Oksanen, J. *et al*. vegan: Community Ecology Package. R package version 2.5-7. https://CRAN.R-project.org/package=vegan (2020).

46. Wallace, L., Arkwazee, H., Vining, K. & Myers, J.R. Genetic diversity within snap beans and their relation to dry beans. *Genes* **9**, 587 (2018).

47. Whitlock, M.C. Evolutionary inference from QST. *Mol.* **17**, 1885-1896 (2008).

48. Beleggia, R. *et al*. Evolutionary metabolomics reveals domestication-associated changes in tetraploid wheat kernels. *Mol. Biol. Evol.* **33**,1740-53 (2016).

49. Tajima, F. Evolutionary relationship of DNA sequences in finite populations. *Genetics* **105**, 437-60 (1983).

50. Vigouroux, Y. *et al.* Identifying genes of agronomic importance in maize by screening microsatellites for evidence of selection during domestication. *Proc. Natl. Acad. Sci. USA* **99**, 9650-5 (2002).

51. Zhang, C., Dong, S.S., Xu, J.Y., He, W.M. & Yang, T.L. PopLDdecay: a fast and effective tool for linkage disequilibrium decay analysis based on variant call format files. *Bioinformatics* **35**, 1786-1788 (2019).

52. Weir, B.S. & Cockerham, C.C. Estimating F-statistics for the analysis of population structure. *Evolution,* **38**, 1358-1370 (1984).

53. Zhang, H., Meltzer, P. & Davis, S. RCircos: an R package for Circos 2D track plots. *BMC bioinformatics* **14**, 244 (2013).

54. Browning, B. L. & Browning, S. R. Genotype imputation with millions of reference samples. *Am. J. Hum. Genet*. **98**, 116-126 (2016).

55. Privé, F., Aschard, H., Ziyatdinov, A. & Blum, M. G. Efficient analysis of large-scale genome-wide data with two R packages: bigstatsr and bigsnpr. *Bioinformatics* **34**, 2781-2787 (2018).

56. Yu, J. *et al.* A unified mixed-model method for association mapping that accounts for multiple levels of relatedness. *Nat. genet.* **38**, 2 (2006).

57. Zhou, X. & Stephens, M. Genome-wide efficient mixed-model analysis for association studies. *Nat. genet.* **44**, 821 (2012).

58. Cui, Y., Zhang, F. & Zhou, Y. The application of multi-locus GWAS for the detection of salt-tolerance loci in rice. *Front. Plant Sci.* **9** (2018).

59. Segura, V. *et al*. An efficient multi-locus mixed-model approach for genome-wide association studies in structured populations. *Nat. Genet.* **44**, 825 (2012).

60. Wang, S.B. *et al.* Improving power and accuracy of genome-wide association studies via a multi-locus mixed linear model methodology. *Sci. Rep.* **6**, 19444 (2016).

61. Fusi, N., Lippert, C., Lawrence, N. D. & Stegle, O. Warped linear mixed models for the genetic analysis of transformed phenotypes. *Nat. commun*. **5**, 4890 (2014).

62. Ma, C. Statistical Methods for Low-frequency and Rare Genetic Variants (University of Michigan, 2014).

63. Wu, Y., Zheng, Z., Visscher, P. M. & Yang, J. Quantifying the mapping precision of genome-wide association studies using whole-genome sequencing data. *Genome biol.* **18**, 86 (2017).

64. Goh, L. & Yap, V. B. Effects of normalization on quantitative traits in association test. *BMC Bioinform*. **10**, 415 (2009).

65. Beasley, T. M., Erickson, S. & Allison, D. B. Rank-based inverse normal transformations are increasingly used, but are they merited? *Behav. Genet*. **39**, 580 (2009).

66. Bůžková, P. Linear regression in genetic association studies. *PLoS ONE* **8**, e56976 (2013).

67. Liu, W. *et al*.Genome-wide association mapping for seedling and field resistance to *Puccinia striiformis* f. sp. *tritici* in elite durum wheat. *Theor. Appl. Genet*. **130**, 649-667 (2017).

68. Kobayashi, Y. & Weigel, D. Move on up, it's time for change—mobile signals controlling photoperiod-dependent flowering. *Genes Dev*. **21**, 2371-2384 (2007).

69. Buckler, E. S. *et al*. The genetic architecture of maize flowering time. *Science* **325**, 714-718 (2009).

70. Chang, F. *et al*. Genome-wide association studies for dynamic plant height and number of nodes on the main stem in summer sowing soybeans. *Front. Plant Sci.* **9**, 1184 (2018).

71. He, L. *et al*. Genome-wide association studies for pasmo resistance in flax (*Linum usitatissimum* L.). *Front. Plant Sci.* **9**, 1982 (2019).

72. Li, C., Fu, Y., Sun, R., Wang, Y. & Wang, Q. Single-locus and multi-locus genome-wide association studies in the genetic dissection of fiber quality traits in upland cotton (*Gossypium hirsutum* L.). *Front. Plant Sci.* **9**, 1083 (2018).

73. Xu, Y. *et al.* Genome-wide association mapping of starch pasting properties in maize using single-locus and multi-locus models. *Front. Plant Sci.* **9**, 1311 (2018).

74. Zhang, Y.M., Jia, Z. & Dunwell, J.M. The applications of new multi-locus GWAS methodologies in the genetic dissection of complex traits. *Front. Plant Sci.* **10**, 100 (2019).

75. Kwak, M., Velasco, D. & Gepts, P. Mapping homologous sequences for determinacy and photoperiod sensitivity in common bean (Phaseolus vulgaris). *J. Hered* **99**, 283–291(2008).

76. Repinski, S.L., Kwak, M. & Gepts, P. The common bean growth habit gene PvTFL1y is a functional homolog of Arabidopsis TFL1. *Theor. Appl. Genet*. **124**, 1539–1547 (2012).

77. Koinange, E.M.K., Singh, S.P. & Gepts, P. Genetic Control of the Domestication Syndrome in Common Bean. *Crop Sci*. **36**, 1037-1045 (1996).

78. Cichy, K. *et al.* A Phaseolus vulgaris diversity panel for Andean bean improvement. *Crop Sci.* **55**, 2149–2160 (2015).

79. Moghaddam, S.M. *et al*. Genome-Wide Association Study Identifies Candidate Loci Underlying Agronomic Traits in a Middle American Diversity Panel of Common Bean. *Plant Genome* **9**, plantgenome2016.02.0012 (2016).

80. Oladzad, A. *et al.* Single and Multi-trait GWAS Identify Genetic Factors Associated with Production Traits in Common Bean Under Abiotic Stress Environments. *G3: Genes Genomes Genet.* **9**, 1881-1892 (2019).

81. Raggi, L., Tissi, C., Mazzucato, A. & Negri, V. Molecular polymorphism related to flowering trait variation in a Phaseolus vulgaris L. collection. *Plant Sci.* **215-216**, 180-189 (2014).

82. Weller, J. *et al.* Parallel origins of photoperiod adaptation following dual domestications of common bean. *J. Exp. Bot.***70**, 1209–1219 (2019).

83. Ugwuanyi, S., Udengwu, O.S., Snowdon, R.J. & Obermeier, C. Novel candidate loci for morpho-agronomic and seed quality traits detected by targeted genotyping-by-sequencing in common bean. *Front. Plant Sci.* **13**, 1014282 (2022).

84. Wallace, D.H., Yourstone, K.S., Masaya, P.N. & Zobel, R.W. Photoperiod gene control over partitioning between reproductive and vegetative growth. *Theor. Appl. Genet.* **86**, 6–16 (1993).

85. Watanabe, S., Harada, K. & Abe, J. Genetic and molecular bases of photoperiod responses of flowering in soybean. *Breed Sci.* **61**, 531-43 (2012).

86. Kamfwa, K., Cichy, K.A. & Kelly, J.D. Genome-wide association study of agronomic traits in common bean. *Plant Genome* **8**, plantgenome2014.09.0059 (2015).

87. Emms, D.M. & Kelly, S. OrthoFinder: phylogenetic orthology inference for comparative genomics. *Genome Biol.* **20**, 238 (2019).

88. Di Vittori, V. *et al.* Pod indehiscence in common bean is associated with the fine regulation of PvMYB26. *J. Exp. Bot*. **72**, 1617–1633 (2021).

89. Zhou, Y. *et al.* Metascape provides a biologist-oriented resource for the analysis of systems-level datasets. *Nat. Commun.* **10**, 1523 (2019).

90. Schaffer, R. *et al*. The late elongated hypocotyl mutation of Arabidopsis disrupts circadian rhythms and the photoperiodic control of flowering. *Cell* **93**, 1219-29 (1998).

91. Adams, S., Manfield, I., Stockley, P. & Carré, I.A. Revised morning loops of the Arabidopsis circadian clock based on analyses of direct regulatory interactions. *Plos One* **10**, e0143943 (2015).

92. Kaldis, A., Kousidis, P., Kesanopoulos, K. & Prombona, A. Light and circadian regulation in the expression of LHY and Lhcb genes in Phaseolus vulgaris. *Plant Mol. Biol*. **52**, 981–997 (2003).

93. Song, Y.H., Ito, S. & Imaizumi, T. Similarities in the circadian clock and photoperiodism in plants. *Curr. Opin. Plant Biol*. **13**, 594–603 (2010).

94. Yang, X. *et al.* Transcriptome analysis to identify putative genes involved in flowering time under different photoperiods in 'Hong jin gou' common bean. *J. Amer. Soc. Hort. Sci.* **144**, 274–279 (2019).

95. Hazen, S.P. *et al*. LUX ARRHYTHMO encodes a Myb domain protein essential for circadian rhythms. *Proc. Natl. Acad. Sci. USA*. **102**, 10387-10392 (2005).

96. Helfer, A. *et al*. LUX ARRHYTHMO encodes a nighttime repressor of circadian gene expression in the Arabidopsis core clock. *Curr. Biol*. **21**, 126-133 (2011).

97. Zhang, C. *et al*. LUX ARRHYTHMO mediates crosstalk between the circadian clock and defense in Arabidopsis. *Nat. Commun.* **10**, 2543 (2019).

98. Gendron, J.M. *et al.* Arabidopsis circadian clock protein, TOC1, is a DNA-binding transcription factor. *Proc. Natl. Acad. Sci. U.S.A* **109**, 3167-3172 (2012).

99. Kamioka, M. *et al*. Direct repression of evening genes by CIRCADIAN CLOCK-ASSOCIATED1 in the Arabidopsis circadian clock. *Plant Cell* **28**, 696–711 (2016).

100. Heo, J.B., Sung, S. & Assmann, S.M. Ca2+ dependent GTPase, extra-large G protein 2 (XLG2), promotes activation of DNA binding protein related to vernalization 1 leading to activation of floral integrator genes and early flowering in Arabidopsis. *J. Biol. Chem*. **287**, 8242 8253 (2012).

101. Levy, Y.Y., Mesnage, S., Mylne, J.S., Gendall, A.R. & Dean, C. Multiple roles of Arabidopsis VRN1 in vernalization and flowering time control. *Science* **297**, 243-246 (2002).

102. Searle, I. *et al*. The transcription factor FLC confers a flowering response to vernalization by repressing meristem competence and systemic signaling in Arabidopsis. *Genes Dev*. **20**, 898-912 (2006).

103. Chen, D., Molitor, A., Liu, C. & Shen, W.H. The Arabidopsis PRC1-like ring-finger proteins are necessary for repression of embryonic traits during vegetative growth. *Cell Res.* **20**, 1332–1344 (2010).

104. Picó, S., Ortiz-Marchena, M.I., Merini, W. & Calonje, M. Deciphering the role of POLYCOMB REPRESSIVE COMPLEX1 variants in regulating the acquisition of flowering competence in Arabidopsis. *Plant Physiol*. **168**, 1286-1297 (2015).

105. Qin, F. *et al*. ArabidopsisDREB2A-interacting proteins function as RINGE3 ligases and negatively regulate plant drought stress-responsive gene expression. *Plant Cell*. **20**, 1693-1707 (2008).

106. Merini, W. *et al*. The Arabidopsis polycomb repressive complex 1 (PRC1) components AtBMI1A, B, and C impact gene networks throughout all stages of plant development. *Plant Physiol.* **173**, 627-641 (2017).

107. Trevaskis, B., Hemming, M.N., Peacock, W.J. & Dennis, E.S. HvVRN2 responds to daylength, whereas HvVRN1 is regulated by vernalization and developmental status. *Plant Physiol*. **140**, 1397–1405 (2006).

108. Yan, L. *et al*. Positional cloning of the wheat vernalization gene VRN1. *Proc. Natl. Acad. Sci. USA* **100**, 6263–6268 (2003).

109. Xu, L. *et al.* The E2 ubiquitin-conjugating enzymes, AtUBC1 and AtUBC2, play redundant roles and are involved in activation of FLC expression and repression of flowering in Arabidopsis thaliana. *Plant J*. **57**, 279-88 (2009).

110. Kim, D.W. Current understanding of flowering pathways in plants: focusing on the vernalization pathway in Arabidopsis and several vegetable crop plants. *Hortic. Environ. Biotechnol.* **61**, 209–227 (2020).

111. Lee, I. *et al*. Isolation of LUMINIDEPENDENS: a gene involved in the control of flowering time in Arabidopsis. *Plant Cell*. **6**, 75-83 (1994).

112. Aukerman, M.J., Lee, I., Weigel, D. & Amasino, R.M. The Arabidopsis flowering time gene LUMINIDEPENDENS is expressed primarily in regions of cell proliferation and encodes a nuclear protein that regulates LEAFY expression. *Plant J*. **18**, 195-203 (1999).

113. Yamaguchi, S. Gibberellin metabolism and its regulation. *Annu. Rev. Plant. Biol*. **59**, 225-251 (2008).

114. Richter, R., Bastakis, E. & Schwechheimer, C. Cross-repressive interactions between SOC1 and the GATAs GNC and GNL/CGA1 in the control of greening, cold tolerance, and flowering time in Arabidopsis. *Plant Physiol*. **162**, 1992-2004 (2013).

115. Richter, R., Behringer, C., Müller, I.K. & Schwechheimer, C. The GATA-type transcription factors GNC and GNL/CGA1 repress gibberellin signaling downstream from DELLA proteins and PHYTOCHROME-INTERACTING FACTORS. *Genes Dev*. **24**, 2093-2104 (2010).

116. Hudson, D. et al. GNC and CGA1 modulate chlorophyll biosynthesis and glutamate synthase (GLU1/Fd-GOGAT) expression in Arabidopsis. *PLoS ONE* **6**, e26765 (2011).

117. Chiang, Y.H. *et al*. Functional characterization of the GATA transcription factors GNC and CGA1 reveals their key role in chloroplast development, growth, and division in Arabidopsis. *Plant Physiol*. **160**, 332-48 (2012).

118. King, R.W., Hisamatsu, T., Goldschmidt, E.E. & Blundell, C. The nature of floral signals in Arabidopsis. I. Photosynthesis and a far-red photoresponse independently regulate flowering by increasing expression of *FLOWERING LOCUS T* (*FT*). *J. Exp. Bot.* **59**, 3811–3820 (2008).

119. Seo, P.J., Ryu, J., Kang, S.K. & Park, C.M. Modulation of sugar metabolism by an INDETERMINATE DOMAIN transcription factor contributes to photoperiodic flowering in Arabidopsis. *Plant J.* **65**, 418-429 (2011).

120. Levesque, M.P. *et al*. Whole-genome analysis of the SHORT-ROOT developmental pathway in Arabidopsis. *PLOS Biol*. **4**, e249 (2006).

121. Helariutta, Y. *et al*. The SHORT-ROOT gene controls radial patterning of the Arabidopsis root through radial signaling. *Cell* **101**, 555-567 (2000).

122. Sozzani, R. *et al*. Spatiotemporal regulation of cell-cycle genes by SHORTROOT links patterning and growth. *Nature* **466**, 128–132 (2010).

123. Lucas, M. *et al.* Short Root regulates primary, lateral, and adventitious root development in Arabidopsis. *Plant Physiol.* **155**, 384 398 (2011).

124. Zhang, Y. e*t al.* Arabidopsis VILLIN4 is involved in root hair growth through regulating actin organization in a Ca2+-dependent manner. *New Phytol*. **190**, 667-682 (2011).

125. Zhao, Y. Auxin biosynthesis and its role in plant development. *Annu. Rev. Plant Biol*. **61**, 49-64 (2010).

126. Ruegger, M. *et al.* The TIR1 protein of Arabidopsis functions in auxin response and is related to human SKP2 and yeast grr1p. *Genes Dev*. **12**, 198-207 (1998).

127. Yen, M.R. *et al*. Deubiquitinating enzyme OTU5 contributes to DNA methylation patterns and is critical for phosphate nutrition signals. *Plant Physiol.* **175**, 1826-1838 (2017).

128. Suen, D.F. *et al*. The Deubiquitinase OTU5 regulates root responses to phosphate starvation. *Plant Physiol.* **176**, 2441-2455 (2018).

129. Pinon, V., Ravanel, S., Douce, R. & Alban, C. Biotin synthesis in plants. The first committed step of the pathway is catalyzed by a cytosolic 7-keto-8-aminopelargonic acid synthase. *Plant Physiol.* **139**, 1666-1676 (2005).

130. Li, J., Brader, G., Helenius, E., Kariola, T. & Palva, E.T. Biotin deficiency causes spontaneous cell death and activation of defense signalling. *Plant J.* **70**, 315-326 (2012).

131. Xuan, Y.H. *et al.* Functional role of oligomerization for bacterial and plant SWEET sugar transporter family. *Proc. Natl. Acad. Sci. USA* **110**, E3685-E3694 (2013).

132. Gautam, T. *et al*. Further studies on sugar transporter (*SWEET*) genes in wheat (*Triticum aestivum* L.). *Mol. Biol. Rep*. **46**, 2327–2353 (2019).

133. Zhang, W. *et al.* Genome-wide characterization and expression profiling of SWEET genes in cabbage (*Brassica oleracea* var. *capitata* L.) reveal their roles in chilling and clubroot disease responses. *BMC Genomics* **20**, 93 (2019).

134. Guo, J. *et al*. COE 1 and GUN1 regulate the adaptation of plants to high light stress. Biochem. Biophys. *Res. Commun.* **521**, 184-189 (2020).

135. Whetten, R. & Sederof, R. Lignin biosynthesis. *Plant Cell* **7**, 1001-1013 (1995)

136. Lattanzio, V., Lattanzio, V.M.T. & Cardinali, A. Role of phenolics in the resistance mechanisms of plants against fungal pathogens and insects. *Phytochemistry: Advances in Research*, 23-67 (2006).

137. Riaz, U. *et al*. Prospective roles and mechanisms of caffeic acid in counter plant stress: a mini review. *Pak. J. Agric. Sci.* **32**, 8-19 (2019).

138. Hiratsuka, K., Wu, X., Fukuzawa, H. & Chua, N.H. Molecular dissection of GT-1 from Arabidopsis. *Plant Cell*. **6**, 1805-13 (1994).

139. Samol, I. *et al.* Identification of a photosystem II phosphatase involved in light acclimation in Arabidopsis. *Plant Cell*. **24**, 2596-609 (2012).

140. Cui, X. *et al.* Ubiquitin-specific proteases UBP12 and UBP13 act in circadian clock and photoperiodic flowering regulation in Arabidopsis. *Plant Physiol*. **162**, 897-906 (2013).

141. Shim, J.S., Kubota, A. & Imaizumi, T. Circadian clock and photoperiodic flowering in Arabidopsis: CONSTANS is a hub for signal integration. *Plant Physiol*. **173**, 5-15 (2017).

142. Putterill, J., Robson, F., Lee, K., Simon, R & Coupland, G. The CONSTANS gene of Arabidopsis promotes flowering and encodes a protein showing similarities to zinc finger transcription factors. *Cell* **80**, 847-857 (1995).

143. Rosas, U. *et al.* Variation in Arabidopsis flowering time associated with cis-regulatory variation in CONSTANS. *Nat. Commun*. **5**, 3651 (2014).

144. Wu, M. *et al.* PIL5 represses floral transition in Arabidopsis under long day conditions. *Biochem. Biophys. Res. Commun*. **499**, 513 518 (2018).

145. Liu, Y., Li, X., Li, K., Liu, H. & Lin, C. Multiple bHLH proteins form heterodimers to mediate CRY2-dependent regulation of flowering-time in Arabidopsis. *PLoS Genet*. **9**, e1003861 (2013).

146. Christians, M.J., Gingerich, D.J., Hua, Z., Lauer, T.D. & Vierstra, R.D. The light-response BTB1 and BTB2 proteins assemble nuclear ubiquitin ligases that modify phytochrome B and D signaling in Arabidopsis. *Plant Physiol*. **160**, 118-34 (2012).

147. Shi, H., Wei, Y., Wang, Q., Reiter, R.J. & He, C. Melatonin mediates the stabilization of DELLA proteins to repress the floral transition in Arabidopsis. *J. Pineal Res.* **60**, 373-379 (2016).

148. Jasinski, S. *et al*. KNOX action in Arabidopsis is mediated by coordinate regulation of cytokinin and gibberellin activities. *Curr. Biol.* **15**, 1560-5 (2005).

149. Shannon, S. & Meeks-Wagner, D.R. A Mutation in the Arabidopsis TFL1 gene affects inflorescence meristem development. *Plant Cell*. **3**, 877-892 (1991).

150. Kobayashi, Y., Kaya, H., Goto, K., Iwabuchi, M. & Araki, T. A pair of related genes with antagonistic roles in mediating flowering signals. *Science* **286**, 1960-1962 (1999).

151. Imura, Y. *et al.* CRYPTIC PRECOCIOUS/MED12 is a novel flowering regulator with multiple target steps in Arabidopsis. *Plant Cell Physiol.* **53**, 287-303 (2012).

152. Ferràndiz, C., Gu, Q., Martienssen, R. & Yanofsky, M.F. Redundant regulation of meristem identity and plant architecture by *FRUITFULL, APETALA1* and *CAULIFLOWER. Development* **127**, 725-734 (2000).

153. Saddic, L.A. *et al*. The LEAFY target LMI1 is a meristem identity regulator and acts together with *LEAFY* to regulate expression of *CAULIFLOWER*. *Development* **133**, 1673-1682 (2006).

154. Sivitz, A.B. *et al.* Arabidopsis sucrose transporter AtSUC9. High affinity transport activity, intragenic control of expression, and early flowering mutant phenotype. *Plant Physiol*. **143**, 188 198 (2007).

155. Zhan, X. *et al.* Arabidopsis proline-rich protein important for development and abiotic stress tolerance is involved in microRNA biogenesis. *Proc. Natl. Acad. Sci. USA* **109**, 18198-18203 (2012).

156. Marshall, C.M., Tartaglio, V., Duarte, M. & Harmon, F.G. The Arabidopsis sickle mutant exhibits altered circadian clock responses to cool temperatures and temperature-dependent alternative splicing. *Plant Cell*. **28**, 2560-2575 (2016).

157. Daniel, X., Lacomme, C., Morel, J.B. & Roby, D. A novel myb oncogene homologue in Arabidopsis thaliana related to hypersensitive cell death. *Plant J.* **20**, 57–66 (1999).

158. Liu, L. *et al*. Elevated levels of MYB30 in the phloem accelerate flowering in Arabidopsis through the regulation of FLOWERING LOCUS T. *PLoS ONE* **9**, e89799 (2014).

159. Mabuchi, K. *et al*. MYB30 links ROS signaling, root cell elongation, and plant immune responses. *Proc. Natl. Acad. Sci. USA* **115**, E4710-E4719 (2018).

160. Liu, Z. *et al*. A conserved cytochrome P450 evolved in seed plants regulates flower maturation. *Mol Plant*. **8**, 1751-65 (2015).

161. Casamitjana-Martínez, E. *et al*. Root-specific CLE19 overexpression and the sol1/2 suppressors implicate a CLV-like pathway in the control of Arabidopsis root meristem maintenance. *Curr. Biol.* **13**, 1435-1441 (2003).

162. Wen, J., Li, J. & Walker, J.C. Overexpression of a serine carboxypeptidase increases carpel number and seed production in Arabidopsis thaliana. *Food Energy Secur.* **1**, 61–69 (2012).

163. Morrone, D., Chen, X., Coates, R.M. & Peters, R.J. Characterization of the kaurene oxidase CYP701A3, a multifunctional cytochrome P450 from gibberellin biosynthesis. *Biochem. J.* **431**, 337-44 (2010).

164. Somssich, M., Il Je, B., Simon, R. & Jackson, D. *CLAVATA-WUSCHEL* signaling in the shoot meristem. *Development* **143**, 3238-3248 (2016).

165. Kwak, M., Toro, O., Debouck, D.G. & Gepts, P. Multiple Origins of the Determinate Growth Habit in Domesticated Common Bean (*Phaseolus vulgaris*). *Ann. Bot.* **110**, 1573–1580 (2022).

166. Giordani, W. Dissecting the genetic architecture of quantitative traits in common bean (*Phaseolus vulgaris* L.): response to root-knot nematode infection and seed morphology. (Universidade de São Paulo, 2021).

167. Boubakri, H. *et al*. Genome-wide identification, characterization and expression analysis of glutaredoxin gene family (Grxs) in *Phaseolus vulgaris*. *Gene* **833**, 146591 (2022).

168. Tan, Q.K. & Irish, V.F. The Arabidopsis zinc finger-homeodomain genes encode proteins with unique biochemical properties that are coordinately expressed during floral development. *Plant Physiol*. **140**, 1095-108 (2006).

169. Conti, L. & Bradley, D. *TERMINAL FLOWER1* is a mobile signal controlling Arabidopsis architecture. *Plant Cell.* **19**, 767-78 (2007).

170. Baumann, K. *et al*. Changing the spatial pattern of TFL1 expression reveals its key role in the shoot meristem in controlling Arabidopsis flowering architecture. *J. Exp. Bot*. **66**, 4769-80 (2015).

171. Chen, Z.H. *et al*. Regulation of auxin response by miR393-targeted transport inhibitor response protein 1 is involved in normal development in Arabidopsis. *Plant Mol. Biol.* **77**, 619-29 (2011).

172. Mata-Pérez, C. & Spoel, S.H. Thioredoxin-mediated redox signalling in plant immunity. *Plant Sci.* **279**, 27-33 (2019).

173. Motohashi, R. *et al*. Chloroplast ribosome release factor 1 (AtcpRF1) is essential for chloroplast development. *Plant Mol. Biol.* **64**, 481–497 (2007).

174. Wen, C.K. & Chang, C. Arabidopsis RGL1 encodes a negative regulator of gibberellin responses. *Plant Cell*. **14**, 87-100 (2002).

175. Chen, M. *et al.* Removal of DELLA repression promotes leaf senescence in Arabidopsis. *Plant Sci*. **219-220**, 26-34 (2014).

176. Wang, W. *et al*. The six conserved serine/threonine sites of REPRESSOR OF ga1-3 protein are important for its functionality and stability in gibberellin signalling in Arabidopsis. *Planta* **240**, 763-79 (2014).

177. Stamm, P. *et al.* Insights into the molecular mechanism of RGL2-mediated inhibition of seed germination in *Arabidopsis thaliana*. *BMC Plant Biol.* **12**, 179 (2012).

178. Christians, M.J., Robles, L.M., Zeller, S.M. & Larsen, P.B. The eer5 mutation, which affects a novel proteasome-related subunit, indicates a prominent role for the COP9 signalosome in resetting the ethylene-signaling pathway in Arabidopsis. *Plant J.* **55**, 467-77 (2008).

179. Sato, R., Ito, H. & Tanaka, A. Chlorophyll b degradation by chlorophyll b reductase under high-light conditions. *Photosynth. Res*. **126**, 249-59 (2015).

180. Cock, J.M. & McCormick, S. A large family of genes that share homology with CLAVATA3. *Plant Physiol.* **126**, 939-42 (2001).

181. Zhang, H. *et al*. Arabidopsis VILLIN5, an actin filament bundling and severing protein, is necessary for normal pollen tube growth. *Plant Cell* **22**, 2749-67 (2010).

182. Dai, S., Wei, X. & Pei, L. Thompson RL, Liu Y, Heard JE, Ruff TG, Beachy RN. *BROTHER OF LUX ARRHYTHMO* is a component of the Arabidopsis circadian clock. *Plant Cell* **23**, 961-72 (2011).

183. Yuan, S. *et al.* Genome-wide identification and classification of soybean C2H2 Zinc finger proteins and their expression analysis in legume-rhizobium symbiosis. *Front. Microbiol.* **9**, 126 (2018).

184. Wang, W. *et al*. Genome-wide characterization of the aldehyde dehydrogenase gene superfamily in soybean and its potential role in drought stress response. *BMC Genomics* **18**, 518 (2017).

185. Sun, T. *et al*. H2O2 mediates transcriptome reprogramming during soybean mosaic virus-induced callose deposition in soybean. *Crop J.* **10**, 262-272 (2022).

186. Usovsky, M. *et al*. Dissecting nematode resistance regions in soybean revealed pleiotropic effect of soybean cyst and reniform nematode resistance genes. *Plant Genome* **14**, e20083 (2021).

187. Chen, L. *et al.* Soybean AP1 homologs control flowering time and plant height. *J. Integr. Plant Biol.* **62**, 12, 1868–1879 (2020).

188. Liu, B. *et al*. The soybean stem growth habit gene *Dt1* is an ortholog of Arabidopsis *TERMINAL FLOWER1*. *Plant Physiol.* **153**, 198–210 (2010).

189. Cai, Z. *et al.* GmTIR1/GmAFB3-based auxin perception regulated by miR393 modulates soybean nodulation. *New Phytol*. **215**, 672–686 (2017).

190. Li, H. *et al.* A genetic relationship between phosphorus efficiency and photosynthetic traits in soybean as revealed by QTL analysis using a high-density genetic map. *Front. Plant Sci*. **7**, 924 (2016).

191. Almeida-Silva, F. & Venancio, T.M. Pathogenesis-related protein 1 (PR-1) genes in soybean: Genome-wide identification, structural analysis and expression profiling under multiple biotic and abiotic stresses. *Gene* **809**, 146013 (2022).

192. Lin, X., Liu, B., Weller James, L., Abe, J. & Kong, F. Molecular mechanisms for the photoperiodic regulation of flowering in soybean. *J. Integr. Plant Biol.* **63**, 981– 994 (2021).
