# Supplementary material for: Selection and adaptive introgression guided the complex evolutionary history of the European common bean
Source: Nat Commun. 2023 Apr 5;14:1908. doi: 10.1038/s41467-023-37332-z (PMC10076260; doi:10.1038/s41467-023-37332-z)
Supplement: Supplementary file 2 — Description of Additional Supplementary Files [file 41467_2023_37332_MOESM2_ESM.pdf]

Description of Additional Supplementary Files

File Name: Supplementary Data 1
Description: List of the common bean accessions used in this study. BEAN_ADAPT code, green (American accessions), blue (European accessions).

File Name: Supplementary Data 2
Description: Summary of data yield and mapping statistics.

File Name: Supplementary Data 3
Description: Summary of genotypes for each sample.

File Name: Supplementary Data 4
Description: Donor accessions included in the ChromoPainter analysis.

File Name: Supplementary Data 5
Description: Ecological data at about 1-km2 resolution, for each accession and for a total of 19 bioclimatic variables (see note below) and 24 monthly variables (precipitations and min, mean and maximum temperatures from April to September). Data were downloaded from WorldClim data (http://www.worldclim.org, Hijmans et al.38).

File Name: Supplementary Data 6
Description: Total amount of synonymous, missense, stop-gain and stop-loss non-reference alleles within each accession.

File Name: Supplementary Data 7
Description: Putative genomic regions target of excess of introgression events. AND-->MES: Andean alleles into the Mesoamerican gene pool. MES--> AND: Mesoamerican alleles into the Andean gene pool. F (AND), F(MES) and F(miss) is the mean observed proportion of Andean, Mesoamerican and missing alleles across the genomic region considering the entire set of European accessions. The number of SNPs supporting each region is reported in the last column.

File Name: Supplementary Data 8
Description: Genomic regions under selection identified by the hapFLK analysis. Regions can be part of the “Extended” or the “Restricted” sets and be located in the top 5% or 1% (FST values).

File Name: Supplementary Data 9
Description: Regions showing strong inter-chromosomal LD levels that were private of the EU_M group. The genomic position (chromosome, start and end position) is reported for both the regions involved in the inter-chromosomal LD.

File Name: Supplementary Data 10
Description: Regions showing strong inter-chromosomal LD levels that were private of the EU_A group. The genomic position (chromosome, start and end position) is reported for both the regions involved in the inter-chromosomal LD.

File Name: Supplementary Data 11
Description: Regions showing strong inter-chromosomal LD levels that were private of the EU_M group, among the “Extended” set of regions under selection. The genomic position (chromosome, start and end position) is reported for both the regions involved in the inter-chromosomal LD.

File Name: Supplementary Data 12
Description: Regions showing strong inter-chromosomal LD levels that were private of the EU_M group, among the “Restricted” set of regions under selection. The genomic position (chromosome, start and end position) is reported for both the regions involved in the inter-chromosomal LD.

File Name: Supplementary Data 13
Description: Putative candidate genes for adaptation according to the presence of signature selection, significant FST between European and American accessions, location within adaptive introgression scan, and/or GWAS peaks for flowering and growth habit traits. Previous studies and information on orthologous genes in soybean (columns Q, R, S), A. thaliana (columns M, N, O), or in common bean (columns D, E) are highlited with the corresponding literature (see the Supplementary References section in the Supplementary Information).
