# Supplementary material for: Selection and adaptive introgression guided the complex evolutionary history of the European common bean
Source: Nat Commun. 2023 Apr 5;14:1908. doi: 10.1038/s41467-023-37332-z (PMC10076260; doi:10.1038/s41467-023-37332-z)
Supplement: Supplementary file 4 — Reporting Summary [file 41467_2023_37332_MOESM4_ESM.pdf]

nature portfolio

Corresponding author(s): Prof. Roberto Papa

Last updated by author(s): Feb 17, 2023

Reporting Summary

Nature Portfolio wishes to improve the reproducibility of the work that we publish. This form provides structure for consistency and transparency in reporting. For further information on Nature Portfolio policies, see our Editorial Policies and the Editorial Policy Checklist.

Statistics

For all statistical analyses, confirm that the following items are present in the figure legend, table legend, main text, or Methods section.

n/a	Confirmed	
☐	☒	The exact sample size (*n*) for each experimental group/condition, given as a discrete number and unit of measurement
☐	☒	A statement on whether measurements were taken from distinct samples or whether the same sample was measured repeatedly
☐	☒	The statistical test(s) used AND whether they are one- or two-sided<br>*Only common tests should be described solely by name; describe more complex techniques in the Methods section.*
☒	☐	A description of all covariates tested
☐	☒	A description of any assumptions or corrections, such as tests of normality and adjustment for multiple comparisons
☐	☒	A full description of the statistical parameters including central tendency (e.g. means) or other basic estimates (e.g. regression coefficient) AND variation (e.g. standard deviation) or associated estimates of uncertainty (e.g. confidence intervals)
☐	☒	For null hypothesis testing, the test statistic (e.g. *F*, *t*, *r*) with confidence intervals, effect sizes, degrees of freedom and *P* value noted<br>*Give P values as exact values whenever suitable.*
☐	☒	For Bayesian analysis, information on the choice of priors and Markov chain Monte Carlo settings
☒	☐	For hierarchical and complex designs, identification of the appropriate level for tests and full reporting of outcomes
☐	☒	Estimates of effect sizes (e.g. Cohen's *d*, Pearson's *r*), indicating how they were calculated

Our web collection on statistics for biologists contains articles on many of the points above.

Software and code

Policy information about availability of computer code

Data collection	A detailed description of all the softwares used for data collection is provided in the supplementary information. Moreover here we provide a list of software for data collection. JMP 7.0.0, GeneData REFINER MS 10.0 (http://www.genedata.com).
Data analysis	A detailed description of all the softwares used for data analysis is provided in the supplementary information. Moreover here we provide a list of software for data analysis. JMP 7.0.0, GeneData REFINER MS 10.0 (http://www.genedata.com), BWA-mem v0.7.15, SAMtools (ref.56. in main manuscript), Genome Analysis ToolKit (GATK) v3.657,58, BCFtools(59),Picard v2.4.1 (http://broadinstitute.github.io/picard), VCFtools(60) , SnpEff v4.3s61 , Beagle(62), ADMIXTURE v1.313, BAPS v5.3(63, 64), MEGA X(65), ChromoPainter v2.0(20), hapFLK(37), fastPHASE v1.4.8, PopLDdecay(72), Orthofinder(76,77) Metascape(78).<br>R (https://cran.r-project.org/) packages; corrplot, PerformanceAnalytics, vegan, imputeqc (https://github.com/inzilico/imputeqc), MVP(https://github.com/XiaoleiLiuBio/MVP), Rcircos.

For manuscripts utilizing custom algorithms or software that are central to the research but not yet described in published literature, software must be made available to editors and reviewers. We strongly encourage code deposition in a community repository (e.g. GitHub). See the Nature Portfolio guidelines for submitting code & software for further information.

Data

Policy information about availability of data

All manuscripts must include a data availability statement. This statement should provide the following information, where applicable:

- Accession codes, unique identifiers, or web links for publicly available datasets
- A description of any restrictions on data availability
- For clinical datasets or third party data, please ensure that the statement adheres to our policy

The raw sequence reads generated and analyzed during the current study are available in the Sequence Read Archive (SRA) of the National Center of Biotechnology Information (NCBI) with the following BioProject number PRJNA573595.
Phytozome v.2.1
P. vulgaris chloroplast genome (NCBI NC_009259)
WorldClim (http://www.worldclim.org)
Whole genomes sequences data from Wu et al.(25, reference in the main manuscript) for Chinese landraces (n=457) (vcfs downloaded from https://zenodo.org/record/3236786#.Y49hDXbMK3A).

A data availability statement including these information is provided within the manuscript.

Human research participants

Policy information about studies involving human research participants and Sex and Gender in Research.

Reporting on sex and gender	N/A
Population characteristics	N/A
Recruitment	N/A
Ethics oversight	N/A

Note that full information on the approval of the study protocol must also be provided in the manuscript.

Field-specific reporting

Please select the one below that is the best fit for your research. If you are not sure, read the appropriate sections before making your selection.

☒ Life sciences ☐ Behavioural & social sciences ☐ Ecological, evolutionary & environmental sciences

For a reference copy of the document with all sections, see nature.com/documents/nr-reporting-summary-flat.pdf

Life sciences study design

All studies must disclose on these points even when the disclosure is negative.

Sample size	The choice of the sample size for this study , that focused on the domestication and introduction in Europe of the two main gene pools of common bean (Andean and Mesoamerican), was made in order to have a wide and balanced representation of American and European accessions for both gene pools, with an almost equal representation between European (n=114) and American (n=104) and Andean (n=51) and Mesoamerican (n=53) accessions in America. The sample size was considered adequate to perform GWAS analysis.
Data exclusions	No data were excluded.
Replication	Nine phenotyping trials were conducted in three locations, with each accession having from three to four biological replicates.<br>In detail, out of the nine trials, 4 were conducted in open field (years 2016 and 2017 both in Italy, UNIBAS and in Germany, IPK), and 5 in greenhouse (2016, 2107 and 2018 in Germany, MPI-MP, and 2016 and 2017 in Itay, UNIBAS). For more detail, see Supplementary Tab. 1 and Supplementary Notes 3-4.<br>In one location (greenhouse; SERIDA) accessions were not replicated, as the esperiment has been conducted in controlled conditions and with limited space. For metabolomics, at least three biological replicates per genotype were used.
Randomization	For phenotyping trials we applied a complete randomized block design. For metabolomics analysis, samples have been processed after samples randomization.
Blinding	For phenotyping trials, phenotyping was performed blindly without knowing the name of the accessions, by labelling plots with progressive numbers. For metabolomics analysis, datafiles were named with a progressive code unrelated to the accession name.

Reporting for specific materials, systems and methods

We require information from authors about some types of materials, experimental systems and methods used in many studies. Here, indicate whether each material, system or method listed is relevant to your study. If you are not sure if a list item applies to your research, read the appropriate section before selecting a response.

Materials & experimental systems

n/a	Involved in the study
☒	☐ Antibodies
☒	☐ Eukaryotic cell lines
☒	☐ Palaeontology and archaeology
☒	☐ Animals and other organisms
☒	☐ Clinical data
☒	☐ Dual use research of concern

Methods

n/a	Involved in the study
☒	☐ ChIP-seq
☒	☐ Flow cytometry
☒	☐ MRI-based neuroimaging
